# Synthetic super-enhancers enable precision viral immunotherapy

**DOI:** 10.1038/s41586-026-10329-6

**Published:** 2026-04-08

**Authors:** Ute Koeber, Mantas Matjusaitis, Neza Alfazema, Katharine Furlong, Zeyu Wang, Rachel White, Alhafidz Hamdan, Pooran Dewari, Gregoire Morisse, Mariela Navarette, Rosie Willis, Jin Wang, Michelle P. Clark, Carla Jacinto de Sousa, Hei Ip Hong, Shahida Sheraz, Ben Southgate, Justyna Cholewa-Waclaw, Sabine Gogolok, Gillian M. Morrison, Felipe Galvez Cancino, Faye Robertson, Anna Williams, Susan J. Rosser, Paul M. Brennan, Dirk Sieger, Abdenour Soufi, Sergio A. Quezada, Steven M. Pollard

**Affiliations:** 1 Centre for Regenerative Medicine, Institute for Regeneration and Repair, University of Edinburgh, Edinburgh, UK; 2 Cancer Research UK Scotland Centre, Edinburgh, UK; 3 Edinburgh Pathology, Royal Infirmary Edinburgh, NHS Lothian, Edinburgh, UK; 4 Institute for Neuroscience and Cardiovascular Research, University of Edinburgh, Edinburgh, UK; 5 Immune Regulation and Tumor Immunotherapy Group, Cancer Immunology Unit, Research Department of Haematology, UCL Cancer Institute, London, UK; 6 School of Biological Sciences, University of Edinburgh, Edinburgh, UK; 7 Centre for Clinical Brain Sciences, University of Edinburgh, Edinburgh, UK; 8 Present Address: IQVIA RDS, Frankfurt am Main, Germany; 9 Present Address: Plurify, Cambridge, UK; 10 Present Address: Trogenix, Edinburgh, UK; 11 Present Address: Laboratory of Immune Regulation, NDM Centre for Immuno-Oncology, University of Oxford, Oxford, UK

**Keywords:** Cancer immunotherapy, Targeted gene repair

## Abstract

Cell-type-specific promoters are used in gene therapy to restrict expression of the therapeutic payload. However, these promoters often have suboptimal strength, selectivity and size. Here, leveraging recent insights into the function of enhancers, we developed synthetic super-enhancers (SSEs) by assembling functionally validated enhancer fragments into multipart arrays. Focusing on the core SOX2-driven and SOX9-driven transcriptional regulatory network in glioblastoma stem cells (GSCs)^1^, we engineered SSEs with robust activity and high selectivity. Single-cell profiling, biochemical analyses and genome-binding data indicated that SSEs integrate neurodevelopmental and signalling-state transcription factors to trigger the formation of large multimeric complexes of transcription factors. Moreover, GSC-selective expression of a combination of cytotoxic (HSV-TK and ganciclovir) and immunomodulatory (IL-12) payloads, delivered using adeno-associated virus vectors, as a single treatment led to curative outcomes in a mouse model of aggressive glioblastoma. Notably, IL-12 induced an immunological memory that prevented tumour recurrence. The activity and selectivity of the adeno-associated virus and SSE were validated using primary human glioblastoma tissue and normal cortex samples. In summary, SSEs harness the unique core transcriptional programs that define the GSC phenotype and enable precision immune activation. This approach may have broader applications in other contexts when precise control of transgene expression in specific cell states is necessary.

## Abstract (web summary)

Synthetic super-enhancers enable specific delivery of anticancer payloads, achieving tumour elimination after a single dose in a mouse model of aggressive glioblastoma.

## Main

Gene therapies require the targeted expression of payloads in specific cell populations to achieve appropriate therapeutic dosing and to minimize off-target effects. Various strategies to attain this selectivity can be explored, including delivery routes, capsid engineering and regulatory elements (for example, promoters, enhancers, untranslated regions and termination signals)^2^. Enhancers determine cell-type-specific expression by recruiting complementary transcription factors (TFs) at high density via TF-binding motifs (TFBMs)^3,4^. However, natural enhancers often have suboptimal features for application in gene therapy vectors, such as size, strength, selectivity and quality of the sequence (for example, GC-rich or repetitive sequences). Strategies for creating artificial promoters and enhancers have typically focused on screening small (around 10 bp) synthetic TFBMs and assembling them into concatamers^5–7^. However, this approach is inadequate owing to the lack of a natural TFBM grammar, that is, the spacing, order, orientation and affinity of TFBMs, which are necessary for cell-type selectivity^8^.

Cell-type-specific enhancers are often clustered in the genome to create super-enhancers^9,10^. Super-enhancers typically regulate transcriptional programs associated with cell identity through the local clustering of low-affinity motifs^8^. This leads to increased high-density binding of lineage and signalling TFs to promote RNA polymerase II recruitment and high transcriptional output^11,12^. We propose that synthetic super-enhancers (SSEs) can be engineered by assembling natural enhancer fragments associated with different genes into multipart arrays and then functionally screening them to identify desired activity and selectivity. The resulting transgene regulatory elements would capture the fundamental grammar of the combinations of TFs that define a specific cell type and signalling state. Such SSEs could have substantial value in gene therapy applications.

We focused on glioblastoma (GBM) to test this platform. GBM is an incurable brain cancer that is driven by cells with fetal neural stem-like phenotypes termed GBM stem cells (GSCs). These cells express high levels of master regulatory TFs associated with neural stem and progenitor cells, including SOX2 (ref. ^13^). SOX2 is a pioneer TF and reprogramming factor essential for inducing and maintaining the identity of neural stem cells (NSCs)^14,15^ and GSCs^1,16–18^. SOX2 is also a crucial master regulatory TF that is broadly expressed across diverse GSC subtypes^18^.

Here we develop SSEs for the targeted expression of anticancer payloads in GSCs. A single, locally delivered dose of adeno-associated virus (AAV)-SSE-7^19^ was evaluated in an immunocompetent model of aggressive GBM. We demonstrate that tumours are safely cleared using this precise and controlled method to express a dual payload of a cytotoxic (herpes simplex virus thymidine kinase and ganciclovir (HSV-TK/GCV)) and a cytokine (IL-12).

## GSC-specific enhancer fragment design

**Fig. 1:**
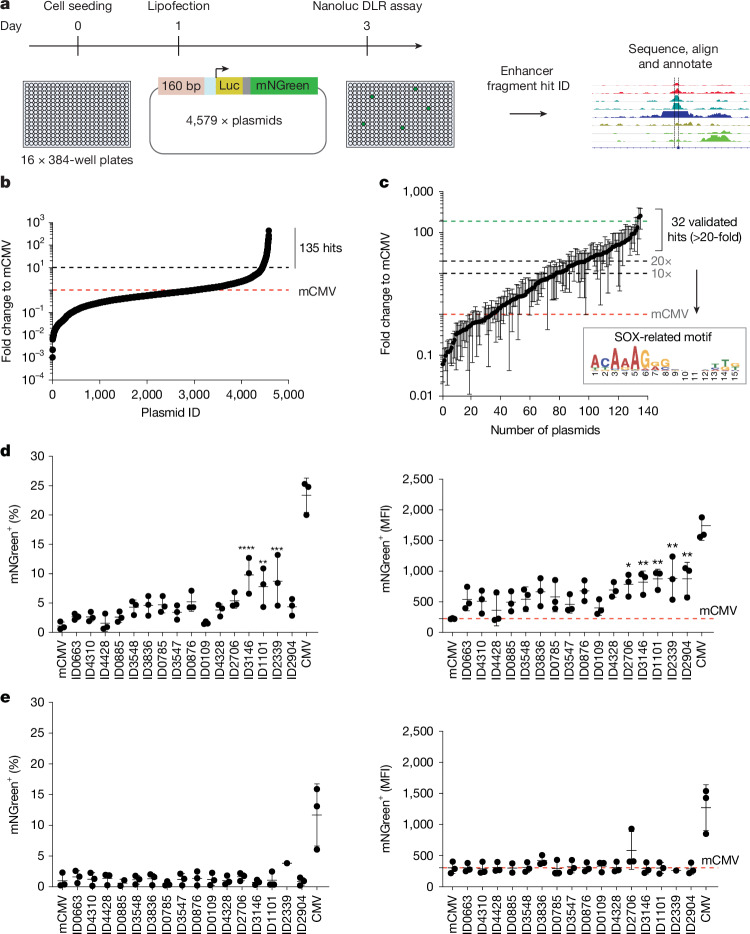
Functional enhancer screening of an arrayed plasmid library. **a**, Strategy for enhancer fragment (160 bp) functional screening using an arrayed library of 4,579 plasmids with luciferase (Luc; yellow) and mNeonGreen (mNGreen; green) reporters (single mRNA linked by *P2A*; grey), with an mCMV promoter (light blue). **b**, Results for the enhancer fragment screen in GSCs (G7 cells) (*n* = 1). **c**, Validation of 135 hits from the primary screen using independent luciferase reporter assays (NanoLuc DLR assay) (*n* = 3 biological replicates, error bars represent the s.d. of the arithmetic mean). The inset shows the enriched SOX dimer motif identified using the MEME tool. **d**, Validation of the top 16 significant hits from **c**, using flow cytometry to determine the percentage of mNeonGreen reporter-positive cells in an independent GSC line (E28), and the MFI per cell for each fragment (right). mCMV and the full-length CMV promoter were used as negative and positive reference controls, respectively (each dot represents a biological replicate (*n* = 3), error bars represent the s.d. of the arithmetic mean). Ordinary one-way analysis of variance (ANOVA) with Dunnet’s multiple comparison test against mCMV was performed. For the mNeonGreen (%) data, adjusted *P* < 0.0001 for ID3146, *P *= 0.0018 for ID1101 and *P *= 0.0003 for ID2339. In the MFI mNeonGreen panel, *P* = 0.0171 for ID2706, *P* = 0.0092 for ID3146, *P* = 0.0038 for ID1101, *P* = 0.0034 for ID2339 and *P* = 0.0037 for ID2904. **e**, Negative control normal human dermal fibroblasts (huFib70) using the same assays as **d** (each dot represents a biological replicate (*n* = 3), error bars represent the s.d. of the arithmetic mean). *P ≤ 0.05, **P ≤ 0.01, ***P ≤ 0.001, ****P ≤ 0.0001. Source data

**Extended Data Fig. 1:**
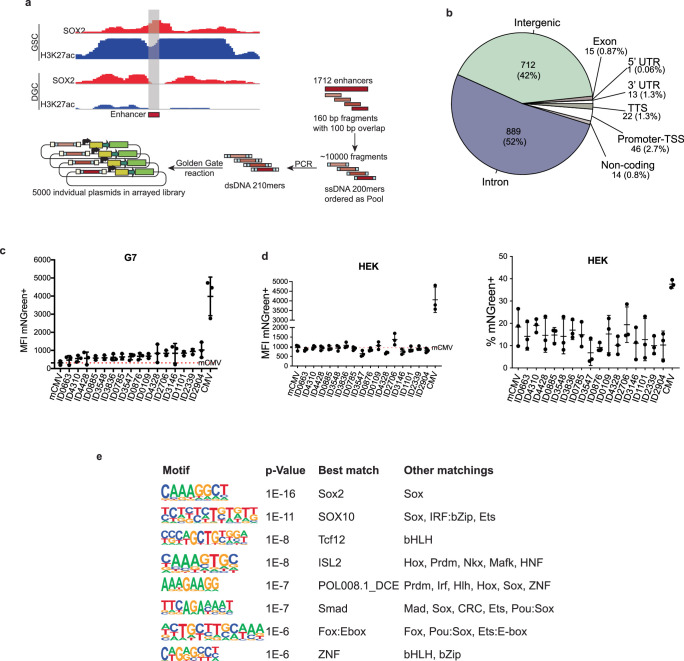
Design of candidate SOX2 GSC-specific peaks and their conversion into a library of candidate enhancer fragments. (a) Summary of the workflow to design and build the enhancer fragment library (DGC: differentiated glioma cell). (b) The genomic annotation of the final library of candidate peaks. (c) The top 16 fragment hits identified from the initial screen (Fig. 1) were validated in GSC7 GSCs for mNeongreen expression using flow cytometry (median fluorescence intensity shown (left). (d) The fragments have the same level of expression in HEK negative control cells as the minimal CMV (MFI and % of total cell population shown, left and right, respectively). (e) De novo motif analysis using HOMER for the Top32 hits of the validation (using shuffled sequences as background) from Fig. 1, confirms the repertoire of candidate TF motifs that are enriched in the functional enhancer fragments. Source data

TFs bind to thousands of sites in the genome, but only a small subset are functional enhancers^20^. To identify candidate GSC-selective enhancers, we first re-analysed previously published datasets^1^ that mapped SOX2-binding sites and used chromatin immunoprecipitation with sequencing (ChIP–seq) to define those specific to GSCs and lost in their differentiated progeny. Of the 1,710 candidate GSC-specific peaks identified (range of 115–3,366 bp, mean of 400 bp), a library of 160-bp double-stranded DNA oligonucleotides, with overlapping 100 bp segments, was synthesized (Extended Data Fig. 1). The 160 bp length was chosen as this was deemed to be the optimum size to capture the core set of TFBMs with the necessary natural grammar and motif diversity that might confer selectivity. Moreover, this length is compatible with nucleosome formation for pioneer factors such as SOX2 (ref. ^21^). Candidate fragments were amplified by PCR, cloned into a luciferase–mNeonGreen reporter plasmid and assembled into an arrayed plasmid library (48 × 96-well plates with 4,579 individual plasmids) (Fig. 1a).

## Discovery of functional GSC enhancers

Patient-derived GSCs can be routinely propagated in a monolayer and can be efficiently transfected^13^. We screened the enhancer fragment plasmid library in GSCs (specifically GSC7 cells) using a luciferase assay to identify functional hits^22^. From this initial screen, 135 plasmids exhibited more than 10-fold activity over a minimal CMV (mCMV) promoter (Fig. 1b). Several hits were identified recurrently, which provided confidence in the screening assay.

Sanger sequencing confirmed sequences of the 135 initial hits, and 32 unique sequences were validated in triplicate with >20-fold activity compared with mCMV (Fig. 1c, Extended Data Table 1 and Supplementary Table 1). The top 16 candidates were also validated using an independent flow cytometry assay to detect the mNeonGreen transcriptional reporter, which provides single-cell quantification of activity and mean fluorescence intensity (MFI). All 16 validated hits were active in an independent patient-derived GSC line (E28), but were inactive in fibroblasts and HEK293 cells, which were used as SOX2-negative controls (Fig. 1d,e and Extended Data Fig. 1c,d). As anticipated, these hit sequences contained TFBMs for many known neurodevelopmental or GBM-associated TF families, including motifs for SOX, bHLH, FOX, TCF and SMAD (Extended Data Fig. 1e).

Notably, de novo motif searches using the MEME tool revealed that the 32 active fragments contained a specific SOX dimer motif that resembles a previously reported SOX9 motif^23^ (Fig. 1c). Although SOX2 typically operates as a monomer, SOX9—a known NSC self-renewal factor^24^—can function as a homodimer^23^. This result suggests that the functional enhancer fragments potentially bind SOX9.

**Extended Data Fig. 2:**
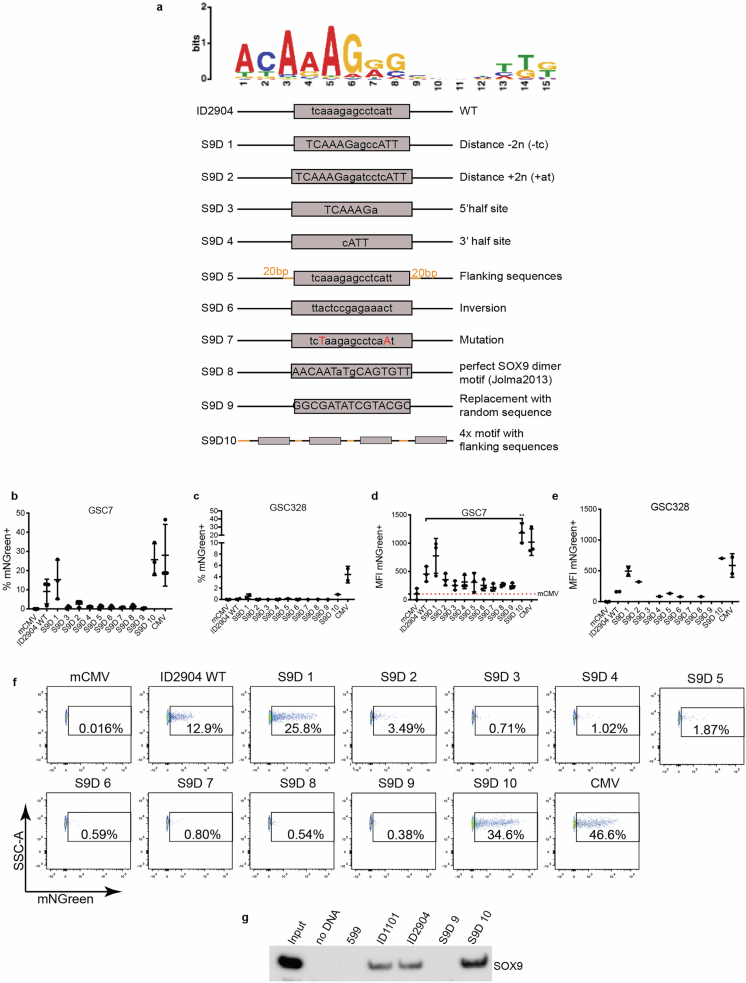
A dimeric SOX motif that binds SOX9 is a major contributor to activity. (a) A specific dimeric SOX dimer motif was identified using MEME for de novo motif discovery and is enriched in the set of functional enhancer hits (ACAAAGRGBVNHTKK) and was mutated as depicted (below) for 10 different variants of the ID2904 sequence to determine the functional impact. (b-e) Plasmid-based flow cytometry assay to determine the activity of each variant, in GSCs (GSC7 and E28, respectively). Interestingly, increased activity was seen with 2 bp reduction of the spacing consistent with the importance of this motif for activity. Each dot represents a biological replicate (2e) One-way Anova test with Dunnett’s multiple comparisons test against ID2904 WT; ** represent p-value of 0.0040 (f) Examples of flow cytometry data used for panels c-f. M(f) Examples of flow cytometry data used for panels c-f. (g) SOX9 protein selectively interacts with ID1101 and ID2904 through the dimer motif. Source data

To functionally validate the importance of this motif in the enhancer fragments, we created a series of mutants in the dimer motif of ID2904 (which was chosen because it only has a single motif) with nucleotide substitutions, inversions or changes in spacing. Each of these alterations abolished the transcriptional activity of the fragment (Extended Data Fig. 2a–f). Higher activity was observed when the distance between the half-sites was reduced by 2 bp. This result suggests that dimer binding is a crucial feature of the activity of the motif and is probably important for SOX9 binding. Indeed, using biochemical pull-down, we confirmed that SOX9 binds to ID2904 and that binding depends on the SOX dimer motif (Extended Data Fig. 2g).

**Fig. 2:**
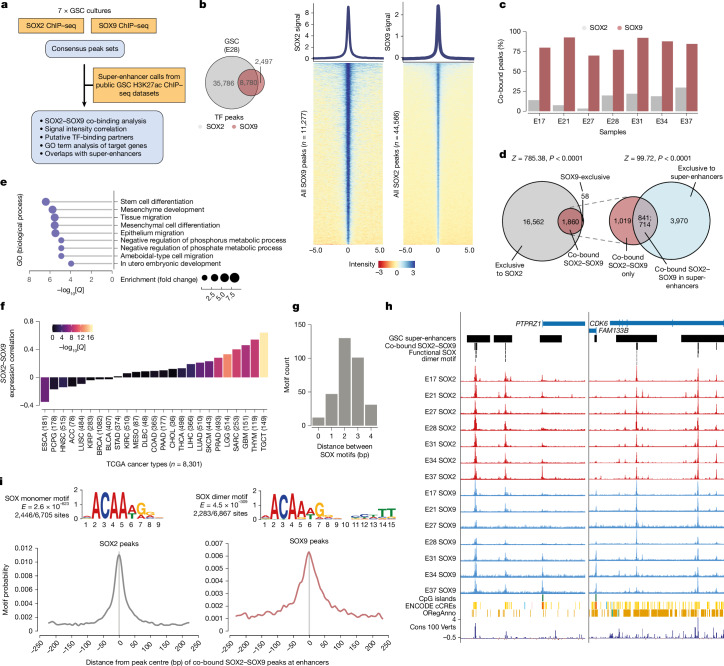
ChIP–seq analysis of SOX2 and SOX9 binding in seven different patient-derived GSC cultures. **a**, Schematic of SOX2 and SOX9 ChIP–seq experimental design and analysis. GO, gene ontology. **b**, Venn diagram showing the overlap between SOX2 and SOX9 peaks for the sample E28 (left), and heatmaps of SOX2 and SOX9 binding intensity over SOX9 and SOX2 peaks, respectively (right), demonstrating co-binding events. **c**, Bar plots showing the proportion of SOX9 peaks located in co-bound sites. **d**, Venn diagrams showing the overlaps between consensus SOX2 and SOX9 peaks and co-bound SOX2–SOX9 peaks with a consensus set of GSC super-enhancers (*n* = 44 publicly available GSC H3K27ac datasets; Methods). **e**, Gene ontology analysis of co-bound SOX2–SOX9 peaks in super-enhancers. **f**, Bar plot showing the distribution of *SOX2–SOX9* expression correlation values (*y *axis) across diverse cancer types from The Cancer Genome Atlas (*x* axis). **g**, Bar plot showing the motif co-occurrences of inverted palindromic SOX motifs, grouped by the distances between the primary and secondary SOX motif pairings. **h**, UCSC genome browser coverage tracks of SOX2 and SOX9 ChIP–seq in the seven individual GSCs at *PTPRZ1* and *CDK6* loci, annotated by the presence of GSC super-enhancers, co-bound SOX2–SOX9 peaks and the functional SOX dimer motif. cCREs, candidate *cis*-regulatory elements; Cons 100 Verts, conservation across 100 vertebrates. **i**, The distributions of centrally enriched de novo SOX monomer and dimer motifs at SOX2 and SOX9 peaks, respectively, restricted to those in co-bound SOX2–SOX9 enhancers.

The above findings led us to speculate that SOX2 and SOX9 might work together at shared target enhancers in GSCs. To find their putative shared target sites, we mapped genome-wide binding of SOX2 and SOX9 in seven independent GSC patient-derived cell lines using ChIP–seq (Fig. 2a). Most SOX9 peaks co-bound with SOX2, and these overlapped with previously reported super-enhancers found across GSCs (Fig. 2b–d). These co-bound peaks were located near genes involved in stem cell function (Fig. 2e), including essential regulators of neural stem and progenitor cells (for example, *CDK6* and *PTPRZ1*; Fig. 2h). The expression of *SOX2* and *SOX9* was positively correlated, particularly in GBM (relative to most other cancers), which implied that these two factors have a disease-specific function and/or regulatory interactions (Fig. 2f). Notably, the SOX2 and SOX9 peaks in co-bound sites at enhancers were enriched with multiple SOX monomer and dimer motifs, respectively, with an optimal dimer motif spacing of 2–3 bp (Fig. 2g,i). Altogether, these data indicate a specific requirement for SOX9 dimerization at loci in which SOX2 and SOX9 are co-bound.

In summary, using functional screening in patient-derived GSCs, we identified candidate 160 bp SOX2 and SOX9 co-bound enhancer fragments with high selectivity but low activity (relative to CMV). Together with previously published findings, such as genome-wide CRISPR screens^18^, these results highlight the importance of SOX2 and SOX9 as core components of a TF circuit that determines and sustains GSC identity. Moreover, they share crucial transcriptional targets.

## SSEs for GBM

**Fig. 3:**
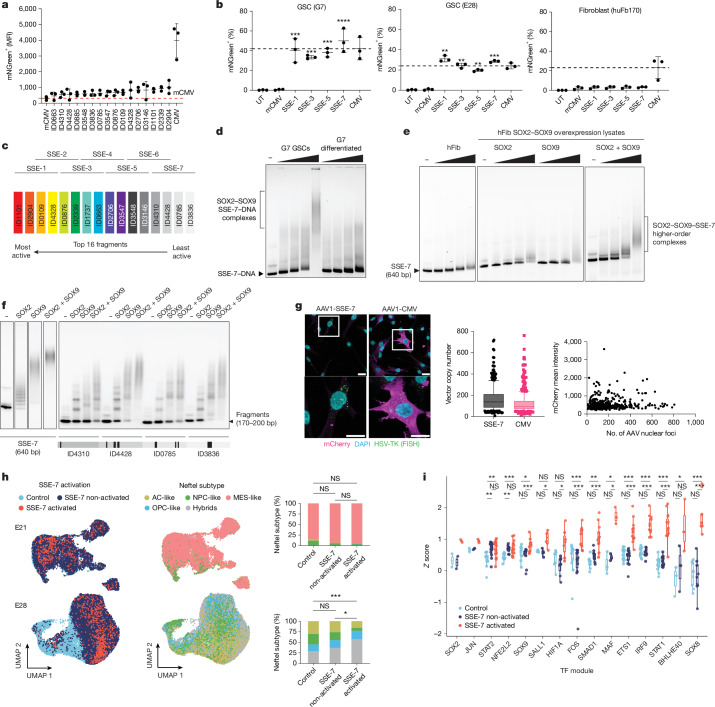
Combining SOX2 functional enhancer fragments results in synergistic increases in activity without compromising selectivity. **a**, Activity of the 16 top hits relative to mCMV and CMV assessed by flow cytometry in GSCs (G7 cells) (each dot represents a biological replicate, error bars represent the s.d. of the mean). **b**, SSE activity detected using an mNeonGreen reporter across GSC lines (GSC7 and E28) and control fibroblasts. Error bars represent the s.d. of the mean. One-way ANOVA with Dunnett’s multiple comparisons test against mCMV, adjusted *P *values for G7: *P* = 0.0001 for SSE-1, *P *= 0.0009 for SSE-3, *P *= 0.0003 for SSE-5 and *P* < 0.0001 for SSE-7. Adjusted *P *values for E28: *P* = 0.0099 for SSE-1, *P* = 0.0048 for SSE-3, *P* = 0.0075 for SSE-5 and *P* = 0.0008 for SSE-7. UT, untreated. **c**, Schematic of each SSE-1, SSE-3, SSE-5 and SSE-7 transgene design. **d**, EMSA of SSE-7 DNA following incubation with GSC lysates compared with differentiated GSCs (representative image;* n* = 3). **e**, EMSA showing SSE-7 DNA with fibroblast (hFib) lysate (left) and hFib with SOX2 and SOX9 expressed individually (middle) or in combination (right). Representative images shown (*n* = 3). **f**, EMSA of SSE-7 DNA (left, representative images of *n* = 3) and its constituent fragments (right, *n* = 1), incubated with SOX2 and SOX9. Schematic of SOX motifs (thin black bar, partial SOX motif; thicker, SOX motif; thickest, dimer motif) is shown underneath the images. **g**, SABER–FISH analysis of transgenes. Representative images are shown (left), with quantification (right). For SSE-7, mean copy number = 167 (median, 119; Q1–Q3, 76–209; min–max, 8–806). For CMV, mean copy number = 115 (median, 87; Q1–Q3, 57–127; min–max, 5–761). Scale bars, 20 μm. **h**, scRNA-seq analysis shows no correlation between SSE-7 activity transcriptional subtypes (AC, astrocyte; MES, mesenchymal; NPC, neural progenitor cell). Neftel subtype, subtype as identified by Neftel et al.^27^. Distribution of SSE-7–mCherry^high^ cells using UMAP plots of scRNA-seq analysis in E21 cells (*n* = 23,384 cells) and E28 cells (*n* = 35,879). **i**, SCENIC analysis to determine TF enrichment across replicate libraries. Combined *Z* scores from each library. *P ≤ 0.05, **P ≤ 0.01, ***P ≤ 0.001; NS, not significant. *P* values were calculated using repeated-measures ANOVA, followed by Bonferroni-adjusted pairwise comparisons of estimated marginal means. Boxes show the interquartile range, centre lines the median, and whiskers the minimum and maximum values. Source data

Our goal was to create a GSC-selective transcriptional switch for cancer gene therapy that combines strong expression and high selectivity. Such a SSE would provide the necessary therapeutic window to target the tumour but spare surrounding normal CNS tissue. We proposed that combining functional and selective enhancer fragments into a larger artificial multipart assembly might increase activity by facilitating increased high-density recruitment of SOX2 and SOX9 alongside other signalling-associated transcriptional cofactors to mimic the mechanism of action of natural super-enhancers^11,12^. We chose four-part assemblies, as this represents an optimal size compatible with AAV vector applications. These assemblies were constructed using sequences from the top 16 validated hits grouped into four sets: 1–4, 5–8, 9–12 and 13–16 (Fig. 3a,c).

Each variant displayed strong activity that matched or exceeded that of a full-length CMV promoter in GSCs. By contrast, the variants had low activity in both fibroblasts and HEK293 cells, such that levels were comparable to mCMV-like levels (Fig. 3b and Extended Data Fig. 4a). Given the principles that underpin their design, their high activity (switch-like behaviour) and their high cell-type selectivity, we herein refer to these artificial elements as SSEs.

To initially explore selectivity in glial cell lineage differentiation, we evaluated SSE activity during GSC differentiation into astrocyte-like cells^1,13^. We observed a twofold reduction in MFI per cell within 24 h, followed by an approximately tenfold decrease by day 10 of differentiation (Extended Data Fig. 4b,c). By contrast, the constitutive CMV promoter displayed high activity across all conditions. SSE activity is therefore highest in the immature stem cell-like state of GSCs and is rapidly reduced as cells differentiate.

**Extended Data Fig. 3:**
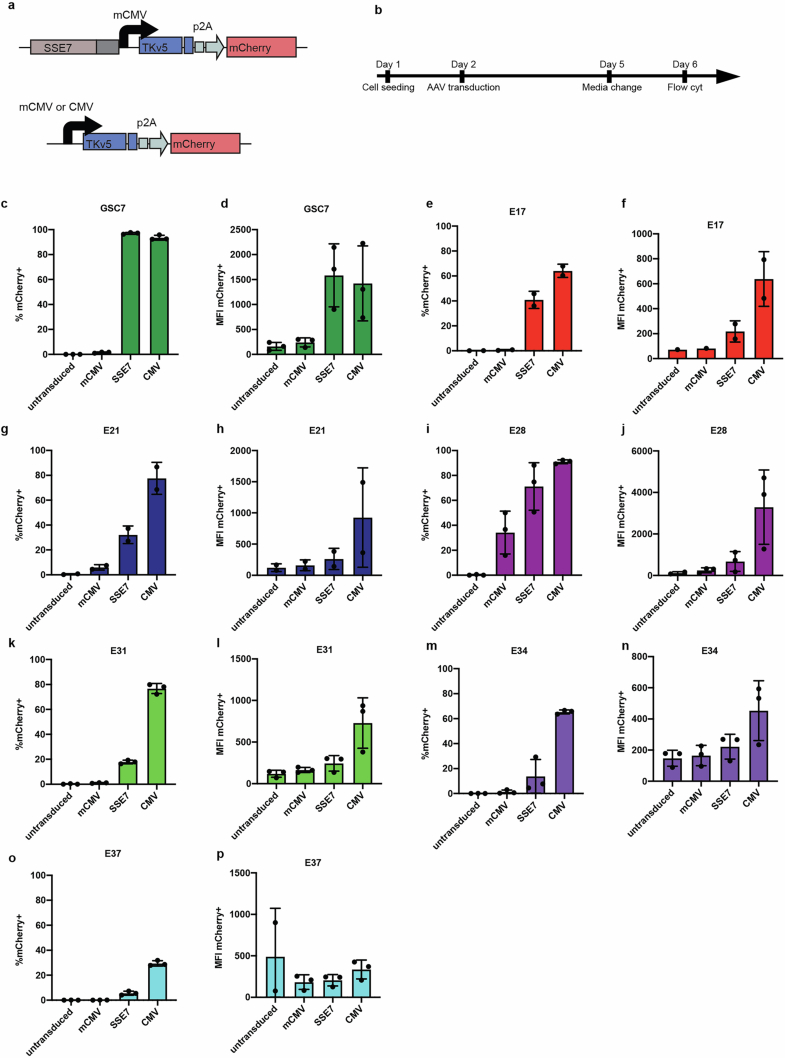
Assessment of SSE-7 activity across a set of seven different patient-derived GSC lines. (a) Reporter reference controls were used to determine relative SSE activity by flow cytometry across each cell line, with minimal CMV (mCMV) and CMV as negative and positive controls, respectively. (c, e, g, i, k, m and o) shows the % mCherry positive cells, whereas (d, f, h, j, l, n and p) shows the median fluorescence intensity (MFI) for each cell. E37 is an outlier line and has an atypical MYC amplification and lower levels of SOX2. Error bars represent the SD of the mean, each dot represents a biological replicate. Source data

Focusing on SSE-7, we next tested a larger set of patient-derived GSCs that span diverse genetic and transcriptional subtypes^25,26^. SSE-7 was active across all seven patient-derived GSCs but had reduced levels in E37 (an atypical cell line with *MYC* amplifications and reduced SOX2 and SOX9 levels) (Extended Data Fig. 3). However, there was variability across the cell lines in the percentage of positive cells in the population compared with CMV, which indicated heterogeneity in the cultures. This result was not explained by SOX2 or SOX9 protein levels, as these were similar across lines, which suggests that additional cooperating TFs might be necessary for SSE-7 activity.

We next explored the biochemical mechanism of the switch-like behaviour and selectivity of SSE-7 and the roles of SOX2 and SOX9 in this process. To that end, we first used cell-free electrophoretic mobility shift assays (EMSAs) to analyse their interactions with SSE-7 alone and in combination using both cell lysates and cell-free recombinant SOX proteins.

SSE-7 incubation with GSC cell extracts resulted in a major super-shift and the formation of large higher-order multimeric complexes, but this was not observed in GSCs that had differentiated into astrocytes (Fig. 3d). Using human fibroblasts with SOX2 and/or SOX9 forced expression, we also observed a clear super-shifted band only when both factors were incubated together with SSE-7, but this effect was weaker compared with GSCs. This result highlights the importance of GSC-associated cofactors for triggering SSE activity (Fig. 3e).

**Extended Data Fig. 4:**
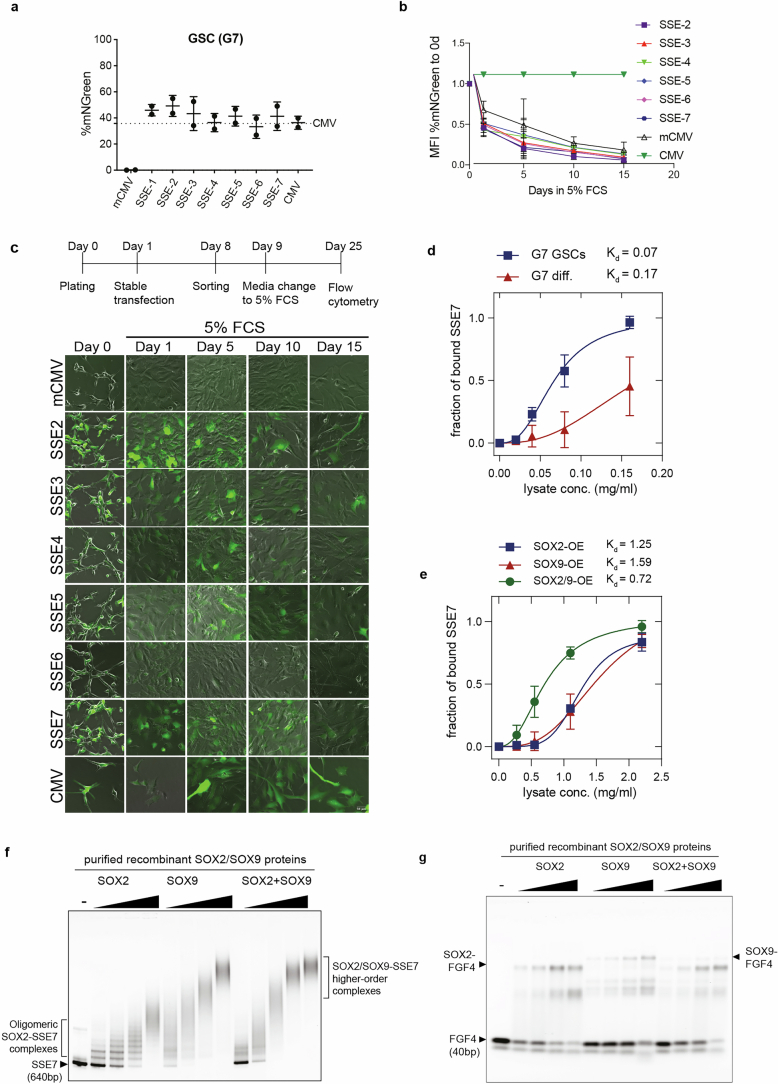
Characterisation of SSEs. (a) Comparison of activity from all seven SSE variants in GSC7 using flow cytometry to monitor expression of the mNeonGreen reporter. Each dot represents a biological replicate. (b) Time-course of GSC astrocyte differentiation to monitor SSE activity using flow cytometry. (n = 2 biological replicates, each dot symbol represents the mean, the error bars represent the SD of the mean) (c) Live cell wide-field immunofluorescence imaging for the cells assessed in (b). (d-e) Quantitation of the band intensities from the EMSA experiments in Fig. 2d,e. Data points are mean from n = 3 with error bars of the SD. Solid line represents the Hill equation with the Kd values displayed above the graph. (f) EMSA of SSE-7 DNA using purified recombinant SOX2 and SOX9, alone or in combination (representative image of n = 3). (g) EMSA showing the individual and combinatorial binding of purified recombinant SOX2 and SOX9 to FGF4 enhancer element (40 bp, n = 1) containing a typical SOX2 motif. Source data

Next, to validate whether synergy exists with SOX2 and SOX9 co-binding, we used purified recombinant SOX2 and SOX9 proteins and tested their binding. When SOX2 alone was bound with SSE-7, it displayed a typical ladder-like banding pattern indicative of SOX2 binding individually at each motif in the 640-bp-long SSE-7 (Fig. 3f and Extended Data Fig. 4d–f). Notably, the binding of SOX9 led to the formation of the higher-order complexes, and this effect was enhanced in the presence of SOX2 (Fig. 3f). The analysis of each constituent 160 bp enhancer fragment in isolation demonstrated that the formation of higher-order complexes depends on the architecture of the SSE (Fig. 3f). No higher-order complexes were formed when using a control 40 bp FGF4 enhancer that contains a single SOX motif (Extended Data Fig. 4g). We therefore conclude that a key feature of the mechanism of action of SSE-7 is its ability to induce higher-order TF complexes through high-density local recruitment of SOX2 and SOX9.

## Mechanistic basis of SSE activity in GSCs

We next aimed to identify candidate SOX-cooperating TFs and the cofactors required for the SSE switch-like transcriptional activation seen in GSCs. To that end, we performed single-cell RNA sequencing (scRNA-seq) analysis of 2,711 SSE-7–mCherry-positive cells across 8 different GSC lines, comparing these to matched mCherry-negative populations. We first used the single-molecule imaging technique signal amplification by exchange reaction and fluorescence in situ hybridization (SABER–FISH) and confirmed that transduction rates and nuclear transgene copy number were similar among the different cell lines and that there was no correlation with mCherry levels (Fig. 3g). Therefore, differential transduction or nuclear processing of the AAV does not explain the restricted SSE-7 activity observed in heterogeneous GSC cultures compared with CMV.

**Extended Data Fig. 5:**
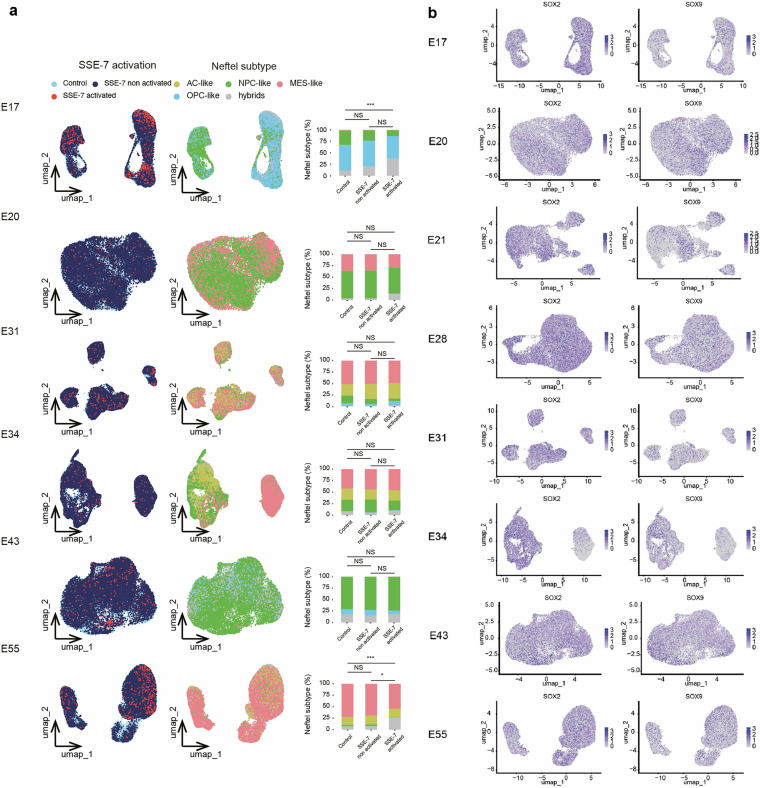
Single cell mRNA profiling of multiple patient derived GSC lines following transduction with AAV1-SSE-7-mCherry virus. (a) Distribution of transcriptional subtypes in scRNA data. UMAP of transcriptional subtype in E17 (n = 29,829 cells), E20 (n = 23,301 cells), E31 (n = 39,823 cells), E34 (n = 27,766 cells), E43 (n = 31,538 cells), E55 (n = 47,291 cells). *P < 0.05, **P < 0.01, ***P < 0.001; NS, not significant. (b) SOX2 and SOX9 are broadly expressed across the diverse transcriptional subtype cell states in GSCs, consistent with their roles as core regulators. See also Extended Data Fig. 6 for cell cycle analysis.

**Extended Data Fig. 6:**
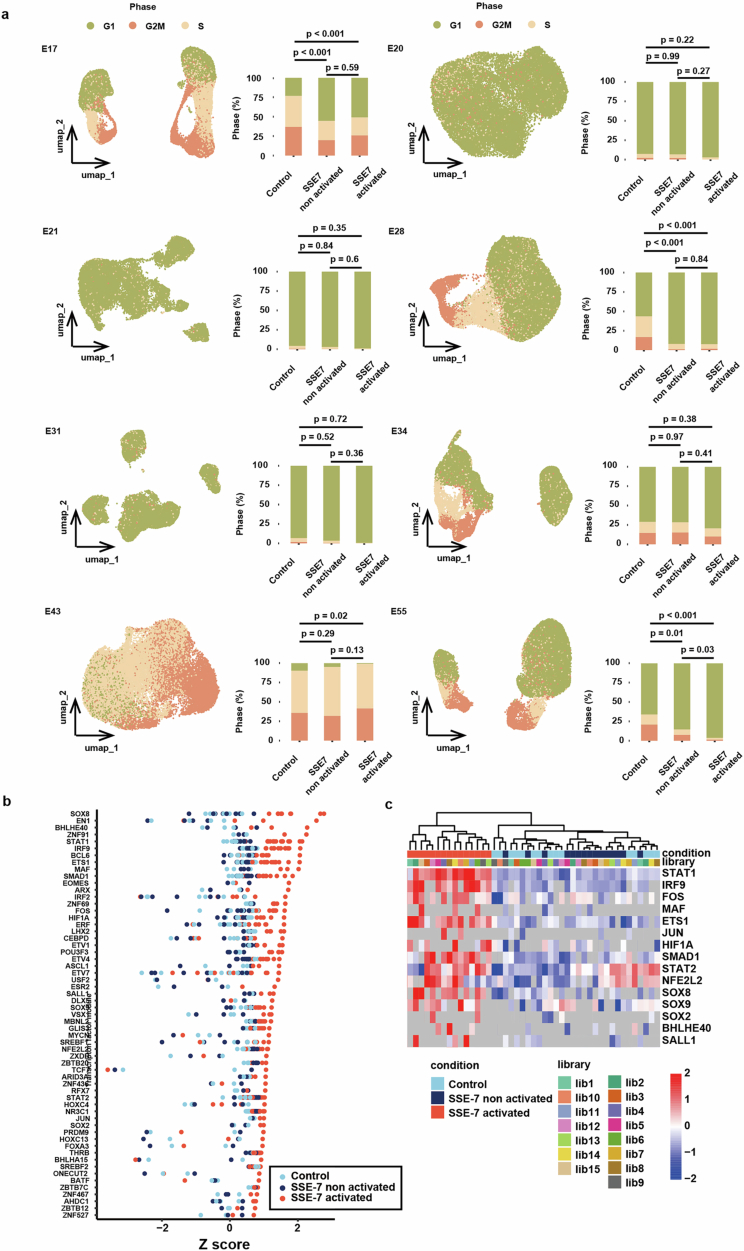
Single cell analysis of cell cycle and SCENIC analysis. (a) Distribution of cell cycle phases across GSC cell states in scRNA data (as per Extended Data Fig. 5). (b) Full data for SCENIC analysis of transcription factor modules identifies signalling endpoint transcription factors alongside SOX as correlating with SSE-7 activity. (c) Heatmap across technical replicates of libraries from Fig. 3i.

Uniform manifold approximation and projection (UMAP) analysis confirmed that these GSC cultures have, as expected, a diverse range of transcriptional subtypes or states^27^. This transcriptional heterogeneity mirrored that seen in patient tumour samples and reflected distinct differentiation states (Fig. 3h). We did not find a simple correlation of SSE-7^high^ activity with previously reported transcriptional subtypes (Fig. 3h), SOX2 and SOX9 expression or cell cycle phase (Extended Data Figs. 5 and 6a).

To perform an unbiased search for TF activities enriched in SSE-7^high^ cells, we used the analysis tool SCENIC. The results revealed a clear correlation of multiple TF activities linked to cancer signalling pathways, including STAT, IRF, SMAD and FOS–JUN (Fig. 3i). Such signalling end-point TFs are often highly activated in cancers, including GBM, and motifs for such TF families are present in SSE-7. This finding is consistent with the idea that these TFs have a potential functional role (Fig. 3i and Extended Data Fig. 6b,c). These data suggest that SSE-7 is activated in cells that display a combination of activated signalling end-point TFs and an immature stem cell-like identity driven by SOX2 and SOX9.

**Extended Data Fig. 7:**
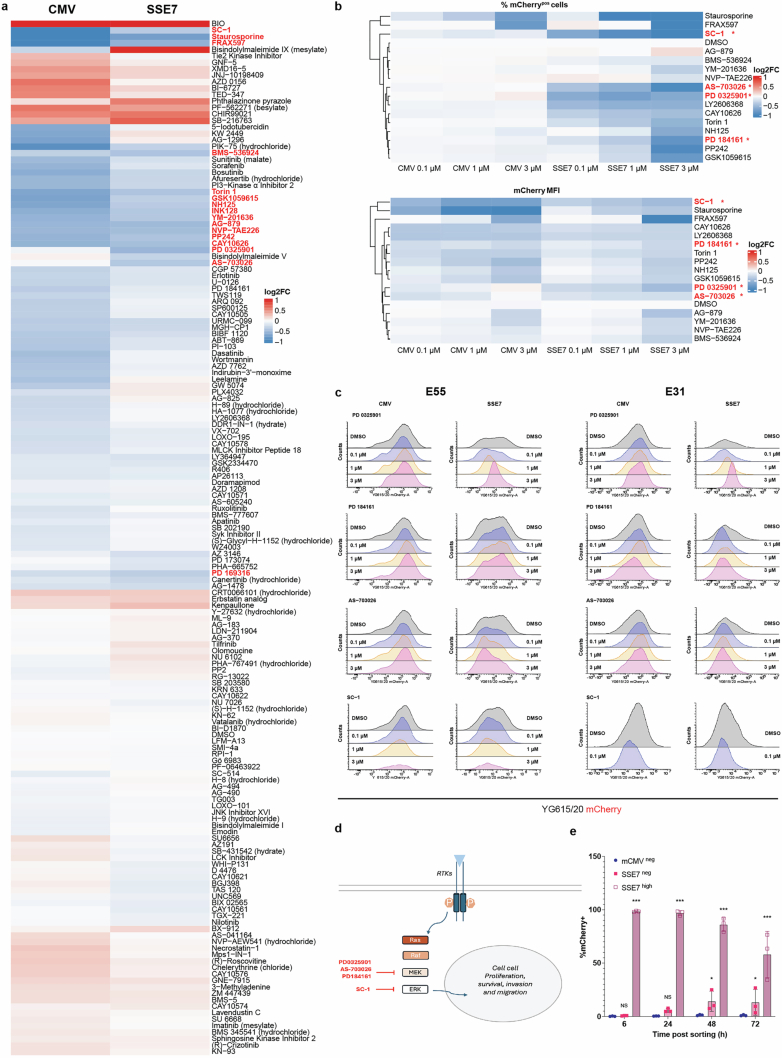
A screen of small molecule pharmacological inhibitors to identify signalling pathways that may impact the activity of SSE-7 in E55 GSCs. (a) The primary screen was performed in the E55 cells. Heatmap summarising the fold change of the normalised mCherry MFI in inhibitor-treated samples compared with DMSO. Initial hits identified with red font. The relative comparator is the CMV-SSE7-mCherry positive control. Any reduction in mCherry selectively in the E55 is due to the different properties of the SSE7. (n = 2 biological replicate; independent screens) (b) Validation of the hit compounds using flow cytometry for mCherry detection across three different concentrations of compound (0.1, 0.3 or 3μM). The heatmap shows the fold change of the normalised mCherry MFI and percentages of mCherry positive cells of inhibitor-treated samples relative to the DMSO-treated control. (n = 2 biological replicates). Total % mCherry cells (top panel) and mean fluorescence intensity (MFI) bottom panel. Red font are MAPK signalling-related compounds validated with dose response and show selective effects on the SSE-7 compared to CMV promoter. (c) Flow cytometry histograms for each validated hit compound. Typical example of flow cytometry overlay histograms for E55 GSCs treated with 3 different doses of MAPK signalling-related inhibitors (left panels) and E31 GSCs (right panels). E55 and E31 have distinct transcriptional subtype identities (mesenchymal and developmental/proneural, respectively). (d) Summary of validated inhibitor hits (red font) and their mechanism of action within the MAPK signalling pathway. (e) Flow cytometry analysis of positive and negative SSE-7mCherry cells sorted and reanalysed over a 72-hr time-course shows that SSE-7 activity can be activated in cells that are initially negative, indicative of a readout that is sensitive to signalling state. n = 3 biological replicates. 2-way ANOVAs followed by two stage setup method were carried out to compare with mCMV ^neg^. *P < 0.05, **P < 0.01, ***P < 0.001; NS, not significant. Note: SC-1 was toxic at the higher doses for E31 and so could not be assessed. Source data

We next explored the requirements needed for signalling pathway activation for SSE-7 function. We performed a screen of 160 different small-molecule kinase inhibitors, spanning 70 distinct families, to identify those that would reduce SSE-7 activity (Extended Data Fig. 7a). Given the diverse signalling states across GSCs and the results from the SCENIC analysis, we speculated that activity would be highly dependent on the cell line. We therefore focused the screen on E55 and E31, two GSC lines with typical phenotypic heterogeneity and distinct transcriptional states.

Initial hits were validated, and a clear dose-dependent reduction in mCherry MFI was confirmed for four of these hits (Extended Data Fig. 7b,c). These four inhibitors all operate in the MAPK–ERK signalling pathway: three MEK inhibitors (PD0325901, AS-703026 and PD184161) and one ERK inhibitor (SC-1) (Extended Data Fig. 7d). For these specific GSC lines (E55 and E31), SOX2 and SOX9 activity together with hyperactive MAPK signalling seemed to be necessary for SSE-7 activity. Finally, a subpopulation of sorted SSE-7-negative GSCs could reactivate SSE-7 expression at later time points. This result is consistent with the finding that SSE activity depends on a specific signalling state (Extended Data Fig. 7e).

Altogether, these functional genetic, biochemical and genomic data indicate that SSE-7 is a dynamic transcriptional regulatory element that is strongly activated in specific GSC states that have a combination of both SOX2 and SOX9 expression and hyperactive signalling-associated TFs. The specific signalling TFs involved are likely to vary among the heterogeneous GSC genetic and transcriptional subtypes. However, our SSE design strategy (that is, mixing distinct enhancer fragments from diverse genes) enabled us to achieve robust and strong expression across GSCs regardless of this variability.

## Cell-state-specific activation of SSE-7

**Extended Data Fig. 8:**
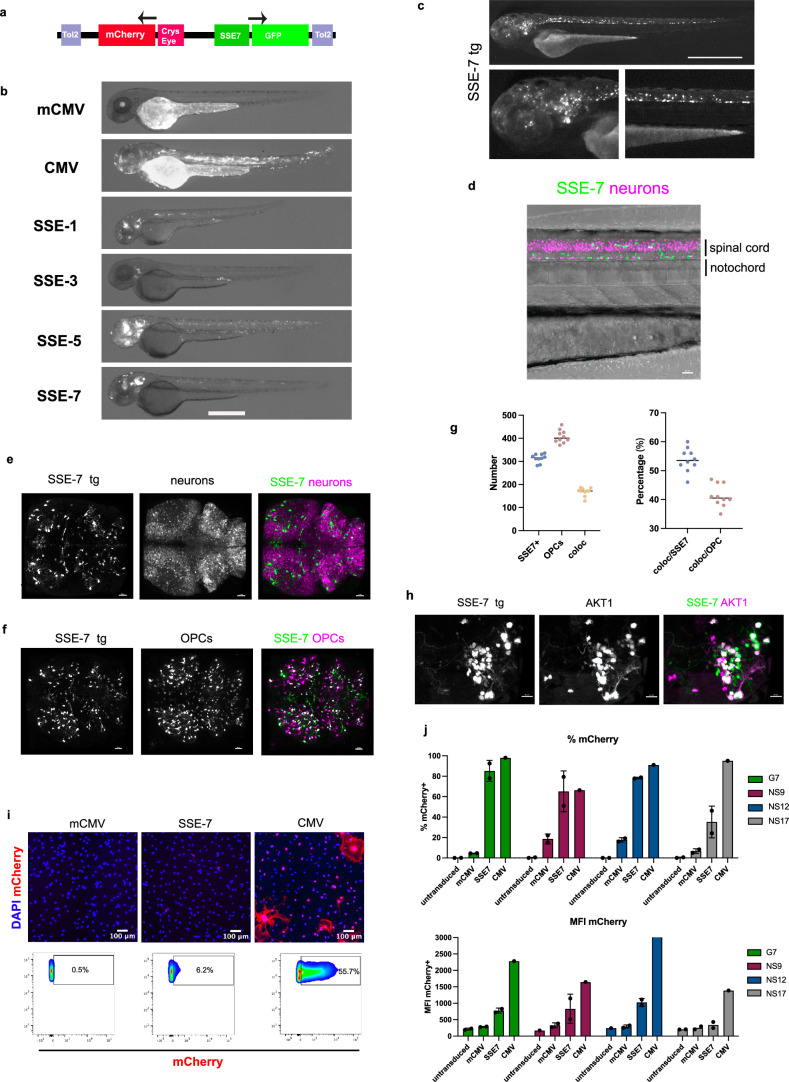
Selectivity in tissue expression in transgenic zebrafish embryos. (a) Reporter transgene for selective expression of SSEs in a zebrafish transgenic reporter model with a GFP reporter, with a transposase (Tol2) reporter designed track transgene insertion using mCherry expression in the eye from the Crystal promoter. (b) SSE activity is tracked by eGFP levels in live cell imaging of the embryos at 48hpf and is tissue-restricted compared parallel CMV controls for each SSE tested. Note: the minCMV control exhibited GFP-expression in the eyes due to spurious transcription from the Crystal eye promoter. (c) A stable transgenic SSE-7:eGFP reporter zebrafish line confirms expression is restricted to the developing nervous system. Images were acquired using a Leica MS205 stereomicroscope. Scale bars represent 500 µm for (b) and 728 µm for (c). (d) Higher magnification image of the spinal cord and nototchord with neuronal reporter (Cyan, as per (e)). (e) Outcross of tgSSE-7:eGFP to tg(Xla.Tubb:DsRed) reveals no expression in neurons; in (f) outcross of tgSSE-7:eGFP to tg(olig1:mScarlet) shows expression in a subset of oligodendrocyte precursor cells. Images represent maximum intensity projections of confocal stacks (optic tectum and cerebellum shown). Images were captured using a Leica SP8 confocal microscope with a 40X/NA 1.1 objective. Scale bars represent 30 µm. (g) Quantification of the OPC data from (f). (h) Overexpression of human Akt1 in neuronal cells results in strong activity of SSE-7. Images represent maximum intensity projections of confocal stacks. Images were captured using a Leica SP8 confocal microscope with a 40X/NA 1.1 objective. Scale bars represent 30 µm. (i) Immunostaining and flow cytometry (top and bottom, respectively) of human ESC differentiated oligodendrocyte progenitor cells shows that SSE-7 is not active compared to CMV (n = 1). (j) SSE7 versus CMV across a set of three human foetal neural stem cell (NSC) cultures (derived from week of gestation 9, 12 and 17 tissue; NS9FB_B, NS12ST_A and NS17ST_A, respectively). Each dot represents a biological replicate. Error bars represent the SD of the mean. Source data

Our functional enhancer fragments typically had high vertebrate evolutionary conservation, which suggested that SSEs may work in different species. To further explore selectivity across diverse tissues and organs, we first tested SSE activity in developing zebrafish (around 48 h after fertilization) (Extended Data Fig. 8). Overlapping expression domains for each SSE-driven eGFP reporter was observed for all four SSEs tested, with consistently high levels of expression in the optic placodes and a subset of forebrain and spinal cord neural progenitors (Extended Data Fig. 8a,b). This restricted developmental expression pattern was consistent with previously reported zebrafish Sox2 and Sox9 expression^27^^,28^, and confirms that not only are the SSEs highly active but they are also highly tissue restricted. Notably, SSE-1, SSE-3 and SSE-5 had an additional weaker expression domain in more posterior CNS tissues, whereas for SSE-3, we detected expression in the posterior endoderm (Extended Data Fig. 8b). We therefore prioritized SSE-7 for subsequent experiments.

We created a stable transgenic zebrafish line and used this to look at later larval stages in the nervous system (Extended Data Fig. 8c). SSE-7 activity was nonoverlapping with mature neuronal markers, which confirmed that SSE-7 is not expressed in neurons and is only seen in a subset of fetal neural progenitor cells (Extended Data Fig. 8d–g). Notably, when we forced the expression of a potent oncogenic form of Akt in zebrafish, we observed that SSE-7 activity increased in those cells. This result is consistent with the requirement of hyperactive signalling together with Sox expression to support SSE activation, as seen in human GSCs (Extended Data Fig. 8h). SSE-7 had low activity in oligodendrocyte progenitor cells (OPCs) derived from human induced pluripotent stem (iPS) cells relative to CMV (Extended Data Fig. 8i), but was expressed in human fetal NSC cultures (Extended Data Fig. 8j). Altogether, these data suggest that SSE-7 activity is highly restricted to specific cell states and may be most active in a subset of fetal neural progenitors.

## SSE-7 activity in GBM core and margin

AAV is an excellent gene therapy vector for use in GBM owing to the following characteristics: (1) its small physical size (for enhanced distribution); (2) persistence in quiescent cancer cells; (3) low immunogenicity; (4) diverse natural capsids that might enable improved transduction; (5) validated efficient brain delivery via convection-enhanced delivery; and (6) well-understood manufacturing and clinical safety profile.

**Fig. 4:**
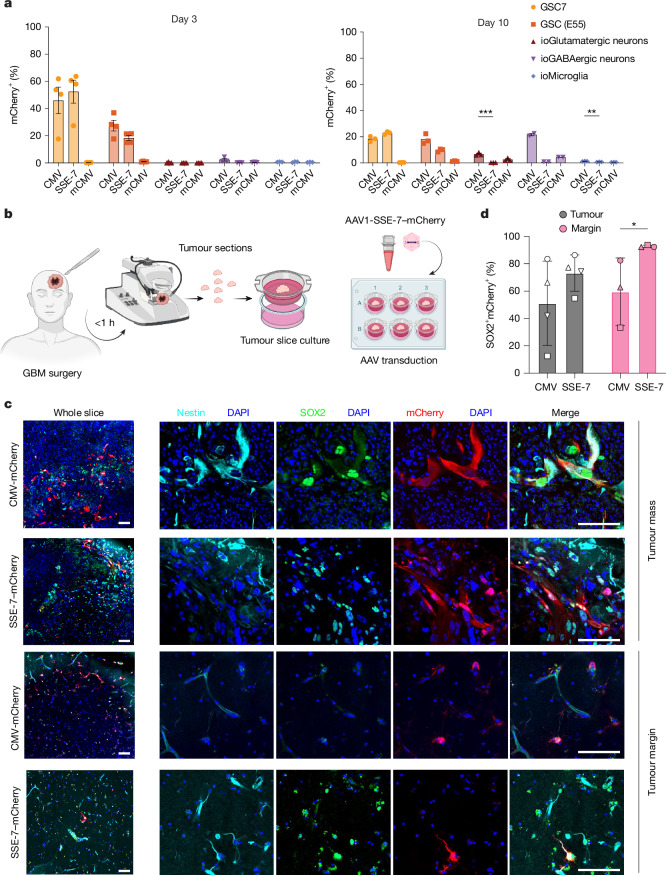
GSC selectivity of SSE-7 confirmed in vitro and in tissue slice cultures. **a**, AAV1-SSE-7–mCherry reporter activity in GSCs and human iPS-cell-derived differentiated neurons and microglia assessed by flow cytometry at day 3 (left) and day 10 (right) following transduction. For day 3 for ioGABAergic neurons and ioMicroglia, *n* = 3 biological independent cell experiments; ioGlutamatergic neurons and GSCs, *n* = 4 biological independent cell experiments. For day 10, *n* = 3 biological independent cell experiments excluding ioGABAergic neurons, for which *n* = 2. Data are the mean ± s.e.m., and statistical analysis was performed using an unpaired *t*-test. **P ≤ 0.01, ***P ≤ 0.001. **b**, Experimental schematic of AAV1-SSE-7 to test selectivity in fresh human GBM tumour mass and tumour margin tissue samples (infiltrating cortex) processed into slice cultures (Methods). **c**, Immunostaining and confocal images of tissue for mCherry (red) and GSC markers (Nestin and SOX2) demonstrates GSC selectivity of SSE-7 relative to CMV. Tumour mass and adjacent matched normal brain tissue (tumour margin) are shown. **d**, Percentage of mCherry-expressing cells co-expressing SOX2. Each point represents a biological replicate, matched tumour and tumour margin samples are indicated by matching symbols. *n* = 3 for margin and *n* = 4 for the tumour mass. Statistical analysis was performed using two-way ANOVA with multiple-comparisons correction (*P* = 0.0436). Scale bars, 10 µm. Schematic in **b** created in BioRender. Pollard, S. M. (2026) https://BioRender.com/l774d5t. Source data

To further explore SSE-7 selectivity using relevant adult human neural cell types and the effectiveness of AAV as the delivery vector, we generated human glutamatergic and GABAergic differentiated neurons (ioGlutamatergic and ioGABAergic, respectively), and microglia (ioMicroglia) from iPS cells, and tested AAV1-SSE-7–mCherry activity (Fig. 4a). SSE-7 was strongly expressed in GSCs within a few days of transduction, with similar levels to the CMV positive control. However, in postmitotic neurons, we did not observe SSE-7 activity (Fig. 4a) at either day 3 or day 10. For microglia, there was poor transduction with AAV1.

To directly test whether SSE-7 would have strong differential activity in GBM tumour tissue compared with the surrounding normal brain, we determined the transduction and expression of AAV1-SSE-7–mCherry in ex vivo fresh tumour tissue slices obtained from patients with GBM (Fig. 4b) and compared these with the tumour margin (which is macroscopically normal tissue but contains infiltrating GBM cells). We first tested whether AAV1-SSE-7 would be active in the tissue, focusing on the mCherry reporter only. After 7 days of incubation with a single dose of the virus, we fixed and stained the tumour tissue and compared CMV and SSE-7 activity using the mCherry reporter.

**Extended Data Fig. 9:**
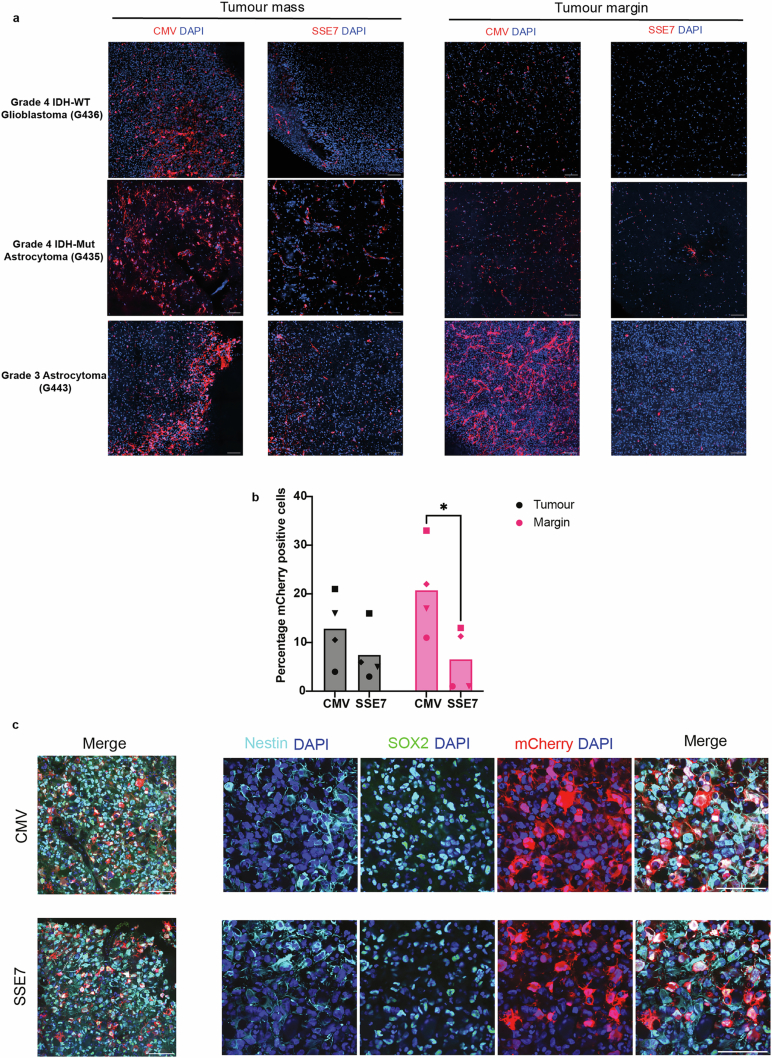
Selective activity of AAV1-SSE7-mCherry in SOX2 expressing cells within the tumour tissue mass and infiltrating margin. (a) Tissue slice-culture experiments were performed across diverse subtypes of high-grade glioma similarly to Fig. 2. Immunostaining and confocal for mCherry across distinct tumour subtypes shows restricted activity of SSE-7-mCherry in the tumour margin relative to CMV. (b) Quantitation of the % of mCherry expression in the tumour versus the margin shows SSE-7 is activated in a subset of GBM cells. (n = 4 for both tumour and margin with both CMV and SSE7). Matched tumour and margin samples were used across multiple tumour types; GBM IDH-WT (isocitrate dehydrogenase wild-type), Grade 4 Astrocytoma IDH-MUT (isocitrate dehydrogenase mutant), Grade 3 high-grade astrocytoma and denoted by distinct symbols, each point is a biological replicate. Error bars represent SD. Statistical significance was assessed by two-way ANOVA with multiple-comparisons correction (p = 0.0182). n = 4. Image analysis and segmentation pipelines provided in the Supplementary Data. Scale bar = 100 micron. (c) Staining for mCherry alongside SOX2 and Nestin in a recurrent GBM sample treated with the AAV1-SSE7-mCherry vector, confirming that SSE-7 is active in a subset of the tumour cells. Left panels, low magnification (no matched normal tissue was available for this recurrent sample). Source data

There were significantly fewer mCherry-positive cells when using SSE-7 compared with CMV (Fig. 4c and Extended Data Fig. 9b) in the tumour, and this was even more significant in the tumour margin tissue. This result is consistent with SSE-7 labelling a subset of GBM cells but not normal healthy tissue. We performed this analysis for four independent patient samples—grade 4 IDH mutant astrocytoma, IDH wild-type GBM, a grade 3 astrocytoma and recurrent GBM (Extended Data Fig. 9a)—and obtained similar results. By contrast, as expected, CMV induced widespread high expression across diverse normal CNS cell types, including cells with neuronal and endothelial morphology (Fig. 4c and Extended Data Fig. 9).

To next confirm whether mCherry is enriched in the tumour cell population, we performed and quantified SOX2 co-staining. This experiment revealed significant differences between CMV and SSE-7, with SSE-7 activated in around 90% of mCherry-positive tumour cells that co-expressed SOX2 (Fig. 4d). We conclude that SSE-7 transgene expression is differentially active in tumour versus normal tissue (tumour margin) compared with CMV. Moreover, SSE-7 provides a strong and selective gene switch that can restrict transgene activity to GSCs in the tumour mass and the infiltrating tumour margin.

## Complete responses in a mouse model of GBM

**Extended Data Fig. 10:**
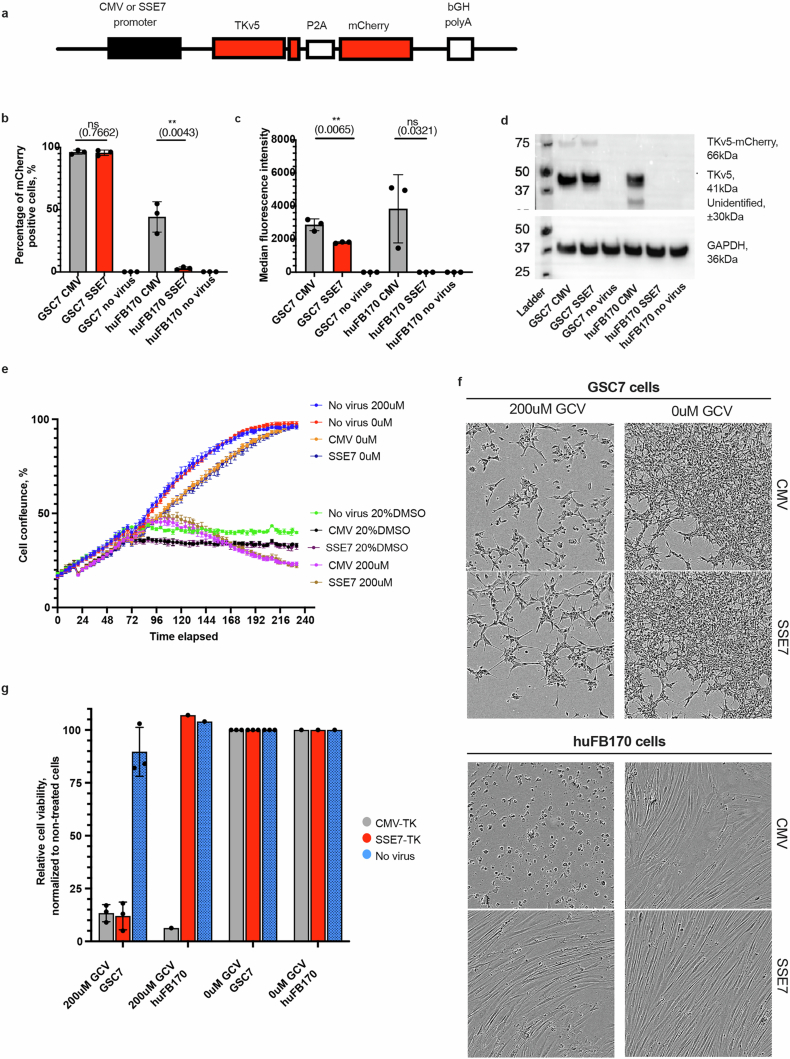
In vitro killing of GSCs, but not fibroblasts, is achieved using SSE7-driven HSV-TK. (a) Schematic of the AAV-2 transgene encoding both HSV-tk and mCherry used in vitro to assess selective cytotoxicity. (b and c) Flow cytometry was performed 4 days post-transduction. Each dot represents biological replicate (n = 3), error bars represent SD. (b) % mCherry positive cells and (c) median fluorescent intensity (MFI) of mCherry positive cells. (d) Western blot image developed against V5 and GAPDH in transduced and non-transduced GSC7 and huFB170 cells; the higher weaker band is the small fraction that was uncleaved. (e) Incucyte live cell imaging generate proliferation curves to determine the change in confluence over 10-day time-course for human GSC7 cells transduced with CMV or SSE-7-driven TKv5-P2A-mCherry in presence of 200 µM GCV, 0.2% DMSO (vehicle control) or 20% DMSO (positive control). (f; top panels) Live images of GSC7 cells at day 8 demonstrating cell number and morphological changes in cells transduced with constructs in the presence of 200 µM GCV. (f; bottom panels) Live images of huFB170 cells at day 17 demonstrating cell number and morphological changes in cells transduced with constructs in the presence of 200 µM GCV. (g) MTT assay for cell viability in GSC-7 and fibroblasts. Each dot represents an independent biological replicate. MTT values were normalized to vehicle-only control. Error bars represent SD (n = 3). Source data

**Extended Data Fig. 11:**
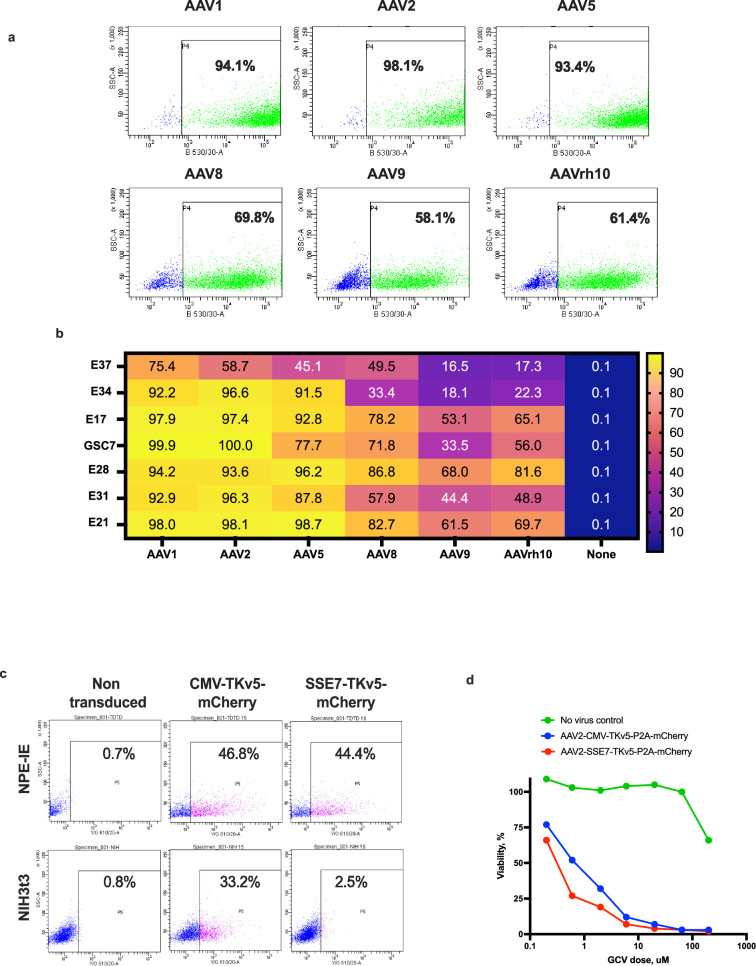
Evaluating SSE7-TKv5-P2A-mCherry activity from an AAV vector. (a) Example of flow cytometry data for GSCs (E17) cells following transduction at MOI of 5E5. (b) Heatmap summarizing mean AAV transduction efficiency in different patient-derived GSC cultures (n = 3, except E34 and E21, which are n = 2). (c) SSE-7 activity in mouse fibroblasts and GSCs. (c) Flow cytometry data comparing transcriptional activity between SSE-7- and CMV-driven TKv5-mCherry cassette in mouse NPE-IE cells (GSCs) and NIH3T3 cells when delivered using rAAV2. (d) MTT viability assay demonstrating GCV dose response kill curves in NPE-IE cells transduced with rAAV carrying specified constructs. Source data

We next determined whether AAV1-SSE-7 could be used to selectively kill GSCs using expression of the HSV-TK/GCV enzyme prodrug system, which has previously been applied in the clinic as a gene-directed enzyme prodrug therapy^29^. SSE-7 retained activity and provided selective expression of the HSV-TK payload in GSCs (Extended Data Fig. 10a–d). This enabled cytotoxic killing of GSCs, after GCV treatment, whereas fibroblasts were unaffected (Extended Data Fig. 10e–g). Screening of common natural AAV serotypes confirmed that AAV1 is the optimal serotype of choice, as it efficiently transduced all seven patient-derived GSCs with high efficiency (Extended Data Fig. 11a,b).

Despite the promise of HSV-TK as a suicide gene therapy approach for oncology, there has been limited success in the clinic. This is partially explained by the challenges of efficient delivery to all cancer cells^30^. As immune payloads can act non-autonomously to trigger an immune response against residual disease, we reasoned that combining an immune payload with a cytotoxic payload might be required to achieve this goal. Indeed, such a strategy could induce multiple pathways of the immune response to polarize local macrophages in the tumour microenvironment while enabling systemic adaptive responses by T cells. We proposed that this combination—tumour cell killing, tumour-associated macrophage polarization and T cell education and activation—would be potent and reduce chances of tumour regrowth from residual disease^30^.

**Fig. 5:**
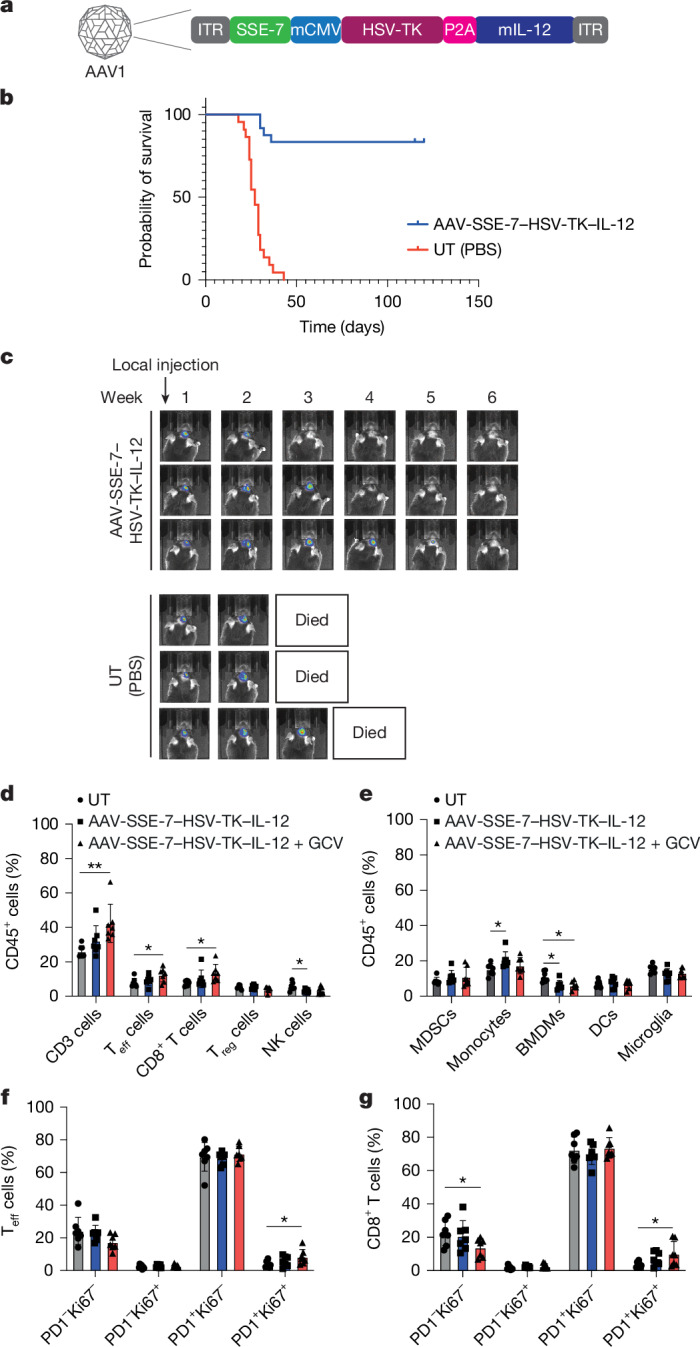
The combination of a cytotoxic and immunomodulatory payload is highly effective at clearing established GBM tumours. **a**, Architecture of the AAV1 transgene used to treat NPE-IE GBM in mice. ITR, inverted terminal repeat; mCMV, minimal CMV promoter; mIL-12, mouse IL-12. **b**, Survival curves for the mice treated with AAV1-SSE-7–HSV-TK–IL-12 (*n* = 24) versus control (UT (PBS); *n* = 22). *P* < 0.0001 for pooled (*n* = 3) independent cohorts. Survival was analysed using the Kaplan–Meier method. Significance was determined using a log-rank (Mantel–Cox) test. Mice were selected at week 1 using bioluminescence imaging to ensure a similar starting size for tumours across the cohorts. **c**, Longitudinal tracking of tumour growth in mice once a week using bioluminescence imaging (three examples shown for experimental and control; see Extended Data Fig. 12 for the larger cohort). **d**,**e**, Bar plots of the abundance of total T (CD3) cells, effector T (T_eff_) cells, CD8^+^ T cells, regulatory T (T_reg_) cells and natural killer (NK) cells (**d**), and myeloid-derived suppressor cells (MDSCs), monocytes, bone marrow-derived macrophages (BMDMs), dendritic cells (DCs) and microglia (**e**) out of total live CD45^+^ cells in the tumour microenvironment of untreated mice and mice treated with a single dose of AAV-SSE-7-HSV-TK–IL-12 or with the AAV-SSE-7-HSV-TK–IL-12 + GCV combination, determined by flow cytometry. **f**,**g**, Bar plots of the abundance of PD1^−^Ki67^−^, PD1^−^Ki67^+^, PD1^+^Ki67^−^ and PD1^+^Ki67^+^ cells from T_eff_ cells (**f**) and from CD8^+^ T cells (**g**) in the tumour microenvironment of untreated mice and mice treated with a single dose of AAV-SSE-7-HSV-TK–IL-12 or with the AAV-SSE-7-HSV-TK-IL-12 + GCV combination, determined by flow cytometry. *n* = 7 biologically independent animals per group, examined in one experiment. Data are the mean ± s.d. Significance was assessed using one-way ANOVA with Tukey’s multiple comparison test. *P ≤ 0.05, **P ≤ 0.01, ***P ≤ 0.001, ****P ≤ 0.0001. Source data

We chose the cytokine IL-12 for evaluation as it triggers a potent inflammatory response (stimulating adaptive and innate immunity) and is effective in promoting anticancer immune responses^31,32^. We reasoned that by using the AAV1-SSE-7 platform, we could avoid the toxicities previously seen when using systemic delivery^31^. This would enable controlled release of IL-12 alongside tumour killing and induce effective antitumour immune responses, with IL-12 levels being reduced as tumour cells are lost. We designed an AAV1 vector with SSE-7 driving the expression of a single mRNA encoding HSV-TK linked via the *P2A* sequence to the mouse IL-12 heterodimer fusion (*Il12a* and *Il12b*, which encode P40 and P35, respectively) (Fig. 5a). This dual payload design ensures that the cytokine and cytotoxic effects are synchronized and serves as a safety switch to prevent uncontrolled IL-12 levels.

For in vivo preclinical efficacy studies, we used our previously reported disease-relevant syngeneic mouse model of GBM, termed NPE-IE^25^, which exhibits a myelosuppressive tumour microenvironment and transcriptional programs seen in human GBMs^25^. AAV1-SSE-7 transduction and SSE activity were first validated using mouse NPE-IE cells in vitro (Extended Data Fig. 11c,d).

AAV1-SSE-7–HSV-TK–P2A-IL-12 was delivered to a cohort of mice with established orthotopic tumours (14 days after tumour cell orthotopic engraftment) using direct intratumoral injection with a microinjection pump to deliver a single dose of the virus. We observed substantial tumour regression within 1–2 weeks and complete clearance of tumours in 20–24 treated mice over the subsequent 2–3 weeks, whereas control mice died within 1–2 weeks (Fig. 5b,c).

**Extended Data Fig. 12:**
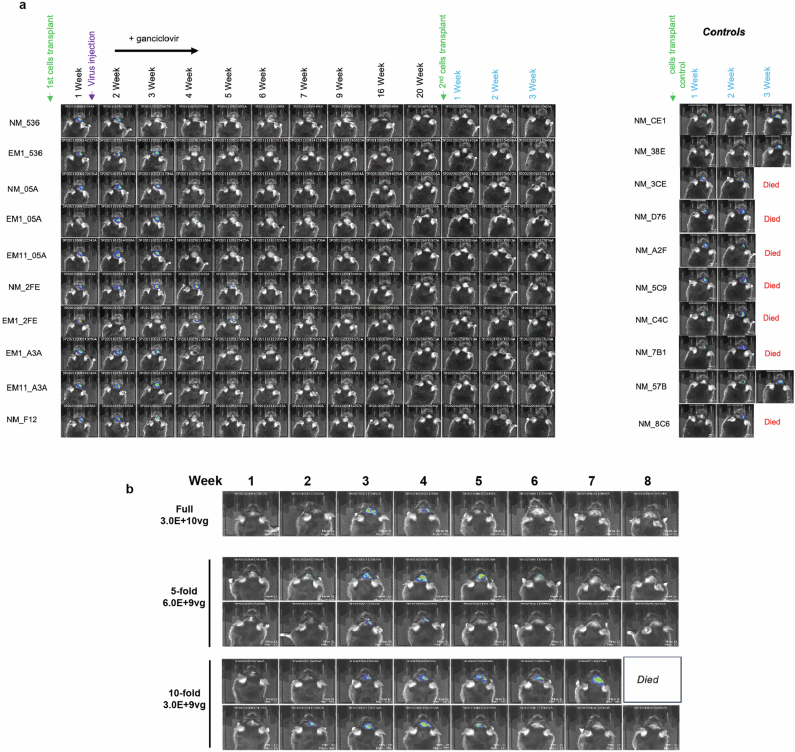
Treatment of mice with AAV-SSE7-TK-IL12 results in tumour clearance and long-term protection against rechallenge. (a) Bioluminescence imaging to track tumour growth with either control (PBS) injection (right panels) or virus plus 3-week ganciclovir treatment (shown are n = 10 mice from the first cohort of mice in Fig. 5b). Right panel are a cohort of parallel fresh controls using the same cells in new cohort of mice, confirming tumour initiation potential. Blue font indicates the new timeline after re-challenge with fresh tumour cells injected (male mice used). (b) Dose de-escalation using IVIS to track tumour growth following treatment with different doses of AAV1-SSE7-HSV-tk-IL12. (male mice used). Source data

Using bioluminescence imaging to track tumour burden in live mice, no further regrowth of tumours was observed after the initial tumours had cleared (Extended Data Fig. 12a). Moreover, no prominent toxicity, scored by weight loss and neurological monitoring, was seen over the subsequent 11 months (the experimental end point). We re-challenged treated mice (*n* = 10 that cleared the tumour) with fresh NPE-IE cell transplants into the striatum (5 months after initiation of the first therapy). Notably, there was no detectable tumour formation in any of the mice after the re-challenge, whereas parallel control mice formed tumours within 2–3 weeks (Extended Data Fig. 12a). Finally, antitumour activity was dose-dependent (Extended Data Fig. 12b).

## IL-12 with HSV-TK/GCV drives antitumour immunity

**Extended Data Fig. 13:**
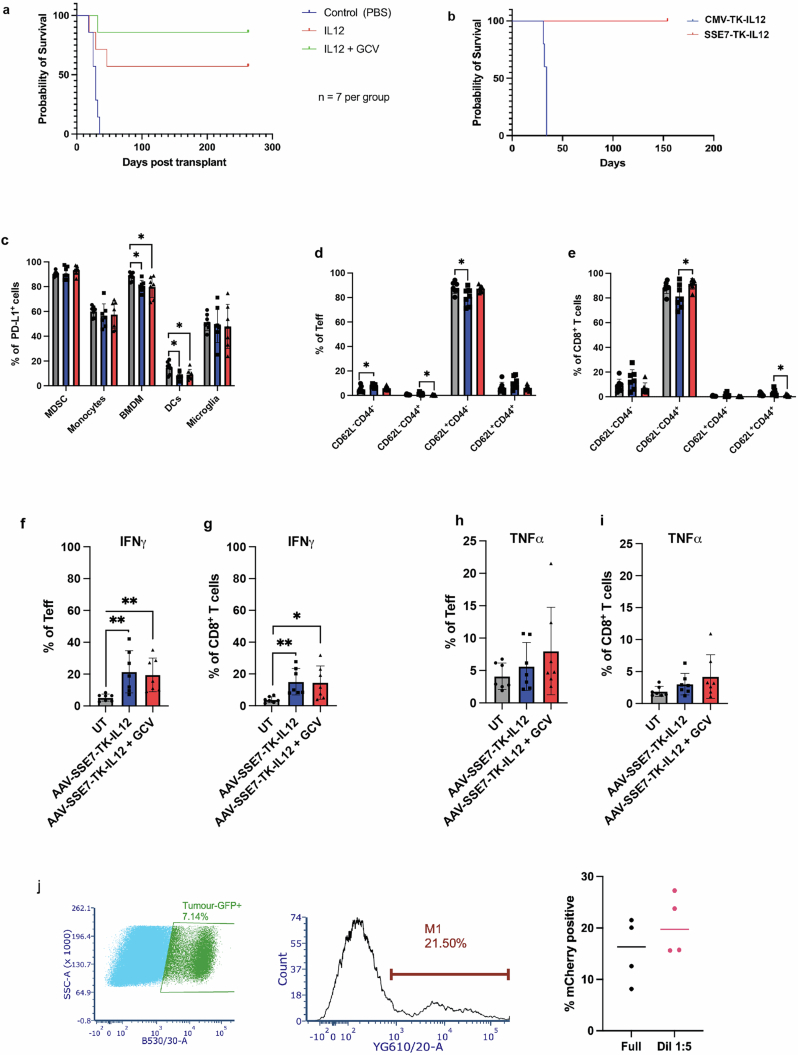
Characterisation of immune cells with single or double payloads. (a) Survival curves of tumour-bearing animals for either single (IL-12) or combination payloads (IL-12 plus TK/gcv), n = 7 male mice per group. Survival was analysed using Kaplan-Meier method. Statistical significance was determined using log-rank (Mantel-Cox) test with *p* = 0.0009. (b) Non-tumour bearing mice treated with AAV-SSE7-TK-IL12 versus the CMV variant. n = 5 male mice per group. Survival was analysed using Kaplan-Meier method. Statistical significance was determined using log-rank (Mantel-Cox) test with *p* = 0.0034. The CMV driven payloads are toxic. (c) Bar plots of the percentage of PD-L1+ cells in MDSCs, monocytes, BMDMs, DCs and microglia. (d, e) Bar plots of the percentage of CD62L-CD44-, CD62L-CD44+, CD62L+CD44- and CD62L+CD44+ cells in T effector cells or CD8+ T cells, respectively. (f,g) Bar plots of the percentage of IFNg producing cells in T effector cells. (h,i) Bar plots of the percentage of IFNg producing cells in CD8+ T cells. (j) Flow cytometry analysis of tumor mass 7 days following AAV1-SSE7-mCherry transduction, reveals that ~20% of the NPE-IE-GFP tumor cell population has strong mCherry expression (left panel, gating of tumor cells; middle panel, mCherry histogram; right panel, quantitation n = 4 independent tumours); 2ul of 3.6E14/ml (full dose) and 7.2E13/ml (1:5 dose). (c-i) n = 7 biologically independent animals per group, examined in one experiment. Data are represented as mean values +/− SD. Statistical significance was assessed using one-way ANOVA with Tukey’s multiple comparison test. *P ≤ 0.05; **P ≤ 0.01; ***P ≤ 0.001; ****P ≤ 0.0001. Source data

To determine whether IL-12 alone would be sufficient for eradicating tumours without the concomitant HSV-TK/GCV-induced killing (that is, single versus dual payloads, respectively), we treated mice with AAV1-SSE-7-HSV-TK–P2A-IL-12 alone or with this virus and with GCV. The dual payload was the most effective and enhanced the effects of IL-12 alone (Extended Data Fig. 13a). To explore safety in normal CNS, we also assessed responses in mice without tumours to either CMV-driven or SSE-7-driven HSV-TK–IL12. As expected for CMV, there were rapid neurological issues within weeks, consistent with the expected toxicities with continuous expression of IL-12 in normal CNS. By contrast, we observed no toxicity with SSE-7, a result consistent with our earlier in vitro data confirming the selectivity of this SSE (Extended Data Fig. 13b).

Characterization of the immune cell composition after treatment using either IL-12 alone or IL-12 with HSV-TK/GCV revealed that the latter performed better, with significantly increased levels of IFNγ and a trend towards increased TNF (Extended Data Fig. 13c–i). This finding is consistent with the observed increase in cytotoxic T cells and reduction in myelosuppressive cells that occurs through T helper 1-like responses induced by IL-12 (Fig. 5d–g).

There was also an increase in proliferative Ki67^+^ T cells, with a shift towards increased CD44^+^, a marker of T cell activation and memory (Extended Data Fig. 13d,e). To assess the percentage of transduction in the tumour mass, we used the SSE-7–mCherry reporter. Tumours were collected, dissociated and assessed using flow cytometry, which revealed around 20% positive tumour cells (GFP^+^mCherry^+^) (Extended Data Fig. 13j). This transduction efficiency was achieved using the same viral doses as the survival analysis experiments. This result confirms that only a fraction of the tumour mass needs to be transduced to achieve immune clearance of residual disease.

In conclusion, AAV1-SSE-7–HSV-TK–IL-12 is highly effective at eliminating tumours in an aggressive syngeneic mouse GBM model. Treated mice were resistant to subsequent re-challenge with fresh NPE-IE cells. A single dose of AAV1-SSE-7–HSV-TK–IL-12 induced complete responses with durable immune memory without off-target toxicity.

## Discussion

The development of effective gene therapies for oncology will require more effective payload combinations and more selective delivery mechanisms than has been possible so far^30,33,34^. We showed that SSEs, combined with delivery via AAV1, can address one of the significant challenges in oncology: namely, how to specifically deliver combinations of anticancer payloads at high doses without off-target toxicity. Across genetically diverse GSC lines and high-grade glioma tissues, SSE-7 consistently read out a core transcriptional program characteristic of GSC identity: the immature NSC-like state.

Our findings indicated that SOX2 and SOX9 share crucial target genes and may represent a core transcriptional module, an ‘oncofetal’ program, that defines aggressive tumour-initiating cells in GBM^18,35^. Using AAV-mediated delivery and SSE control of a transgene, we achieved specific elimination of active GSCs by targeting them from within, akin to a Trojan horse approach. The persistence of AAV vectors, and our focus on the shared SOX circuits, meant that we were able to guard against plasticity, as transgene expression will be reactivated if cells transit through this specific cell state.

Immunotherapy approaches have generally had limited success in GBM^36–38^. Our AAV–SSE approach will now require careful safety studies to determine the potential for translation to patients. The goal is to deliver cytotoxic payloads alongside innate and adaptive immune activation to achieve meaningful and durable suppression of high-grade gliomas without significant off-target toxicities.

Future studies will explore the molecular mechanisms that underlie SSE activity, which may involve transcriptional hubs or condensates. Our findings from exploring the biochemical binding of SOX2 and SOX9 suggests that there is clear synergy between these two factors. However, additional cofactors are required for the creation of a multimeric complex. Detailed structural studies are needed to precisely determine how SOX2 and SOX9 operate and the signalling TF requirements and whether nucleosome context is critical^39^. In the future, improved computational methods and artificial-intelligence-based tools for the design of enhancers and synthetic associated motifs will complement the functional screening approaches used here^40–42^. In conclusion, our study demonstrated the potential of SSEs and AAV vectors to deliver precision immune activation for the treatment of cancer.

## Methods

### Destination vector cloning

We built a specific custom destination vector for efficient Golden Gate cloning of enhancer fragments. This included the reporter gene cassette NanoLuc-Ires-mNGreen-pA, downstream of a mCMV promoter. A bacterial suicide ccdB cassette spanning the enhancer position enabled assembly of a single enhancer for selection and efficient cloning. This was based on the EMMA Golden Gate cloning system^43^. For the GSC long-term differentiation experiments, the same vector was built but included PiggyBac transposase recognition sites flanking the entire cassette.

### Bioinformatics to design a SOX2 enhancer oligonucleotide pool

We re-analysed previously published^1^ SOX2 ChIP–seq and H3K27ac GSC cell line data to identify GSC-specific SOX2 peaks that were overlapping with H3K27ac and absent in differentiated cells (cells in serum culture). The resulting shared peaks were then combined into one set and manually curated to remove centromeres. This resulted in 1,721 peaks with an average length of 402 bp. These peaks were then used to design a set of 160 bp sequences. Next, 20 bp adapters were included to flank the sequences and these sequences were synthesized as an oligonucleotide pool (Twist Bio).

### Construction of an arrayed plasmid library of enhancer fragments

The oligonucleotide pool (9,523 unique sequences) was first amplified for 10 cycles with 0.25 µl (2.5 ng input) volume, and 0.5 µl of this reaction was used for the subsequent 15 cycles of amplification to reduce PCR ‘jackpot’ amplification. The final products were cloned into the expression vector using an efficient Golden Gate reaction. We used KAPA HiFi Hotstart polymerase with GC buffer (Roche, KK2501), 68 °C annealing temperature and 5 s of extension time at 72 °C. A total of 4,579 individual plasmids were then randomly picked, miniprepped and plated as an arrayed plasmid DNA library on 96-well plates. This was deemed a practical number for arrayed library screening. A magnetic-bead-based SPRI purification method was used to minimize loss and to clean up the DNA before Golden Gate cloning. Sanger sequencing for quality checks of a sample of the plasmids confirmed that they were diverse, and all sequences could be mapped back to the original library design.

### Screening platform for 384-well plates

Cells were seeded in 384-well plates using a multidrop and transfected the following day using CyBio Felix (CyBio). Two days later, a NanoGlo DLR assay was used to identify hits with a fold change to mCMV of >10. These hits were Sanger sequenced to determine the specific sequence and mapped back to the genome to validate overlap with the original SOX2 ChIP–seq data. The GREAT tool predicted the target gene for hit sequences^44^.

### General cell culture procedures

The GSC lines GCGR-E17 (E17), GCGR-E21 (E21), GCGR-E27 (E27), GCGR-E28 (E28), GCGR-E31 (E31), GCGR-E34 (E34), GCGR-E37 (E37) and GCGR-E55 (E55) and the human NSC lines NS9FB_B (NS9), NS12ST_A (NS12) and NS17ST_A (NS17) were generated in the Pollard Laboratory and are available upon request from the Glioma Cellular Genetics Resource. Informed consent was obtained for use of patient tissue. All procedures on patient brain tissue received ethics approval from the NHS Health Research Authority, East of Scotland Research Ethics Service (REC reference 15/ES/0094), and all procedures on embryonic and fetal brain tissue received ethics approval from the Lothian NHS Board, South East Scotland Research Ethics Committee (REC reference 08/S1101/1).

Cells were grown as adherent monolayers under serum-free conditions as previously described^13^. All cells lines tested negative for mycoplasma at passage 3 using a MycoAlert Mycoplasma Detection kit (Lonza, LT07-318). Cell line authentication by STR profiling was performed as a service by the European Collection of Authenticated Cell Cultures (ECACC). Reports are available upon request from the Glioma Cellular Genetics Resource. Analysis was conducted using the Promega Fusion system (DC2402), analysing differences at 24 distinct hypervariable genetic loci, 16 of which are used for the final STR profile and report. The ioGlutamatergic (io1001S) and ioGABAergic (ioEA1003S) neurons and ioMicroglia (ioA021) were obtained from bit.bio and grown per the manufacturer’s specifications. GSC7 was previously characterized^22^.

For lipofection, Plus reagent and Lipofectamine LTX (Life Technologies, 15338030) were each diluted in half the volume of Opti-MEM I reduced-serum medium (hereafter referred to as Optimem) (Life Technologies, 31985062). Following this step, the Plus reagent and Optimem premix was added to all DNA samples followed by the Lipofectamine LTX and Optimem premix. The transfection mix was incubated for 5 min at room temperature and then carefully dropped onto the cells. Generally, no change in medium was carried out. Cells were analysed 2 days after transfection.

HEK293 cells were seeded at the specified density in the respective plate format. The next day GMEM medium, DNA and polyethylenimine were mixed and incubated for 15 min at room temperature to facilitate formation of the complex. The transfection mix was subsequently dropped onto the cells, and analysis was carried out 2 days later.

### Screening assay using the Nano-Glo Dual-Luciferase reporter assay system

This assay consists of two steps. Transfection was performed using a normalization plasmid PGK-Firefly-Luciferase (Promega, E5011) transfected in a 1:10 ratio with the plasmid of interest. Therefore, in this assay, the firefly luciferase activity is first measured, which enables normalizing of the transfection efficiency. Cells were washed 3 times with PBS and 20 µl was left in a 96-well plate (25 µl in a 384-well plate). Oneglo buffer was added, and the plate was shaken for 5 min at 480 rpm to allow cell lysis. Next, 20 µl cell lysate in a 96-well format (25 µl cell lysate in a 384-well format) was transferred into an opaque white 96-well format (or 384-well format) and light was measured for 0.1 s per well (Ensight Multimode Plate Reader, Perkin Elmer). For the subsequent NanoLuc reaction in a 96-well format, 2 µl cell lysate was transferred into an opaque white plate containing 40 µl PBS, and 20 µl Stopglo buffer, supplemented with substrate, was added to each well. In a 384-well format, 20 µl Stopglo buffer containing substrate was added on top of the undiluted cell lysate. The plate was shaken again for 5 min at 480 rpm to quench the firefly luciferase reaction and to ensure good mixing. NanoLuc activity was measured for 0.1 s per well. Data obtained by the NanoLuc reaction were normalized to the Firefly reaction to account for well-to-well variability of transfection efficiency. Normalized data were used to calculate the fold change compared with the empty vector control, which contained mCMV only and no enhancer fragments.

### Immunocytochemistry

Cells were washed twice with PBS and fixed in 4% paraformaldehyde (PFA) for 10 min. Cells were permeabilized with 0.1% Triton-100 in PBS (PBST). Cells were blocked with blocking solution (1% BSA in PBST with 3% goat serum) and incubated with the primary antibody at 4 °C overnight. The next day, cells were washed 3  times in PBST, and the respective secondary antibody was applied in blocking solution and incubated for 45–60 min at room temperature. Cells were washed with PBS and incubated for 5 min with a DAPI nuclear counterstain at 1 µg ml^–1^ final concentration. Images were acquired using a Nikon TiE microscope and NIS elements software (Nikon).

### Flow cytometry

For flow cytometry, cells were detached, pelleted and resuspended in an appropriate volume of flow cytometry buffer (1% BSA in PBS, v/v) or PBS. Cells were stained with Draq7 (Abcam, ab109202, final concentration of 0.1 µM) or DAPI (ThermoFisher Scientific, D3571) as a live/dead stain and analysed using a BD LSRFortessa cell analyser (4 lasers, BD Bioscience). Analysis of flow cytometry data was carried out using FlowJo Analysis software (v.10.6.2) or FCS Express 7 flow cytometry software (v.7.22.x).

### RNA isolation, cDNA synthesis and RT–qPCR

RNA was extracted using a MasterPure RNA Purification kit (Epicentre). RNA was stored at −80 °C, and concentration was determined using a NanoDrop spectrophotometer. cDNA synthesis was carried out using a SuperScript III Reverse Transcriptase kit (Life Technologies) according to the manufacturer’s instructions. Around 200–500 ng RNA was used for the cDNA reaction. The same amount of RNA was used for each experiment. After the reaction, cDNA was diluted to the required volume using nuclease-free water.

For RT–qPCR, TaqMan Universal PCR master mix (Applied Biosystems) and TaqMan gene expression assays (Life Technologies) were used on a Quant Studio7 Flex Real-Time qPCR machine. RNA samples that did not undergo reverse transcription (to assess DNA contamination) and water controls were used on every plate. RT–qPCR was carried out in technical duplicates. Data analysis was performed using the dd*C*_t_ method, which assumes 100% PCR efficiency and is guaranteed with TaqMan assays. In brief, the mean was calculated for technical replicates and normalized to the housekeeping gene *GAPDH*, which produces the d*C*_t_ value.

### Western immunoblotting

Cell lysates were prepared by resuspending cells in RIPA buffer (50 mM HEPES pH 7.7, 150 mM NaCl, 1% NP-40, 0.5% DOC and 0.1% SDS), incubating on ice for 5 min, centrifuging at maximum speed for 10 min in a tabletop centrifuge (5415D, Eppendorf) and collecting the supernatant (lysate). Protein extracts were quantified using a Pierce BCA Protein Assay kit (Thermo Scientific, 23225). SDS–PAGE was performed using homemade 4–12% Bis-Tris gels. Gels were transferred onto PVDF membranes (Millipore, IPVH00010), previously activated in methanol, by wet electroblotting or semi-dry blotting using a Bio-Rad Trans-blot turbo system. Western blots were revealed using HRP-conjugated antibodies, homemade ECL solutions or Clarity Western ECL (Bio-Rad), and imaged using X-ray films or a Bio-Rad ChemiDoc Imaging system.

### Precipitation of interacting proteins

PCR amplification with biotinylated primers and purification were used to generate templates that could pull down bound proteins. Enhancer fragments were amplified with 5′ biotinylated primers using PrimeStar Max (Takara) according to the manufacturer’s instructions.The following primers were used: forward primer 595: TGATCCGTCTC**G**CCCT**ACTAGGTTACTGGTGCATGC**; reverse primer 596: ACTAACGTCTC**G**GAGC**ACCCAAACTATTGGAGCGAG** (bold sequences, adaptors).

PCR purification using Agencourt AMPure XP magnetic beads (Beckman Coulter) was carried out according to the manufacturer’s instructions. PCR product quantification using Tapestation and reagents was carried out according to the manufacturer’s instructions.

All the buffers were prepared the day before, passed through a 22 µm filter (except the dialysis buffer) and left at 4 °C overnight. DTT and protease inhibitors were added immediately before use. Cell pellets were resuspended in 5 ml per 40 million cells of ice-cold buffer A (10 mM HEPES pH 7.9, 1.5 mM MgCl_2_, 10 mM KCl, 0.5 mM DTT and protease inhibitors (complete, Roche, 11697498001)) and incubated on ice for 10 min. The cell suspension was transferred to a glass Dounce homogenizer and dounced 40 times on ice, transferred to a Falcon tube and centrifuged at 1,350 rcf for 10 min at 4 °C. The supernatant was discarded (cytosolic extract) and the pellet was resuspended in 100 µl per 10 million cells in ice-cold buffer B (20 mM HEPES pH 7.9, 5% glycerol, 1 M NaCl, 1.5 mM MgCl_2_, 0.2 mM EDTA pH 8.0, 0.5 mM DTT and protease inhibitors (complete, Roche, 11697498001)). The suspension was rotated for 30 min at 4 °C and transferred into a dialysis membrane (SnakeSkin, Thermo Scientific, 68100). Dialysis was carried out in 500 ml dialysis buffer (20 mM HEPES pH 7.9, 5% glycerol, 100 mM KCl, 0.83 mM EDTA pH 8.0, 1.66 mM DTT and protease inhibitors (complete, Roche, 11697498001)) for 2 h at 4 °C with rotation. The dialysis buffer was changed (500 ml), and dialysis was continued overnight at 4 °C. The extract was collected in a 1.5 ml tube and spun at maximum speed for 15 min at 4 °C (centrifuge 5415D Eppendorf). The supernatant was collected into new 1.5 ml tubes and quantified (Pierce BCA Protein Assay kit, Thermo Scientific, 23225). Next, 100 µg aliquots were flash-frozen in liquid nitrogen and stored at −80 °C. The following primary antibodies were used for western blotting: anti-SOX2 (rabbit) 1:1,000 Abcam (ab92494); anti-SOX9 (rabbit) 1:500 Millipore (ab5535); and anti-GAPDH (mouse) 1:10,000 Ambion (AM4300). The secondary antibodies used for western blotting were anti-rabbit 1:5,000 Novex (A16110) and anti-mouse 1:5,000 Novex (A16027).

Streptavidin magnetic beads (NEB, S1420S) were used according to the manufacturer’s instructions. In brief, 10 µl beads were aliquoted into low-bind tubes and washed 3 times with binding buffer (20 mM Tris-HCl pH 7.5, 0.5 M NaCl and 1 mM EDTA) on a magnetic stand. Next, 20 µl biotinylated DNA (around 20 pmol) was mixed with 200 µl binding buffer and was added to the beads. The suspension was rotated at room temperature for 2 h. The beads were washed 3 times with binding buffer, and 50 µg nuclear extract was added (total volume of 50 µl). Beads, DNA and nuclear extract, to which 150 µl dialysis buffer (20 mM HEPES pH 7.9, 5% glycerol, 100 mM KCl, 0.83 mM EDTA pH 8.0, 1.66 mM DTT and protease inhibitors (complete, Roche, 11697498001)) was added, were rotated overnight at 4 °C. The following morning, beads were washed 3 times with wash buffer (20 mM HEPES pH 7.9, 5% glycerol, 250 mM NaCl, 0.83 mM EDTA pH 8.0 and 1.66 mM DTT). For western blotting, proteins were eluted in 20 µl loading buffer (lithium dodecyl sulfate buffer containing 50 mM DTT) and boiled for 5 min to denature the proteins. For mass spectrometry, the beads were sent dry to the Institute for Genetics and Cancer core facility (University of Edinburgh) for analyses.

### SOX2 and SOX9 ChIP–seq library preparation and analysis

In brief, GSCs cultured in 150 mm dishes to 70% confluence were crosslinked in 1% formaldehyde for 10 min at room temperature. Excess formaldehyde was quenched by incubating cells with 0.25 M glycine for 5 min at room temperature. The cell pellet was washed twice with ice-cold PBS before storage at −80 °C. For each GSC line, cell pellets from eight 150 mm dishes were combined before proceeding for cell lysis and sonication. The cell pellet was lysed in buffer LB3 (10 mM Tris-HCl pH 8.0, 100 mM NaCl, 1 mM EDTA pH 8.0, 0.5 mM EGTA pH 8.0, 0.1% sodium deoxycholate, 0.5% *N*-lauroylsarcosine and 0.1% SDS) and chromatin was fragmented to 200–600 bp size on a Covaris M220 sonicator (total of 4 cycles, with the following treatment settings: 600 s per cycle, peak power 75, duty factor 10, cycles/burst 200; temperature: minimum 5 °C, set point 7 °C, maximum 9 °C). For each ChIP assay, 15–20 µg fragmented chromatin was incubated overnight in a cold room with 10 μg antibodies (R&D SOX2 AF2018, Millipore, SOX9 AB5535) and 30 μl Protein G Dynabeads (10003D). The next day, magnetic beads were washed 5 times with RIPA (50 mM HEPES-KOH pH 7.5, 0.5 M LiCl, 1 mM EDTA pH 8.0, 1% NP-40 and 0.7% sodium deoxycholate), once with TE NaCl (10 mM Tris-HCl pH 8.0, 1 mM EDTA pH 8.0 and 50 mM NaCl) and finally eluted in 200 μl ChIP elution buffer (50 mM Tris-HCl pH 8.0, 10 mM EDTA pH 8.0 and 1% SDS). Immunoprecipitated chromatin was reverse crosslinked by incubating samples at 65 °C for 10 h and extracted using the phenol–chloroform–IAA (11896714) method. To account for intertumoral heterogeneity, ChIP–seq was performed on seven independent primary GSC lines. Libraries were prepared for two technical ChIP replicates and one input control using a NEBNext Ultra II DNA Library Prep kit for Illumina (E7645S). The libraries were barcoded using NEBNext Multiplex Oligos for Illumina (Dual Index Primers Set 1 kit, E7600S) and sequenced on an Illumina Novaseq (50 bp read length).

Paired-end reads were aligned to the hg38 genome using BWA, filtering out poor-quality (MAPQ < 10), duplicates, mitochondrial genome and blacklisted regions (https://www.encodeproject.org/annotations/ENCSR636HFF/). Technical replicates were merged, and peaks were called using MACS2 with default settings. Consensus SOX2 and SOX9 peak sets were derived by taking the overlap of peaks occurring in 5 out of 7 GSCs for each TF set. The overlap significance between the consensus SOX2 and SOX9 peak sets was determined using a circular permutation test in regioneR (*n*_times_ = 10,000). Genomic regions near SOX2 and SOX9 peaks were annotated using HOMER^45^.

To perform overlapping analysis with GSC super-enhancers, we downloaded raw fastq files from publicly available H3K27ac datasets on GSCs (GSE119834, GSE74529, GSE121601 and GSE92458) and called super-enhancers using ROSE with default settings. A consensus set of GSC super-enhancers was derived by considering super-enhancers that occurred in at least two GSCs. The overlap significance between the consensus co-bound SOX2–SOX9 sites and consensus GSC super-enhancers was performed using a circular permutation test as described above. Gene ontology term association analysis was performed using GREAT. To find centrally enriched de novo motifs at SOX2 and SOX9 peaks in co-bound SOX2–SOX9 enhancers, and to identify the spacing between the most significant de novo motifs from each peak set, we used CentriMo and SpaMo from the MEME-ChIP suite of tools^46^.

### scRNA-seq sample preparation

On the day of cell seeding, cells were detached as described above. Cells were counted using a haemocytometer and seeded into a 6-well Corning plate at 400,000 cells per well in 2 ml. Plates were incubated at 37 °C with 5% CO_2_. The next day, AAV vector stocks were thawed at room temperature. The appropriate amount of virus stock was added to the culture medium to achieve a final multiplicity of infection (MOI) of 1,000,000, which ensured high transduction efficiency. Viral-containing medium was added to cells without replacing the existing medium. The cells were then returned to the incubator at 37 °C with 5% CO_2_.

After 3 days of incubation with the viral particles, cells were detached as described above. Next, 10% of the cells were prepared for flow cytometry using DAPI as a live/dead stain (see Supplementary Information for the gating strategy). The remaining 90% of the cells were processed for single-cell transcriptome sequencing.

Cells were fixed using a Parse Evercode v.2 Cell Fixation kit following the manufacturer’s instructions, with an average of around 400,000 cells per sample. Fresh reagents were prepared for each fixation, and 7.5% Gibco BSA fraction V was added per the manufacturer’s recommendations. In brief, cells were washed with PBS and resuspended in cold Cell Prefixation buffer. Cells were filtered through a 40 µm strainer after 5 min of centrifugation at 300*g* at 4 °C. Following this step, Cell Fixation solution and Cell Permeabilization solution were subsequently added, with incubation on ice for 10 min and 3 min, respectively. Cell Neutralization buffer was then added, and cells were centrifuged again at 300*g* for 5 min at 4 °C. Finally, cells were resuspended in a volume of 50–90 µl Cell Buffer containing 1% DMSO based on the live cell number before fixation. Cells were filtered through a 40 µm strainer and stored at −80 °C for up to 3 months.

### scRNA-seq library preparation

Libraries were generated using a Parse Evercode WT Mega v.2 kit according to the manufacturer’s instructions. In brief, 72 samples were loaded into a 96-well RT barcoding plate for reverse transcription, with each well containing a unique barcoded RT primer. Following cDNA synthesis, cells were pooled and subjected to two additional rounds of barcoding, achieving three rounds of barcoding. Barcoded cDNA was then amplified via template switching and pooled into 15 sublibraries, each containing around 62,500 cells. A fourth barcode was incorporated during PCR amplification of the sequencing libraries. Sublibraries were then fragmented, ligated with Illumina adapters and purified using Ampure XP beads. Library quality was assessed on an Agilent BioAnalyzer 2100 before sequencing. Sublibraries, with 5% PhiX spike-in, were sequenced on an Illumina NovaSeq X using 300 cycles kits as paired-end, dual-index reads.

### scRNA-seq analysis

Sequencing reads were aligned to the human (hg38) genome, and UMI counting data were generated following the standard Parse Bioscience pipeline, ‘splitpipe (v.1.1.2)’, with default parameters. Three custom genes were added to the genome (mCherry, HSV-TK and bGHployA) to capture ‘activated’ SSE-7 cells. In total, 345,495 cells were included in the initial dataset. Low-quality cells were filtered out based on the following thresholds: (1) the percentage of mitochondrial gene was >20%; (2) the number of genes was <300; (3) the number of uniquely aligned reads was <500; or (4) the number of uniquely aligned reads was >20,000. Genes detected in fewer than five cells were filtered out as low-quality genes. Potential doublets were identified using the R package ‘DoubletFinder’ (v.2.0.4), with an expected doublets rate of 3%, as guided by Parse. Doublets and clusters containing more than 20% doublets were removed. Ambient RNA-contaminated cells (cell score > 0.2) were removed using ‘decontX’ from the R package ‘celda’ (v.1.18.2). After filtration, 270,842 cells remained for further analysis.

The data were normalized using ‘LogNormalize’, and principal component analysis was performed based on the top 2,000 variant genes using the R package ‘Seurat’ (v.5.0.3). The first 15 principal components were used as input for Louvain-based graphing. SSE-7-activated cells (2,711 cells) were labelled according to the following criteria: mCherry > 1 or HSV-TK > 1 or bGHpolyA > 0. Cells with a single count of mCherry or HSV-TK from the virus-treated group were labelled as false negative (9,583 cells), whereas the remaining cells were labelled as SSE-7 non-activated cells (125,525 cells). Meanwhile, cells expressing mCherry or HSV-TK in the control group were labelled as false positive (1,998 cells). Transcriptional subtypes were predicted on the basis of a previously described gene signature^27^ using the function ‘sigScores’ and ‘as_four_state_gbm’ from the R package ‘scalop’ (v.1.1.0). Cell cycle was predicted using ‘CellCycleScoring’ from the R package ‘Seurat’.

### SCENIC analysis of scRNA-seq data

False-negative and false-positive cells were removed, and the dataset was divided into 15 libraries as per the sequencing library preparation. The dataset was then analysed following the standard ‘pyscenic (v.0.12.1)’ pipeline. Auxiliary human datasets (hg38 mc_v10_clust) were downloaded from the cisTarget resources website (https://resources.aertslab.org/cistarget/). Motif–TF annotation was based on 10 kbp upstream and downstream around the transcription start sit (20 kbp in total). SSE-7-enriched TF modules were selected on the basis of the following criteria: (1) *Z* score > 0.7 and (2) *Z* score in the SSE-7-activated group was higher than in other groups, as determined by repeated-measures ANOVA test. Enrichment for candidate TF modules across 15 libraries was as follows: SOX8 (found in 9 libraries), STAT1 (found in 15 libraries), IRF9 (found in 12 libraries), ETS1 (found in 13 libraries), MAF (found in 3 libraries), SMAD1 (found in 12 libraries), FOS (found in 11 libraries), HIF1A (found in 7 libraries), SOX9 (found in 10 libraries), STAT3 (found in 3 libraries), NEF2L2 (found in 13 libraries), JUN (found in 2 libraries) and SOX2 (found in 2 libraries).

### Lentivirus production and titration

To produce SOX2, SOX9 and rtTA2M2 individual lentiviral supernatant samples, nearly 2.4 million HEK293T cells per 15 cm plate were cultured for 24 h in GMEM medium supplemented with 10% fetal calf serum (FCS), 1 mM sodium pyruvate, 1 mM glutamine and non-essential amino acids (HEK medium) at 37 °C and 5% CO_2_. At 24 h after seeding, HEK293T cells were transfected with a plasmid cocktail containing 7.5 μg expression plasmid, 5.1 μg psPAX2 packaging vector, 2.4 μg envelope vector mixed in 45 μl Fugene6 (Roche) and 855 μl Opti-MEM medium (Invitrogen, Thermo Fisher Scientific). The cells were further cultured for 16 h at 37 °C and 5% CO_2_ before the medium was changed for HEK medium. At 65 h after transfection, virus-containing supernatant was collected and cleared by centrifugation and filtered through a 0.45 μm syringe filter (Merck Millipore). Virus was concentrated (roughly 100-fold) by transferring to thin-walled ultracentrifuge tubes and pelleted by ultracentrifugation at 25,000 rpm for 2.5 h using a SW32-TI rotor in a Beckman Optima XPN ultracentrifuge (Beckman Coulter). The pelleted virus was resuspended in 300 μl plain GMEM medium and incubated at 4 °C for 16 h before aliquoting and flash-freezing in liquid nitrogen for storage at −80 °C. The viral titre, calculated by flow cytometry, was 6 × 10^7^ infectious units per ml.

### Overexpression of SOX2 and SOX9

Early passage (passage 4) human fibroblasts (hFibs) were seeded, 1.5 million per 15 cm plate, and cultured in hFib medium (GMEM medium supplemented with 10% FCS, 1 mM sodium pyruvate, 1 mM glutamine, MEM 1× non-essential amino acids solution, 50 μM 2-mercaptoethanol (Gibco, Thermo Fisher Scientific) and 2 ml penicillin–streptomycin (Invitrogen, 15140122)) 24 h before transduction at 37 °C and 5% CO_2_. The cells were transduced by exchanging the growth culture medium with lentiviral medium, without antibiotics, containing 8 μg ml^–1^ polybrene, and 65 μl concentrated rtTA2M2 and either SOX2 or SOX9 virus, for single factor overexpression, or 75 μl rtTA2M2, SOX2 and SOX9 viruses for double factor overexpression. At 48 h after transduction, the cells were split 1:2 and cultured for 24 h, after which the culture medium was replaced with induction medium containing 1 μg ml^–1^ doxycycline (induction day 0). After 48 h of induction, the cells were collected for lysis and nuclear protein extraction.

### Nuclear extraction of GSC7 and hFib cells

GSC7 cells were cultured as adherent monolayers^13^. For differentiation, cells were cultured without the supplements EGF and FGF and in 5% serum for 2 weeks. GSC7 and hFib nuclear lysates were prepared in the same way; cells were resuspended to 1 million cells per 100 μl 10 mM HEPES pH 7–8, 1.5 mM MgCl_2_, 10 mM KCl, 0.5 mM DTT, supplemented with complete Ultra EDTA-free protease inhibitors (Roche, 05892970001) and homogenized using a dounce and tight-fit pestle with 1 stroke per 1 million cells. Samples were centrifuged at 1,350 rcf for 5 min at 4 °C. The nucleus pellet was resuspended to 10 μl per 1 million starting cells in 20 mM HEPES pH 7–8, 30% glycerol, 420 mM NaCl, 1.5 mM MgCl_2_, 0.2 mM EDTA, 0.5 mM DTT and complete Ultra EDTA-free protease inhibitors (Roche, 05892970001). Samples were dialysed for 4 h into a minimum of 1–1,000 volume excess of 20 mM HEPES pH 7–8, 30% glycerol, 100 mM NaCl, 0.83 mM EDTA, 1.66 mM DTT, 0.2 mM PMSF and complete Ultra EDTA-free protease inhibitors (Roche 05892953001) using Slide-A-lyzer mini dialysis units with 3,500 MWCO cassettes (Thermo). Samples were divided into single-use aliquots (11 μl) and flash-frozen in liquid nitrogen for storage at −80 °C.

### Western blot analysis of overexpression

The protein concentrations of the lysates were quantified using a Pierce BCA Protein Assay kit according to the manufacturer’s 96-well format instructions (Thermo Fisher Scientific). The following overexpression (OE) values were used: SOX2 OE = 10.54 mg ml^–1^, SOX9 OE = 5.56 mg ml^–1^, SOX2 and SOX9 OE = 7.56 mg ml^–1^, GSC7 GSCs = 1.02 mg ml^–1^ and GSC7 differentiated cells = 0.43 mg ml^–1^. Verification of SOX protein overexpression, relative to untransfected hFib lysate, was resolved by SDS–PAGE and electroblotting onto a PVDF membrane for western blotting. The primary antibody incubations with goat anti-human SOX2 antibody (1:363, AF2018, Abcam) and rabbit anti-mouse SOX9 (1:2,000 AB5535, Chemicon) were performed at 4 °C for 16 h. The secondary antibody incubations with donkey anti-goat IgG-HRP (1:2,000 dilution of ab97110, Abcam) and goat anti-rabbit (1:2,000 of 32460, Invitrogen) were performed for 1 h at room temperature. Blots were visualized using SuperSignal West Pico Chemiluminescent substrate (Thermo Fisher Scientific) and HRP was visualized using a Bio-Rad ChemiDoc MP imaging system.

### Recombinant SOX protein production

His-tagged human SOX2 and SOX9 were expressed from a pET28a backbone (cloned by G. Roberts in the Soufi Laboratory) using Rosetta 2(DE3) pLys competent cells (Novagen).

SOX2 was expressed as previously described^47^. Cells in LB medium (1% Bacto-tryptone, 0.5% yeast extract and 1% NaCl), supplemented with 30 µg ml^–1^ kanamycin and chloramphenicol, were grown at 37 °C overnight. SOX9 was expressed in 2× TY medium (1.6% Bacto-tryptone, 1% yeast extract and 0.5% NaCl), supplemented with 30 µg ml^–1^ kanamycin and chloramphenicol, at 16 °C overnight. After expression, bacteria cell pellets were lysed, 100 ml buffer per 1 l of culture, in GuHCl Denaturing buffer (6 M guanidine-HCl, 50 mM Tris-HCl (pH 8) and 500 mM NaCl, 5% (v/v) glycerol) overnight. Lysate was sonicated for 200–300 s at 25–30 microns using a Soniprep 150 with intermittent chilling on ice. The supernatant was clarified by centrifugation at 18,000 rpm in a FLA21-8x50y rotor for 30 min. The lysate was further sonicated for 120 s, as above, then filtered through a 0.45 µm filter before loading into a HisTrapHP column (Cytivia) for affinity purification of His-tagged proteins using an AKTA pure system. The column was equilibrated with 5 CV of denaturing 10 mM imidazole buffer (6 M urea, 500 mM NaCl, 50 mM Tris-HCl (pH 8), 10 mM imidazole and 5% (v/v) glycerol), after sample loading was washed with 20 CV of denaturing 50 mM imidazole buffer (6 M urea, 500 mM NaCl, 50 mM Tris-HCl (pH 8), 30 mM imidazole and 10% (v/v) glycerol), and bound proteins were eluted with denaturing 300 mM imidazole (6 M urea, 500 mM NaCl, 50 mM Tris-HCl (pH 8), 300 mM imidazole and 10% glycerol). Peak fractions of the elution were analysed by SDS–PAGE, and samples with appropriately sized bands (40 kDa for His–SOX2, and 60 kDa for His–SOX9) were combined and desalted into 2 M urea buffer using a HiPrep 26/10 desalting column (GE Healthcare). The SOX2 desalting buffer comprised 50 mM Tris-HCl (pH 8), 240 mM NaCl, 10 mM KCl, 2 mM MgCl_2_, 2 mM CaCl_2_, 0.8 M urea, 30% (v/v) glycerol, 0.1% NP40 substitute, 0.05% Triton-X-100, 2 mM EDTA and 5 mM DTT resuspended in 1× PBS. The SOX9 desalting buffer comprised 50 mM HEPES NaOH (pH 7.5), 240 mM NaCl, 10 mM KCl, 2 mM MgCl_2_, 2 mM CaCl_2_, 2 M urea, 30% (v/v) glycerol, 0.1% NP40 substitute, 0.05% Triton-X-100, 2 mM EDTA and 5 mM DTT. The fractions corresponding to a dip in the absorbance at A_280_ were collected and visualized by SDS–PAGE before combining. Protein concentration was determined by SDS–PAGE, comparing to rAlbumin standards. The gels were imaged by detecting Coomassie blue staining using a Bio-Rad ChemiDoc MP imaging system. The resulting images were visualized, and the bands quantified using ImageJ 2.8/1.54i.

### Small-molecule inhibitor screening

E55 cells were screened using inhibitors included in the Kinase Screening Library (Cayman, 10505) as well as two YAP–TEAD pathway inhibitors, MGH-CP1 (HY-139330) and TED-347 (HY-125269). Around 18,000 cells were seeded per well in a 96-well plate and transduced with the corresponding AAVs at a MOI of 2,000,000 on the next day. Cells were incubated at 37 °C, 5% CO_2_. In the primary screen, the cells were treated with a single concentration of 3 μM per inhibitor at 24 h after transduction. After 48 h of inhibitor treatment at 37 °C and 5% CO_2_, the cells were detached and analysed using an Agilent NovoCyte Penteon five laser flow cytometer and FCS Express 7 Research (v.7.24.0030). The results were expressed as the fold change in mCherry median fluorescence intensity in the inhibitor-treated samples relative to the DMSO control. After performing the first round of screening, inhibitors that gave more than 20% changes were selected for the second round of screening.

In the second-round screen, the cells were seeded and transduced with AAVs using the same procedures as the primary screen. At 24 h after transduction, the cells were treated with 3 different doses (0.1, 1 and 3 μM), and incubated at 37 °C and 5% CO_2_ for 48 h. Cells were then detached and analysed using a Agilent NovoCyte Penteon five laser flow cytometer. Draq7 was used as the live/dead stain in both rounds of screening. The results of the second round screen were expressed as the fold change in mCherry MFI and as the percentages of mCherry-positive cells, each normalized to the DMSO control.

### EMSAs

Cy5-labelled SSE-7 bait for EMSAs was generated by PCR amplification of SSE-7 from the plasmid using Cy5-labelled primers (Sigma): 5′-ACTAGGTTACTGGTGCATGCTTGTCCTGCCTTTGAGAACA-3′ and 5′-ACCCAAACTATTGGAGCGAGAGAAAGGAAAGAAAGAGGTC-3′. The following primers were used for Cy5-labelled fragments for EMSA: ID4310: 5′-ACTAGGTTACTGGTGCATGCTTGTCCTGCCTTTGAGAACA-3′ and 5′-ACCCAAACTATTGGAGCGAGCTCATTTGAAGCAGAAGAAT-3′; ID4428: 5′-ACTAGGTTACTGGTGCATGCTCTAAATGGAGATCCTCCCA-3′ and 5′-ACCCAAACTATTGGAGCGAGACAGGAGGAAGTAGTAAATC-3′; ID0785: 5′-ACTAGGTTACTGGTGCATGCAAGAATGAACTGGGCCCAGC-3′ and 5′-ACCCAAACTATTGGAGCGAGTGAAAACCAGAGGCATCTCA-3′; and ID3836: 5′-ACTAGGTTACTGGTGCATGCTACCCTTCCAGGGGAGCAGT-3′ and 5′-ACCCAAACTATTGGAGCGAGAGAAAGGAAAGAAAGAGGTC-3′.

The binding to Cy5-end-labelled SSE-7 was analysed in native 1% agarose gels (12 × 13 × 1 cm), which were prepared in 0.5× TBE (45 mM Tris-borate and 1 mM EDTA). Gels were stored overnight at 4 °C before pre-running at 120 V (approximately 10 V cm^–1^) for 1 h. For affinity analysis, a 10 μl mixture typically containing 50 nM Cy5-labelled full-length or fragmented SSE-7, 100 ng µl^–1^ poly(dA:dT) (InvivoGen, tlrl-patn) and 0, 0.5, 1, 2 or 4 μl of hFib lysates, with and without SOX overexpression (5.56 mg ml for OE lysates and 3.47 mg ml^–1^ for hFib control lysates), GSC7 GBM stem, or differentiated, cell lysates (at 0.43 mg ml^–1^), or purified recombinant SOX protein (at 3.17 µM). For SOX2 and SOX9 in combination, equimolar amounts of SOX2 and SOX9 were mixed for 0.5, 1, 2, 4 and 8 µl of protein in total. Next, 8 µl SOX protein was added to 25 nM SSE-7 DNA in a 20 µl volume, and 20 µl of sample was loaded for gels, which were prepared in 1× binding buffer (10 mM Tris HCl pH 7.5, 1 mM MgCl_2_, 10 μM ZnCl_2_, 10 mM KCl, 1 mM DTT, 5% (v/v) glycerol and 0.5 mg ml^–1^ BSA). The mixtures were incubated at 20 ± 1 °C in the dark for 1 h using DNA LoBind tubes (Eppendorf). The entirety of each sample was then loaded onto agarose gels and electrophoresis was performed at 120 V for 3 h at 4 °C. The gels were imaged by detecting Cy5 fluorescence using a Bio-Rad ChemiDoc MP imaging system. The resulting images were visualized, and the bands were quantified using ImageJ 2.8/1.54i.

### Adult human cortex and brain tumour slice cultures

Tumour and non-tumour brain tissue were transferred to a 10-cm^2^ tissue culture dish with sterile PBS and placed on ice. Visibly damaged tissue was removed and the remaining tissue was transferred into a 35-mm^2^ dish with pre-warmed 3% SeaPlaque agarose (50100, Lonza). After cooling in ice, the block was removed and cut using a scalpel into an approximately 2 cm cube around the brain. Before starting to cut, a 6-well plate was prepared. In each well, we introduced one cell culture insert (PICMORG50, Millicell) and added below it 1 ml culture medium in basal NSC medium, Dulbecco’s modified Eagle medium: F12 supplemented with EGF and FGF (Life Technologies). The embedded brain was placed in a circular vibratome plate with glue. The vibratome (VT1000 S, Leica) plate was fixed in the platform and filled with PBS and penicillin–streptomycin (15140-122, Gibco, 1:100). Then, 300-µm-thick slices were cut, with vibrating frequency set at 10 and speed at 1. Each slice was transferred using a small brush onto the top of a Millipore culture insert. The platform was maintained cool at all times. The 6-well plate was placed in an incubator at 37 °C with 5% CO_2_.

#### Addition of virus

A 5 µl volume of virus was pipetted onto the centre of each tissue slice, using a 10 µl tip without touching the tissue. Three repeat doses of 5 µl virus was added to each slice at 5 min intervals (20 µl in total per slice). Slices were incubated for 7 days. The medium was replaced on day 3 or 4.

#### Immunostaining

The medium was removed and exchanged for 1 ml freshly prepared 4% PFA; 1–2 ml PFA was also placed gently on top to cover the slice. After 2 h, PFA was removed, brain slices were washed 3 times with PBS and transferred using a brush to a 24-well plate. Slices were incubated at room temperature for 1.5 h in blocking solution (0.5% Triton X-100 and 3% goat serum; Sigma-Aldrich) followed by incubation with primary antibody for 2 days at 4 °C. Sections were washed five times with PBS, the respective secondary antibody was applied in blocking solution overnight. The next day, slices were washed 5 times with PBS mounted and cleared in RapiClear 1.49 (RC149001, Sunjin Lab).

Images were acquired using an Opera Phenix Plus high-content imaging system (Revvity) equipped with a ×40/1.1 NA water-immersion objective. Image analysis was performed in Harmony software (v.5.2; Revvity). For the full pipeline see Supplementary Information. In brief, nuclei were segmented using DAPI with the common thresholding method, and the nuclear mean intensity of channels SOX2 (488) and mCherry (555) were then calculated. Thresholds to define positive cells were set for each tissue slice individually based on 3–5 regions of interest. Nestin was segmented using ‘find image region’ with channel 647, and positive cells were defined as those with nuclei in these regions. Analysis was performed for a single *z* plane. Data were exported and analysed using GraphPad Prism. Representative images of the segmentation can be found in the Supplementary Information.

### Zebrafish experiments

All embryos were obtained by natural spawning and collected in conditioned aquarium water in 0.00001% methylene blue. Embryos were raised at 28.5 °C in embryo medium (E3) on a 14 h light–10 h dark photoperiod and were treated with 200 μM *N*-phenylthiourea (Sigma) from 6 h post fertilization to inhibit pigmentation. Zygotes were injected at the one-cell stage of development. DNA constructs were created using the Tol2Kit system^48,49^. Approximately 2 nl of plasmid DNA (30 ng μl^–1^) containing Tol2-capped mRNA (20 ng μl^–1^), supplemented with 0.2% w/v phenol red (Sigma) to facilitate visualization of injected volume, was injected. To induce Akt1 overexpression in neural progenitor cells, a combination of Tol2-pDEST-NBT:DlexPR-lexOP-pA (20 ng μl^–1^) and Tol2-pDEST-lexOP:AKT1-lexOP:tagRFP (30 ng μl^–1^) plasmids was injected as previously described^50^. The newly generated tgSSE-7:eGFP was outcrossed to tg(Xla.Tubb:DsRed) and tg(olig1:mScarlet). Live imaging was performed using either a Leica MS205 stereomicroscope or a Leica SP8 confocal microscope with a ×40/NA 1.1 objective. Animal experimentation was approved by the ethics review committee of the University of Edinburgh and the Home Office in accordance with the Scientific Procedure Act 1986.

### AAV medium-exchange transduction assay

HEK cells were seeded into a 6-well plate a day before transfection, aiming for 60–70% confluence the next day. On the day of transfection, the culture medium was exchanged and HEK cells were transfected with RepCap, pHelper and AAV-ITR transgene plasmids using polyethylenimine. Two days later, conditioned HEK culture medium (now containing rAAV particles) was collected and centrifuged at 1,300 rpm for 4 min to remove any cells. rAAV-conditioned medium was transferred to HEK or GSC7 cells seeded at low density (10–20%) in 6-well or 12-well plates. Two to three days later, cells were analysed for transduction. For AAV1 in vivo experiments, we purchased research-grade viral vectors from Vector Biolabs (iodixanol gradient ultracentrifugation purified).

### AAV transduction assay in iPS cell-derived neurons

GSC7 and E55 cells were seeded in a 24-well plate format the day before transduction, whereas ioGlutamatergic neurons, ioGABAergic neurons and microglia (bit.bio) were seeded in a 48-well format before differentiation. After differentiation, cells were transduced with AAVs at a MOI of 5 × 10^5^. Cells were incubated at 37 °C and 5% CO_2_ for 3 or 10 days.

### Cell seeding and transduction for GCV killing assay

On the day of cell seeding, cells were detached using the method described above. Cells were counted using a haemocytometer and seeded into 96-well (1,000 cells per well in 50 µl), 24-well (30,000 cells per well in 500 µl) or 6-well (60,000 cells per well in 2 ml) Corning plate and placed in an incubator at 37 °C and 5% CO_2_. The next day, AAV stocks were thawed at room temperature, and an appropriate amount of virus stock was added to the required amount of culture medium to achieve the final MOI of 5 × 10^5^. Culture medium containing viral particles was added to cells without the replacement of existing medium. Cells were returned to a 37 °C and 5% CO_2_ incubator.

### Prodrug treatment

Lyophilized GCV was diluted in DMSO to achieve 100 mM stock concentration. GCV stocks were aliquoted and were stored at −20 °C for no longer than 1 month. To make a working stock concentration, GCV was diluted 1:100 in appropriate culture medium, and 20 µl was added to wells already containing 80 µl culture medium for a final concentration of 200 µM. As a negative control, DMSO was diluted 1:100 in appropriate culture medium to achieve a working stock. Next, 20 µl of working stock was added to wells already containing 80 µl culture medium. As a positive control, 20 µl DMSO was added to wells with 80 µl culture medium to achieve a final concentration of 20% DMSO.

### Incucyte live-cell imaging

To track cell proliferation and morphological changes during treatments, cells were monitored using an Incucyte live-cell imaging system. Whole-well imaging of Corning 96-well plates was performed every 4 h. Basic confluence scoring analysis software (Incucyte) was used to estimate confluence. Images at specific time points were extracted to verify cell confluence and morphology.

### MTT assay

On the day of assay, the culture medium was replaced with 0.3 mg ml^–1^ MTT solution (diluted in cell line-appropriate culture medium). Cells were placed in incubator at 37 °C and 5% CO_2_ for 3 h. After incubation, the medium was removed and 70 µl DMSO was added to each well. Each plate was kept in the dark at 37 °C for 20 min, shaking it occasionally. Before reading the plate, each well was visually inspected to make sure that all (formazan) crystals were dissolved. Plates were read with plate reader at 560 nm absorbance.

### Statistical analysis

Data analysis was performed using Microsoft Excel (v.16.23 for Mac), GraphPad Prism (v7) and RStudio (v1.1.456). Error bars are shown as the s.d. of the mean. For illustrations, BioRender and Adobe Illustrator 22.0.1 were used.

Furthermore, we used open-source programmes such as bedtools, GREAT, fastasplitter, HOMER and MEME for various analyses^45,46^.

### Tumour initiation by transplantation and intratumoral AAV delivery

All animal procedures were approved by the University of Edinburgh Animal Welfare and Ethical Review Body (AWERB) and conducted under UK Home Office licence (PPL number: PP8631583) in accordance with the Animals (Scientific Procedures) Act 1986 and ARRIVE guidelines. Male and female C57BL/6 mice (6–8 weeks old) were obtained from Charles River. Mice were housed in Individually ventilated cages with sterile bedding plus enrichment inside a pathogen-free facility on a 12-h light–dark cycle, at a temperature range of 20–24 °C and with relative humidity level of 45–65%. Transplantation experiments were performed as previously described^25^. We used 7-week-old male mice. Mice were transplanted with 200,000 NPE-IE cells in 2 µl PBS per mouse. NPE-IE cells have been previously described^25^ and have *Nf1* and *Pten* inactivating mutations, alongside EGFvIII overexpression and a GFP-Luc reporter construct. At 2 weeks, when tumours were visible by IVIS imaging (bioluminescence) and of similar size, AAV1-SSE-7–HSV-TK–IL-12 virus (1.2 × 10^13^ viral genomes per ml, Vector Biolabs) was delivered directly into the mouse brain tumour (2.5 µl per mouse) using a microsyringe linked to an injection pump, at a rate of 0.17 µl min^–1^ and 2.3 mm depth, higher than the previous depth of 2.4 mm for tumour cells. Each mouse received 3.00 × 10^10^ viral genomes. The following day after virus injections, GCV was intraperitoneally injected (500 µl per mouse at 2 mg ml^–1^) once daily for 20 days. Mice were imaged by IVIS imaging (PerkinElmer) once a week for luciferase signals. Animals were observed regularly for any neurological symptoms. Mice with tumours were culled based on the following criteria; (1) body weight loss of ≥20% relative to baseline (weights of mice were recorded once weekly); (2) abnormal neurological signs, including ataxia, head tilt, circling behaviour, seizures, paresis or inability to right itself within 3 s; (3) hunched posture with reduced locomotor activity and lack of reach response; and (4) any extracranial tumours ≥ 15 mm in diameter or ulcerated as per the Workman (2010) guidelines^51^. All mice reaching the humane end point were culled using the schedule 1 method. The end-point limits were not exceeded in any of the experiments.

### Immune characterization of mouse GBMs

Brains were collected and processed 5 days after treatment. First, they were microdissected under a Leica stereo microscope to avoid contamination with healthy tissue. Tissues were mechanically disaggregated using scissors, followed by 30 min of enzymatic digestion using a mix of 0.35 µg ml^–1^ Liberase TL (05401119001, Roche) and 0.23 µg ml^–1^ DNAse (101041590001, Sigma) in plain RPMI. After digestion, tissues were dispersed through a 70 nm cell strainer to obtain a single-cell suspension. After centrifugation, cells were resuspended in PBS for further flow cytometry staining. Cell suspensions were incubated for 10 min at 4 °C with a blocking mixture of mouse, rat and calf serum containing anti-CD16/32 blocking antibodies (clone 2.4G2, BioXcell, BE0307). Cells were washed with PBS and incubated for 20 min in antibody-containing staining buffer plus Fixable Viability Dye eFluor-780 (eBioscience, 65-0865-14) to distinguish live and dead cells. Cells were then washed and resuspended in fixation/permeabilization buffer (BD CytoFix/Cytoperm; 554714 or FOXP3 Transcription Factor Staining Buffer set, eBioscience) followed by intracellular target staining.

### SABER–FISH detection of transgene copy number

SABER–FISH was performed as previously described^52,53^. Non-overlapping 36 bp probes were designed to target the HSV-TK transgene, and probes were extended using primer exchange reaction to approximately 500 bp. For imaging, cells were first stained with mCherry,then fixed with PFA and subsequently processed for immuno-SABER–FISH^54^.

### Assessment of the fraction of the tumour cell population that activates AAV1-SSE-7–mCherry

AAV1-SSE-7-mCMV–HSV-TK-V5–mCherry was injected at around 3.58 × 10^14^ viral genomes per ml and 7.2 × 10^13^ viral genomes per ml (1:5 dilution) in PBS. AAV was injected into tumours as described above. After 7 days, tumours were imaged using a stereomicroscope and surgically excised.

Each tumour sample was added to 1 ml digest mix (RPMI + 1 mg ml^–1^ collagenase IV, 12.6 µg ml^–1^ DNase I and 1% penicillin–streptomycin) and incubated for 1 h in a shaking incubator at 40 rpm at 37 °C. Samples were filtered through a 100 μm cell strainer, washed with 9 ml FACS buffer and centrifuged at 250*g* for 15 min at room temperature. The pellet was then resuspended in 1 ml 37.5% Percoll in PBS and gradient centrifuged at 900*g*, room temperature, for 12 min with no brake. The supernatant was discarded, and the bottom layer of cells resuspend in 500 µl PBS and filtered into a FACS tube. Cells were stained with DAPI as a live/dead stain and analysed using a BD LSR Fortessa cell analyser (four lasers, BD Bioscience).

### Reporting summary

Further information on research design is available in the Nature Portfolio Reporting Summary linked to this article.

## Online content

Any methods, additional references, Nature Portfolio reporting summaries, source data, extended data, supplementary information, acknowledgements, peer review information; details of author contributions and competing interests; and statements of data and code availability are available at 10.1038/s41586-026-10329-6.

## Supplementary information

Supplementary Information Supplementary Sections 1–8, including Supplementary Tables 1 and 2.

Reporting Summary

## Source data

Source Data Figs. 1 and 3–5 and Source Data Extended Data Figs. 1–4 and 7–13

## Figures and Tables

**Extended Data Table 1:**
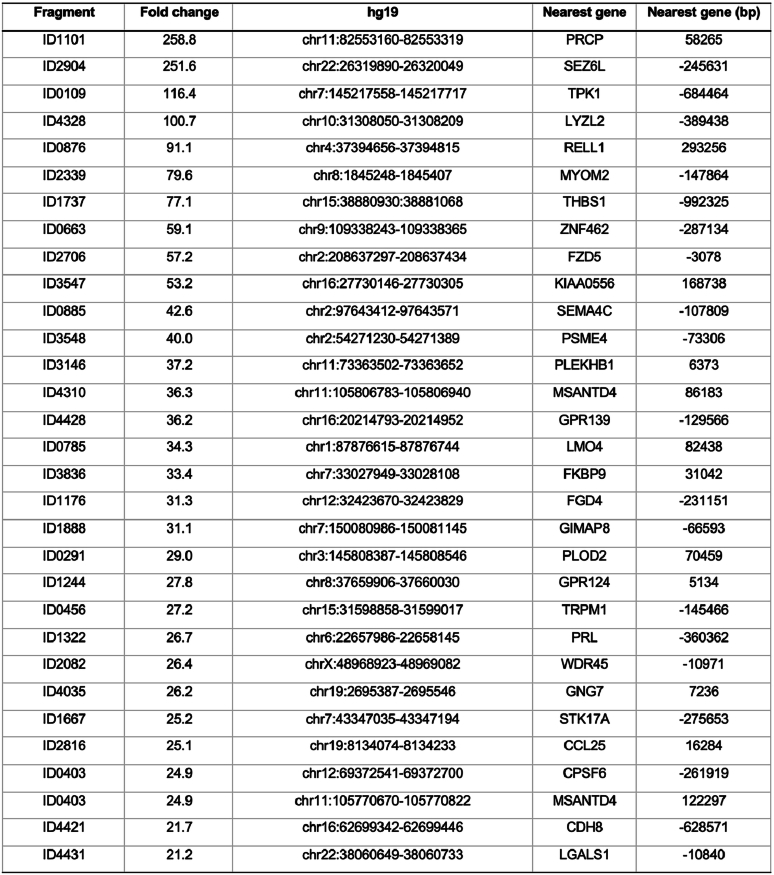
Predicted target genes and distance from enhancer fragment to transcription start site (TSS)

## Data Availability

The scRNA-seq data have been deposited at the European Nucleotide Archive (ENA) (accession number: PRJEB81816). The project number for ChIP–seq data is PRJEB107008. For ChIP–seq, raw (fastq) and processed (bigwig and narrowPeak) files have been deposited and made public (https://www.ebi.ac.uk/ena/browser/view/PRJEB107008). The ENA does not take processed files; therefore, these have been uploaded to BioStudies, which links to the same ENA accession (PRJEB107008), otherwise they are at https://www.ebi.ac.uk/biostudies/studies/S-BSST2733. All the codes and processing steps for scRNA-seq, SCENIC analysis and ChIP–seq data are available at GitHub (https://github.com/alhafidzhamdan/sse_gene_therapy). The GitHub repository includes some processed files, including all the narrowPeak files and consensus sets; using these in conjunction with the .Rmd document for reproduction of Fig. 2. This also includes a link to the SOX2 and SOX9 tracks on the UCSC browser. The linked and minted a repo is available at Zenodo (https://zenodo.org/records/18676096)^55^. Source data is provided with this paper.
