# Supplementary material for: Synthetic super-enhancers enable precision viral immunotherapy
Source: Nature. 2026 Apr 8;653(8113):232–41. doi: 10.1038/s41586-026-10329-6 (PMC13149004; doi:10.1038/s41586-026-10329-6)
Supplement: Supplementary file 1 — Supplementary Sections 1–8, including Supplementary Tables 1 and 2. [file 41586_2026_10329_MOESM1_ESM.pdf]

nature portfolio

https://doi.org/10.1038/s41586-026-10329-6

Supplementary information

Synthetic super-enhancers enable precision viral immunotherapy

In the format provided by the authors and unedited

Supplementary Information Guide

Title: **Synthetic super-enhancers enable precision viral immunotherapy**

Authors: Ute Koeber[1, 7*], Mantas Matjusaitis[1,7,8*], Neza Alfazema[1,7], Katharine Furlong[1], Zeyu Wang[1,7], Rachel White[1,7,9], Alhafidz Hamdan[1,6,7], Pooran Dewari[1,7], Gregoire Morisse[5,7], Mariela Navarette[2], Rosie Willis[1,7], Jin Wang[5,7], Michelle P. Clark[1,7,9], Carla Jacinto de Sousa[1,7,9], Hei Ip Hong[1,7], Shahida Sheraz[1,7], Ben Southgate[1,7,9], Justyna Cholewa-Waclaw[1,7], Jacek Mendrychowski[1,7], Vera Kho[1,7], Sabine Gogolok[1,7], Gillian M. Morrison[1,7], Felipe Galvez Cancino[2,10], Faye Robertson[1,7], Anna Williams[1], Susan J. Rosser[3], Paul Brennan[1,4,7], Dirk Sieger[5 ,7], Abdenour Soufi[1], Sergio A Quezada[2] and Steven M. Pollard[1,7,#]

Please find below the following Supplementary Information for Koeber et al.

1. **Supplementary Table S1** | DNA sequences of the top 32 enhancer fragment hits (Related to Fig. 1)
2. **Supplementary Table S2** | Antibodies used in this study for immune profiling.
3. **Flow cytometry gating strategy for single cell QC analysis and sample processing** (Related to Fig. 3h and Extended Data Fig. 5 and 6a). E28 is shown.
4. **Unprocessed EMSA gel images** (Related to Fig. 2)
5. **Image analysis, segmentation and quantitative analysis** (Related to Fig. 4 and Extended Data Fig. 9)
6. **Harmony software image analysis pipeline** for human tissue (**Related to Fig. 4 and Extended Data Fig. 9)**
7. **Western immunoblots uncropped gels** (Related to Extended Data Fig. 2g and Extended Data Fig. 10d)
8. **Typical example of flow gating** for all tumours assessed for GFP and mCherry costaining (GFP tumours cells and mCherry transgene, respectively). Related to Extended Data Fig. 13j

ID1101 chr11 82553161 82553319

GTGAGTGTGACTCACATGCTGGCATCAGAGCCCCTCTTAGAGCTTAGAACACTGGGCCTCTGTCAGGCAAAGAAAAGGAGTCCCTATGAA
TAGCCATCTCCACAAATAGCAGTTACTGTCACTTCTTAAAAGACACAAGCATGGCTGGAGACCGGCACTG

ID2904 chr22 26319891 26320049

TGCATGTTCTTGGGAAAAAGTGGTATTTAATGAAGGCGCTTTGAGCTGAATCAAAGAGCCTCATTCTTCTGAAAGTCCTTCTTCCTAGTCTC
TGCATCCGGCTCCTATCAAAGGAAATCCACAGCTTTGGCTTTTCGTTGCTGTTGTCATGGAAATCGTT

ID0109 chr7 145217559 145217717

GAAATTGGATCCTGAGGAGAATGTCACCACCTCTTTGACAATAACTAGCTTTCAGAGCTGAACACAGTGAATCTGGATACTGTGTATATGT
TTAAATGAGTTAGGAAATGACATCACATTATTTATTTCTTCTTTTGTGACTTAGTAAATTGACTTGGCC

ID4328 chr10 31308051 31308209

TACAATCTATTCTCCATAGTCATCAGAGTTTCTTTAAAGGCACGGTTCAGACAATGGCACCCTCTGTTCCAAACCCTCCAGGGCTTCCTATT
TCACTTAGCATAAAAACCAAAGTCCTCACAATGACCGACAAGTCTTCACACAATGTGCCCCTTCATTG

ID0876 chr4 37394657 37394815

GAGCAGGCAGTCATTTGGTTCCCTTCTCTGAATGCATTGGCTCTGATCCACGGCGCCTCAGGAAGTGAAAGGGAGCAGGCAGTGAGGCC
AAGCCATGAATGTTGGGCCCTTTTCTTAGAAAACAAAGGGTTCACTTTAGGCTAGGAGGAAACATCCGGGG

ID2339 chr8 1845249 1845407

GTCGGCGGAACTGAGTCTCCTTTGTGGAGCCCCTCATTTGCATGATAAACCCCGGGGACAAAGTGTGTTCTTGTTACTTGGACTGTTTCTT
GGAAACTGCTGCTACCTGGAGCGAGTGAACACAGGGCAAGTATTCTCGCCCACAGCAACGTGTCATTGA

ID1737 chr15 38880931 38881068

CAACGTTTTTGGCAGATGCAGCTCTTTCTGAACAGTAAGTCATTGTTGAGTGACATGAATAGGACGAGAGCTACAGAAAATGAAGGTTTCA
AAAGATTGTCTAAAACAAAAGTCCTTTCAGTCCCGTATTCAGAGTT

ID0663 chr9 109338244 109338365

ATAAATTCCGATTAGCCAAGGAAGCTCTGAGTCTAAATACCAGCAAAGCTCAGCCCCTGCAAGTGGGGACGAATTATTTGAAAGGTCATC
CTAGAGACACTGAACAAAGGGCCTCTAGCAGCT

ID2706 chr2 208637298 208637434

CCAATGCCAAAGCAGTTTACATTTTACAGATGCCATGGCAACCGTCAGGAAGTTACCCTGTATGGTCTAAGAGGGGGAGGAACCCTCAGT
TCCGGGAATTGCCCGCCCCTTTCCCGGAAACTCATGAATAATCCACCC

ID3547 chr16 27730147 27730305

CGTTCCCCCTTAACAAAAGCCACCTCCTCCCTCCTGAAAGGCACCATTGGTCACCAAAACAAGTCCCCTTTGTCCTCCACCGCAGCTGGA
GCCTGGGCCTTCCATCTCCTGGGGGCCAATAAAACCCCAGATCAGCCAATCCTCCCTCCCTGGCTCTGTCA

ID0885 chr2 97643413 97643571

CAGCAAAGGCTGCACGCAATGGGGGAGGGGGAAGGAGGGAAGGATATTTCCTCCTTTTTAGATCAGTTTCTTTTCTGAGCACAAGTGCTT
TCTGTGTCATCCTAACAATGTAAGAGCCCACTAAGTTTAGGACGCATAATACCGGCCTTTGTTCTACTCC

ID3548 chr2 54271231 54271389

TCCAAACACTCCCCTGCAACAGATGTACACACAGAGCCCATTGTTTTCTCTAGAAATGTAAACTGCACGTCACAGTTACTCCATACACACC
ACAAACAGACTCCCTTTGTTCTCAGGATATGTGTTGACACAAGAATATGTCATCCTCTCAGCTCCAGAC

ID3146 chr11 73363503 73363652

TGAAAGAGATGGGTGTGTGGGAGTGGGGGAAGCTGAGCTCCAGGCAGACAGCACAGAGCCGCCTTGTCCAACCCAGCCAGGTGCCATG
GCAACCAACAAAGGCCCCATTGTCCCGTGTCTGCTGGGCCTTCCTGAGGCTCTGGGGTCAGAGTCCCGGTT

ID4310 chr11 105806783 105806940

TTGTCCTGCCTTTGAGAACACTGGAAGGGCACTGAAGGCTGACCAGCATGCACCTCACCCCACCTCCTCTCTGTCTCATCCTCAGCCATC
TAAGCGGGAGACTCATCAGGCTTAAATTCTCATTCTTATCCAAGATTCTTCTGCTTCAAATGAG

**ID4428**	**chr16**	**20214794**	**20214952**

TCTAAATGGAGATCCTCCCATAAAGGGAGTTCTGTTTGGGCTTCCCTTGGAAGTCCATTGAAAGCGTTTCACATTTCAATGCTTCTTTACAT
TCTCCTCTAGAATGAATCTGGAAGGGCCATTTCATTCCAGCCCTATCAGATTTACTACTTCCTCCTGT

**ID0785**	**chr1**	**87876615**	**87876744**

AAGAATGAACTGGGCCCAGCTGGGCATTTGCATCAAAGTGCCTCACCAGAGTTCCCAGCTGCTCTGGCAGAGTGCCCAGGCTTGCCCCA
GAGCCCAAACAGAATGCTCTTTGAGATGCCTCTGGTTTTCA

**ID3836**	**chr7**	**33027949**	**33028108**

TACCCTTCCAGGGGAGCAGTTTCCTGGCTGTGTCGTCAGATACTGTGGTTTACGCTGTTCCGGAGTCTGGAGGTCTGTGTGCCTTTGATC
TCTTTCCCAACTAATTTGCACAGCTGCTGTGGAGGCCCTCATGAATGACCTCTTTCTTTCCTTTCT

**ID3765**	**chr11**	**105806783**	**105806940**

TTGTCCTGCCTTTGAGAACACTGGAAGGGCACTGAAGGCTGACCAGCATGCACCTCACCCCACCTCCTCTCTGTCTCATCCTCAGCCATC
TAAGCGGGAGACTCATCAGGCTTAAATTCTCATTCTTATCCAAGATTCTTCTGCTTCAAATGAG

**ID1176**	**chr12**	**32423670**	**32423829**

ACAAACTAAACACAGACATTTTCTCAATTAGTCATTTTCAGGTTTATTGCCTAAGATGCTAATTGAAATTCAGGGCAGGCAAAAATACATCAT
TGGCCTTTGTATATCTTTGAAAGGCCTTCTGATGGGACCTTGCTCCGTATAAAGGCTTCTCTTCTCT

**ID1888**	**chr7**	**150080986**	**150081145**

GACCTTGCCTCTTTCTGGCCTGTGGCCTTTCTCCTCCCCCAGCTGCTGTCTGGCCGCCCCACCCCCAAGTCTCTGCCACAACCAAAGCTC
CTTTGTTGCCTCCCCCTGCCCCGGGCCTCTCCACCTGCTGCTAGCTCAATCCCCTCTTTGTGTGGTTGGT

**ID0291**	**chr3**	**145808387**	**145808546**

CATTCTTTGCAGATAGGAAACAGTTTGCTATCTGAGAGACTGGCCAAAAACCTTACTTTCAAGTAAAATAGGGCTCTATTCATAAGCTCCTA
CATGTACACAAGTTACTCTTTATACTCCTTTCAGACAATGATAAGAATAGAAACTATTTAATTCAGGA

**ID1244**	**chr8**	**37659906**	**37660030**

ACAGCAGCCAGAGGAAGTGGCTGTGTACAGAGAGCTTTTGTGAGAGAAGAGGGGGAGGGGGAAAGAGCAAAGAAAAAGAAGGAAAGAA
GAGAGGGAAGAGGCCAGAGACAGAGAAGGCCAGAGCA

**ID0456**	**chr15**	**31598858**	**31599017**

ATCACAAAGTGGTTAGCATGCAGGGGAGTGCGCCGGTCCCCCGCCTGTATTTGCATGACAAAAGCGCCTGCCCAACGGCTCTCGGTGGC
TTTGTTGAGAGCTGCAGCCTCGCTCAGGCTGAGCCTTGGGAATTGTTCCCCGACATTGGTTGTTCCTGGGC

**ID1322**	**chr6**	**22657986**	**22658145**

GGGCAACAAAGGATGCCATGCAAAGCAGCTCAGAACTTGAGTTGTTTCAACAGATGCTGTTTTATGTTTTGCCAAAGAATGGCCCCTTTTA
TTTCTGTCATGAATGCAGTCTTTCTTTCAGTTTCTAAATGTTTGCCAGTATTAACCCTAATCCTTATCT

**ID2082**	**chrX**	**48968923**	**48969082**

ACAGAAGCCGTTGCTGCAGTAACCACTGCCGCAAGAGCAACTTTGGCAGCAGGCAGGTGCCAGGCAGCAAGCTGAGAGAACACTGACA
GATTCAAAGTGTCTTCAGATACAAGGCTTTGTTTGTGCACAGTGAACAAAGGGCATATTGGGAAGGCCTCC

**>ID4035**	**chr19**	**2695387**	**2695546**

CCCAGACTGCCATCTCCACGGCCCCCACACCAGCACACAGATGCCTCATTCACGTGGTGCTCCCAGGGCCTGGCACCAGTTCTGAGGCT
TAAAATAGGCACACAACGGGCCTTTGTCGGATAGAGGAAGGAAGGAAGGAGCGTTTCAGACAAAGGAGACA

**ID1667**	**chr7**	**43347035**	**43347194**

ACCCTTTGCAGAGAGGAAAGAGAAAGGAGGTCTCCTGTTGGGGAGATCTGCTTTAGAGGTTAGGAAGAATGGTGGAGTTGTAAGACATTG
GAATCACTGTCTAGGAAGGACAGTGGCCTTTTATCCAGGGCTATTTAAAAACAGACAGAGCTTCATCTAT

**ID2816**	**chr19**	**8134074**	**8134233**

TTCCTGCATCTGGCCCTCTTTGAGAGCCCCTTGTGTGCCAATGACAAACCCTTCTTTATTTAAGCTCCTCTGAAGTGCCTTCCTGGTCTTG
GCAGCTGGACAGGGCCCTGAATAAGACAGCGAGCCTCAATGGCAGGCTACCCCAGGGCTGGGTGACGTA

**ID0403-1**	**chr12**	**69372541**	**69372895**
TGCCTCTCTTCCATTGGCATTGGAGTGGCTATAGGCCTTTGGAGGACCTGGCAAAGGGGAAGTTGATTTAGGGGTTCATTGAATTGAGGG GAAGTATGTAAGTGGTTTGCAGTCACTTCCTTGTGTGGTACTCTTGTATAGTAATGATTTCCTGGAATA			
**ID0403-2**	**chr11**	**105770670**	**105770822**
ATGTTAAACAGTGAAATCATCAGCAAAAAGCACAAAACTGCAGAAAACATGGCACTAAATAGAACACAAAAAGGACACTTGTTTACAGTAT GAGAGCAGAAACAAGGAAACAGTGTTTCCTTGTTCAGCCACAGCTGGGACCTTGTGTGT			
**ID4421**	**chr16**	**62699342**	**62699446**
GCATTCATTTTACAGCTGGGGTAAGCTTTCTCTGAAATGCAGCTGGAATAGGCATTTCAAAGGCCCACAGTGTTATGCAAATGCTTCTATT CTCCATGTGGGCGG			
**ID4431**	**chr22**	**38060649**	**38060733**
AAATGGTGATTATCATCATTTGTTCAGGGACATTAGCATGAAGGCCGAATGTTCTGGCACAAAGACACGTCTGTGGGACAGGCAC			
**ID3495**	**chr1**	**87876615**	**87876744**
AAGAATGAACTGGGCCCAGCTGGGCATTTGCATCAAAGTGCCTCACCAGAGTTCCCAGCTGCTCTGGCAGAGTGCCCAGGCTTGCCCCA GAGCCCAAACAGAATGCTCTTTGAGATGCCTCTGGTTTTCA			

1. **Supplementary Table S1 | Sequences of the top 32 fragments.**

**Mouse Flow cytometry antibodies**	**Supplier**	**Cat number.**	**Antibody dilution**
NK1.1	BD Biosciences	PK136/Cat #564144	1/300
CD4	BD Biosciences	GK1.5/ Cat# 564667	1/300
CD45	BD Biosciences	30-F11/ Cat# 565710	1/200
CD11B	BD Biosciences	M1/70/ Cat# 565080	1/200
CD3	BD Biosciences	17A2/Cat# 564380	1/300
CD8a	BD Biosciences	53-67/ Cat# 612898	1/300
FOXP3	eBioscience	FJK-16S/ Cat#48577382	1/100
CD62L	BD Biosciences	MEL-14/ Cat#563117	1/400
TIM-3	Biolegend	RMT-23/Cat#119721	1/100
CD27	Biolegend	LG.3A10/Cat# 119721	1/200
GITR	BD Biosciences	DTA-1/Cat# 563390	1/200
CD44	Biolegend	IM7/ Cat#103059	1/200
CD25	Biolegend	PC61/Cat#102017	1/200
LAG3	eBioscience	ebioC9B7W/Cat#46-2231-82	1/100
TCF7	Cell Signalling Technology	C63D9/ Cat#14456S	1/100
PD-1	BD Biosciences	J43/ Cat#562523	1/200
Ki67	eBioscience	SolA15/ Cat# 25-5698-82	1/200
TOX	Miltenyi	REA473/ Cat#130-118-335	1/100
Granzyme b	BD Biosciences	GB11/ Cat#560213	1/200
CD24	BD Biosciences	M1/69/ Cat# 564664	1/400
LY6G	BD Biosciences	IA8/Cat# 565707	1/100
CD45	BD Biosciences	30F11/Cat# 624287	1/400
CD68	Biolegend	FA-11/Cat#137017	1/200

Cd16.2	Biolegend	9E9/Cat #149531	1/200
LY6C	BD Biosciences	AL-21/Cat# 563011	1/200
CD206	Biolegend	C068C2/Cat#141723	1/400
MHCII	Biolegend	M5-114-15-2/Cat#107643	1/200
CD11c	Biolegend	N418/Cat#117336	1/100
CD49D	Biolegend	R1-2/Cat#103611	1/200
XCR1	Biolegend	ZET/Cat#148208	1/200
CD16	Biolegend	S17014E/Cat#158003	1/200
PDL1	Biolegend	10F.9G2/Cat# 124324	1/200
CD64	Biolegend	X54-5/7.1/Cat#139314	1/200
CD32b	Thermo Scientific	AT130-2/Cat# 17-0321-82	1/200
Ki67	Invitrogen	SolA15/Cat# 56-5698-82	1/200
IFN-γ	Biolegend	XMG1.2/Cat#505813	1/100
IL-2	eBiosciences	JES6-5H4/Cat#17-7021-82	1/100
TNF-α	Invitrogen	MP6-XT22/Cat#25-7321-82	1/100

2. Supplementary Table S2 | Antibodies used for immune cell characterization of tumours.

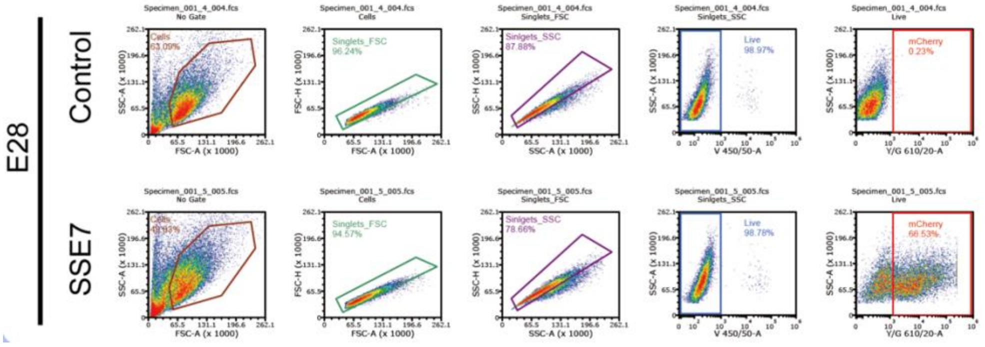

Gate	# of Events
No Gate	23013
Cells	14518
Singlets_FSC	13972
Sinlgets_SSC	12278
Live	12152
mCherry	28

Gate	# of Events
No Gate	34920
Cells	17399
Singlets_FSC	16454
Sinlgets_SSC	12942
Live	12784
mCherry	8505

3. Flow cytometry gating strategy for single cell QC analysis and sample processing (Related to Fig. 3h and Extended Data Fig. 5 and 6a). E28 is shown.

G7 lysates on FL SSE7 (Fig. 3d) n=3

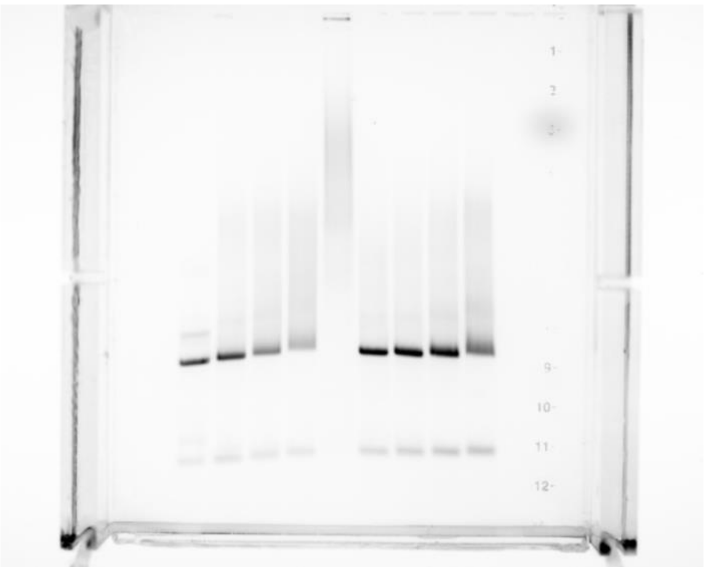

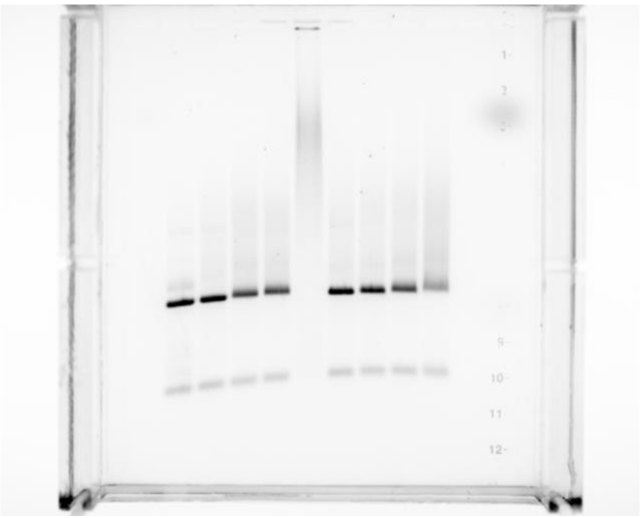

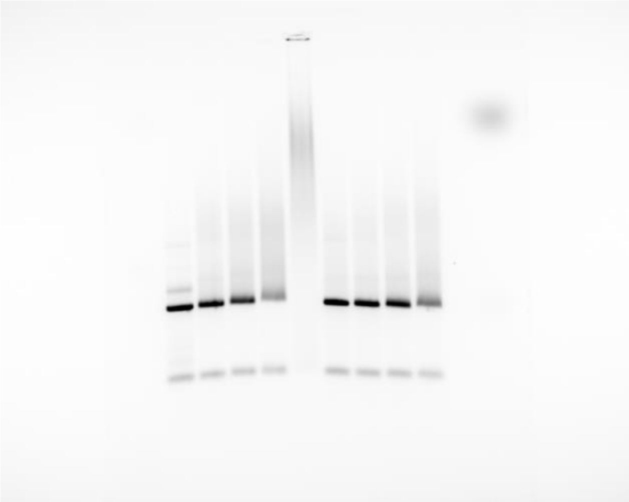

hFib OE lysates on FL SSE7 (figure 2f) n=3

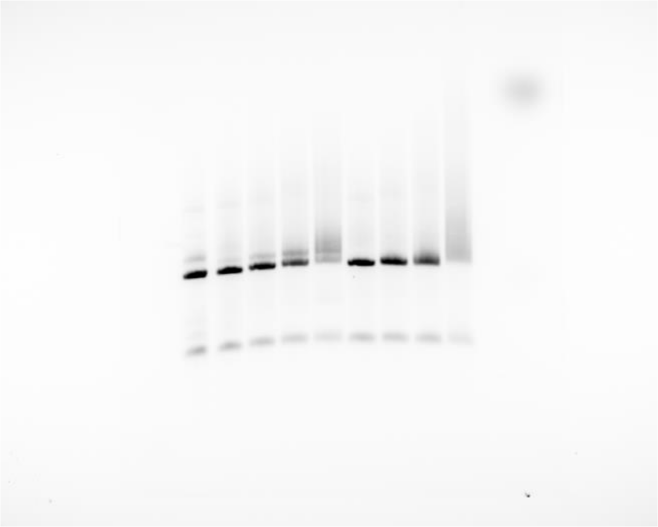

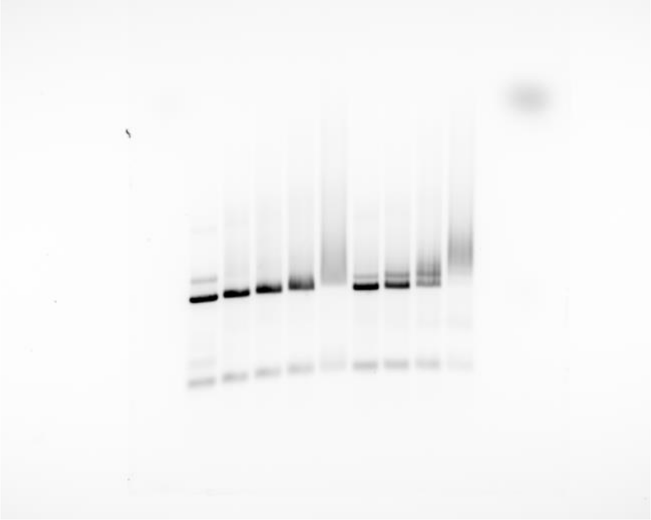

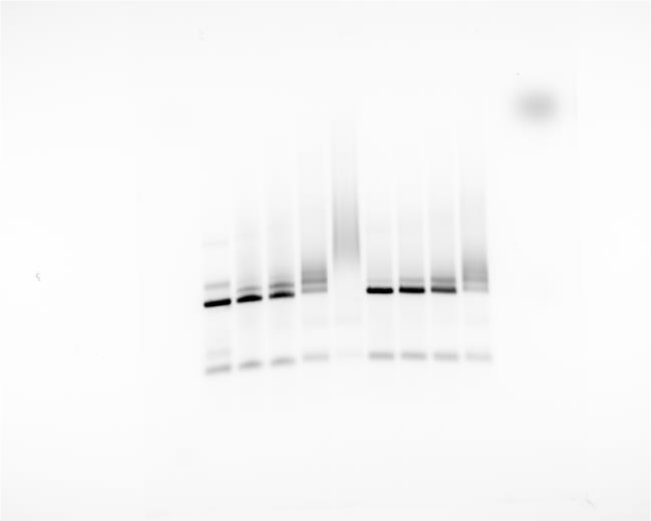

HFib lysates figure 2f

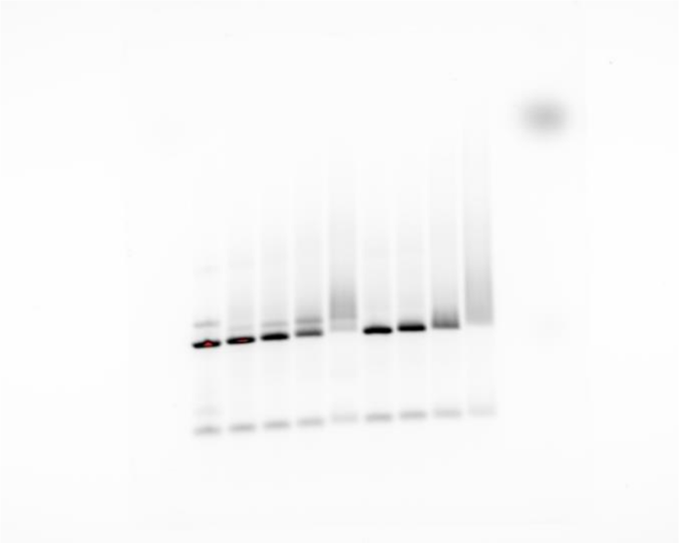

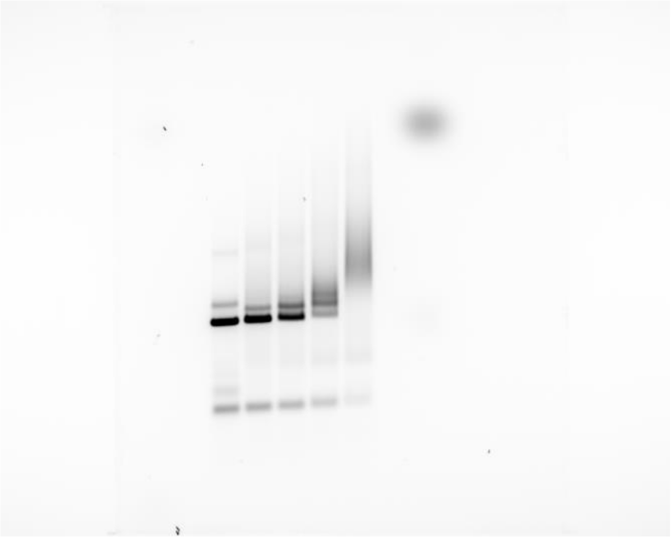

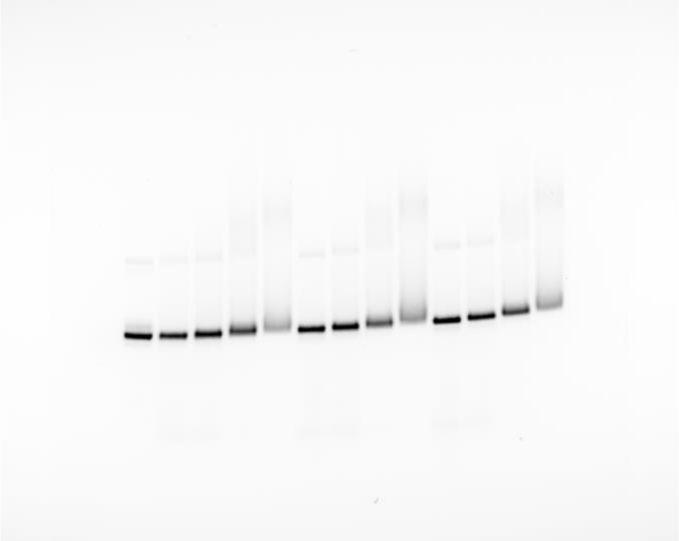

Recombinant protein on FL SSE7 figure 3f (n=3)

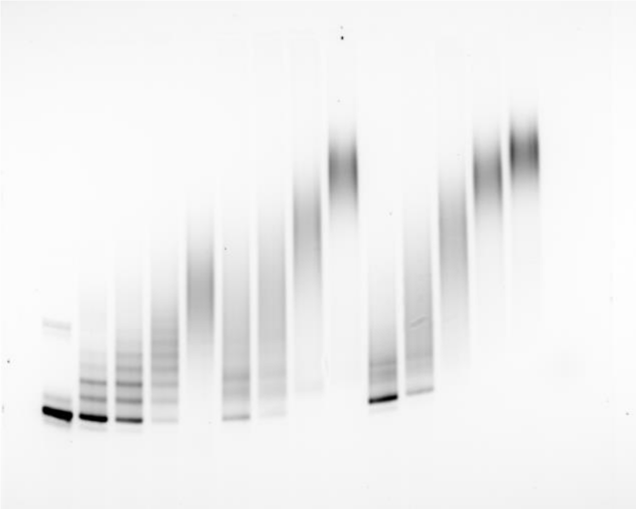

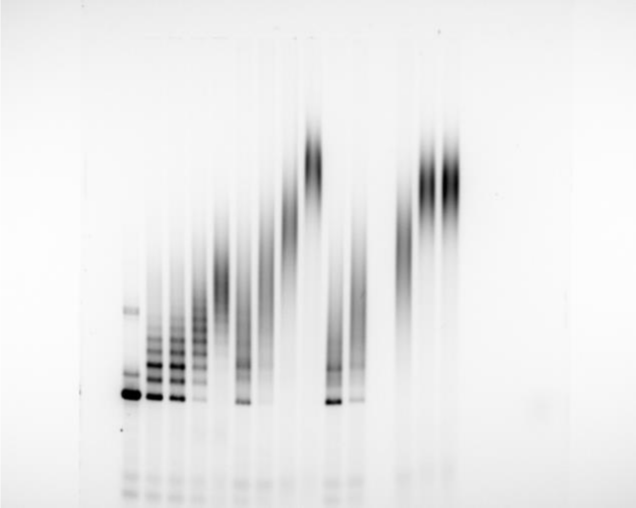

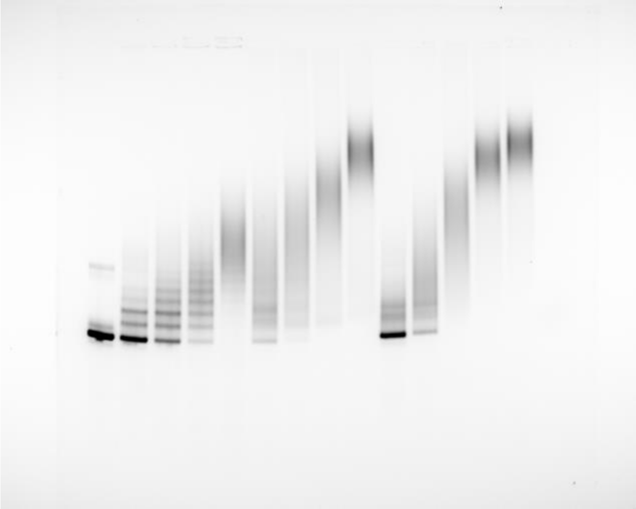

Recombinant proteins on FGF4 extended figure 4f (n=1)

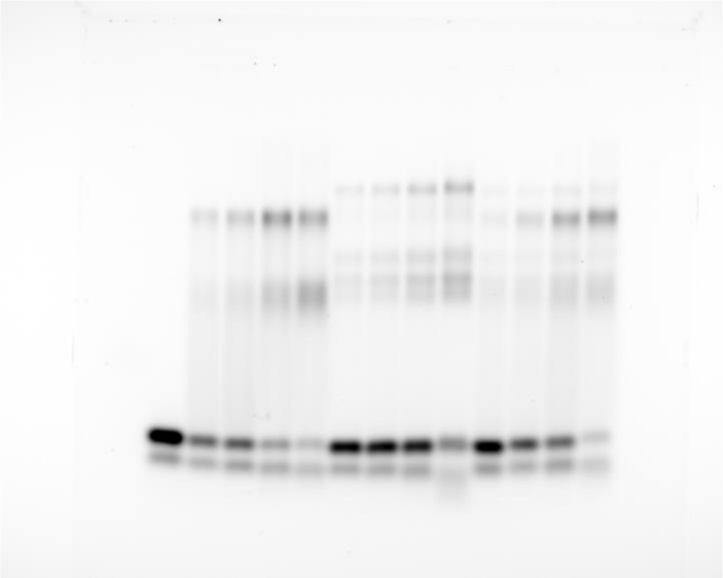

Recombinant proteins on the constituent fragments of SSE7 Figure 2g (n=1)

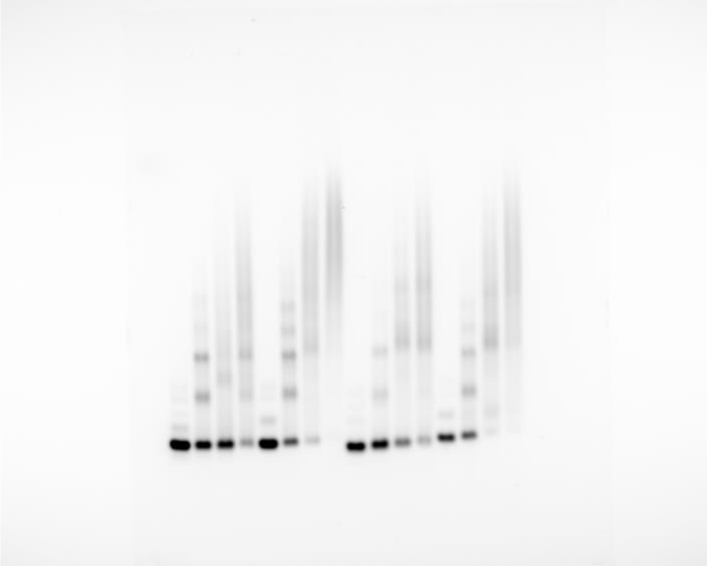

4. Uncropped full gel images for EMSA (Related to Fig. 3d-f)

Example of cell scoring/segmentation

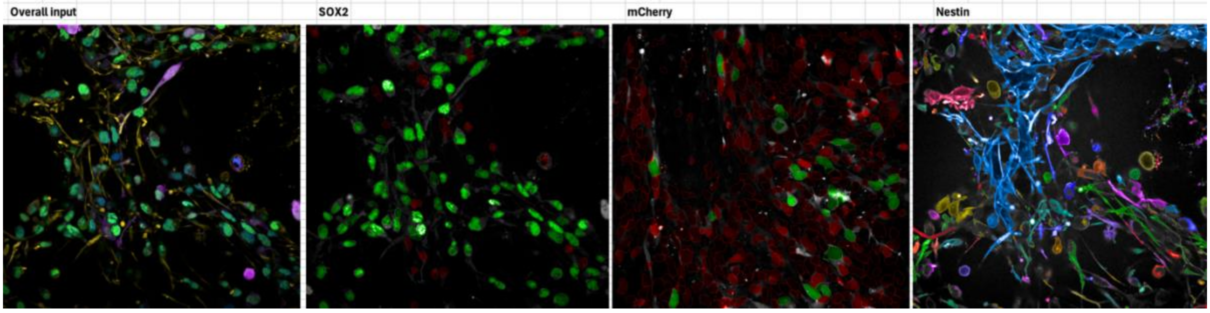

5. Image analysis, segmentation and quantitation for human tissue slices | Example of cell segmentation/scoring.

Input Image	Input
	**Channel group :** 1<br>**Sequences :** ALL<br>**Flatfield Correction :** Basic<br>Brightfield Correction<br>**Stack Processing :** Individual Planes

Find Nuclei	Input	Method	Output
	**Channel :** DAPI<br>**ROI :** None	**Method :** C<br>Common Threshold : 0.3<br>Area : > 20 µm²<br>Splitting Coefficient : 7.0<br>Individual Threshold : 0.4<br>Contrast : > 0.15	Output Population :<br>Nuclei

Calculate Morphology Properties	Input	Method	Output
	**Population :** Nuclei<br>**Region :** Nucleus	**Method :** Standard<br>Area<br>Roundness	Property Prefix :<br>Nucleus

Calculate Intensity Properties	Input	Method	Output
	**Channel :** DAPI<br>**Population :** Nuclei<br>**Region :** Nucleus	**Method :** Standard<br>Mean	Property Prefix :<br>Intensity Nucleus DAPI

Select Population	Input	Method	Output
	**Population :** Nuclei	**Method :** Filter by Property<br>Nucleus Area [µm²] : > 26<br>Intensity Nucleus DAPI Mean : > 0<br>Boolean Operations : F1 and F2	Output Population :<br>Nuclei Selected

Select Population (6)	Input	Method	Output
	**Population :** Nuclei Selected	**Method :** Common Filters<br>Remove Border Objects<br>Region : Nucleus	Output Population :<br>whole_nuclei

Find Image Region	Input	Method	Output
	**Channel :** Alexa 647<br>**ROI :** None	**Method :** Common Threshold<br>Threshold : 0.58<br>Split into Objects<br>Area : > 0 px²<br>Fill Holes	Output Population : Image Region nestin<br>Output Region : Image Region

Calculate Intensity Properties (2)	Input	Method	Output
	**Channel :** Alexa 488<br>**Population :** whole_nuclei<br>**Region :** Nucleus	**Method :** Standard Mean	Property Prefix : Intensity Nucleus Alexa 488

Calculate Intensity Properties (3)	Input	Method	Output
	**Channel :** Alexa 555<br>**Population :** whole_nuclei<br>**Region :** Nucleus	**Method :** Standard Mean	Property Prefix : Intensity Nucleus Alexa 555

Select Population (2)	Input	Method	Output
	**Population :** whole_nuclei	**Method :** Filter by Property<br>Intensity Nucleus Alexa 488 Mean : > 1900	Output Population : Sox2 +

Select Population (3)	Input	Method	Output
	**Population :** whole_nuclei	**Method :** Filter by Property<br>Intensity Nucleus Alexa 555 Mean : > 1700	Output Population : mcherry +

Select Population (4)	Input	Method	Output
	**Population :** whole_nuclei	**Method :** Select by Mask<br>Region : Nucleus<br>Mask Population : Image Region nestin<br>Mask Region : Image Region<br>Select by : Geometrical Center	Output Population : nestin +

Calculate Intensity Properties (4)	Input	Method	Output

	**Channel :** Alexa 647<br>**Population :** nestin +<br>**Region :** Nucleus	**Method :** Standard Mean	Property Prefix : Intensity Nucleus Alexa 647
**Select Population (5)**	**Input**	**Method**	**Output**
	**Population :** whole_nuclei	**Method :** Filter by Property<br>Intensity Nucleus Alexa 488 Mean : > 1900<br>Intensity Nucleus Alexa 555 Mean : > 1700<br>Boolean Operations : F1 and F2	Output Population : Sox2 mcherry +
**Select Population (7)**	**Input**	**Method**	**Output**
	**Population :** whole_nuclei	**Method :** Filter by Property<br>nestin + : > 0<br>Intensity Nucleus Alexa 555 Mean : > 1700<br>Boolean Operations : F1 and F2	Output Population : Nestin + mCherry+

**Define Results**	**Results**
	**Method :** List of Outputs<br>**Population : Image Region nestin**<br>Number of Objects<br><br>**Population : whole_nuclei**<br>Number of Objects<br>Sox2 + : Mean+StdDev<br>mcherry + : Mean+StdDev<br>nestin + : Mean+StdDev<br>Sox2 mcherry + : Mean+StdDev<br><br>**Population : Nuclei Selected**<br>whole_nuclei : Mean<br><br>**Population : Nuclei**<br>Number of Objects<br><br>**Population : Sox2 +**<br>Number of Objects<br>Intensity Nucleus Alexa 488 Mean : Mean<br><br>**Population : mcherry +**<br>Number of Objects<br>Intensity Nucleus Alexa 555 Mean : Mean<br><br>**Population : nestin +**<br>Number of Objects<br>Intensity Nucleus Alexa 647 Mean : Mean<br><br>**Population : Sox2 mcherry +**<br>Number of Objects<br><br>**Population : Nestin + mCherry+**<br>Number of Objects
	**Object Results**<br>Population : Image Region nestin : None<br>Population : whole_nuclei : Use Selected Well Results<br>Population : Nuclei Selected : None<br>Population : Nuclei : None<br>Population : Sox2 + : None<br>Population : mcherry + : None<br>Population : nestin + : None<br>Population : Sox2 mcherry + : None<br>Population : Nestin + mCherry+ : None

6. Image analysis pipeline in Harmony

Related to ED Fig. 2g

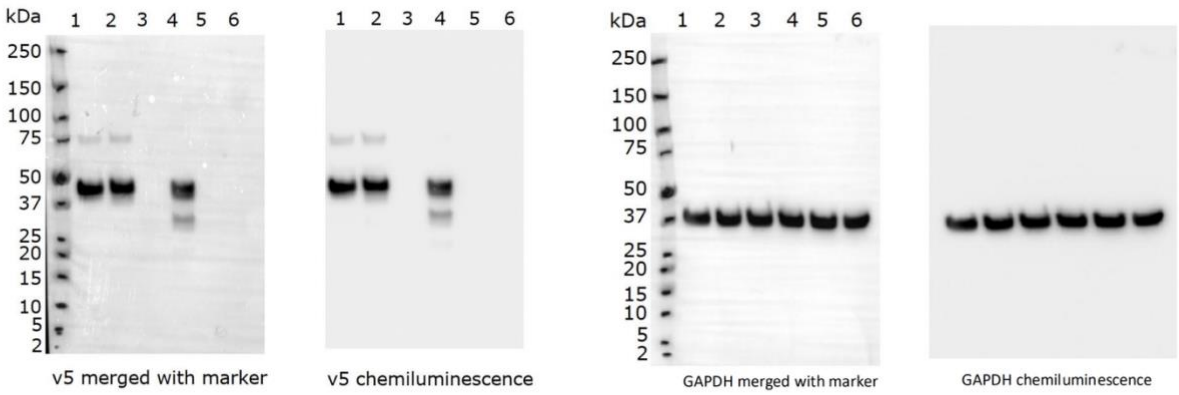

Related to ED Fig. 10d

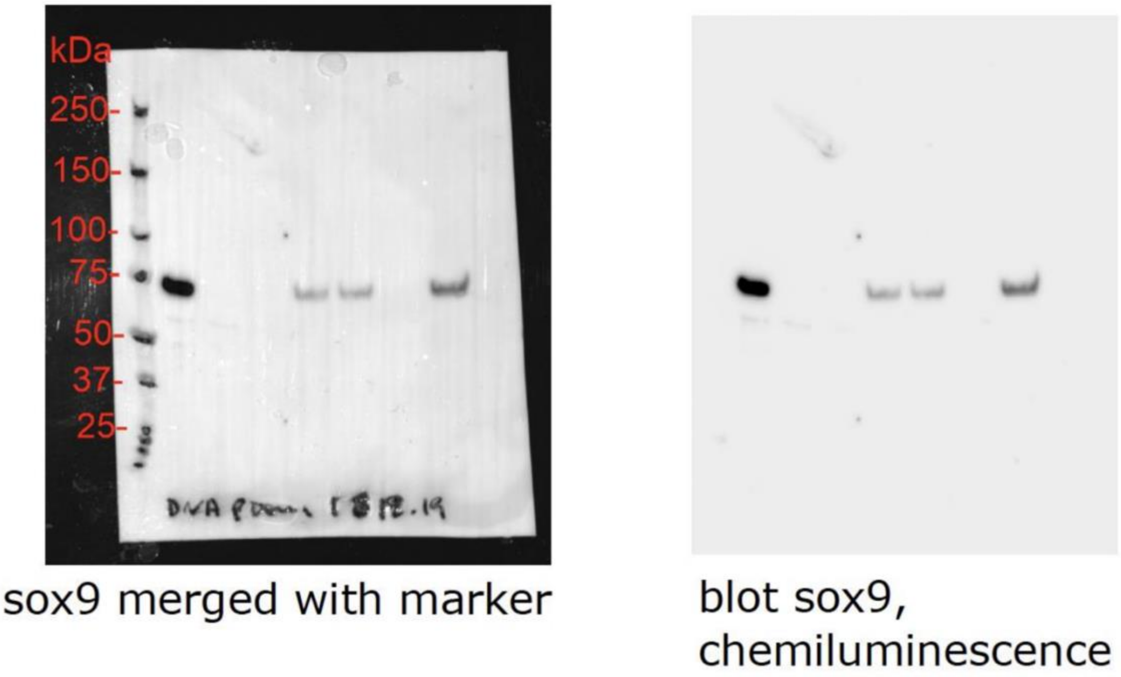

7. Western blots uncropped gels

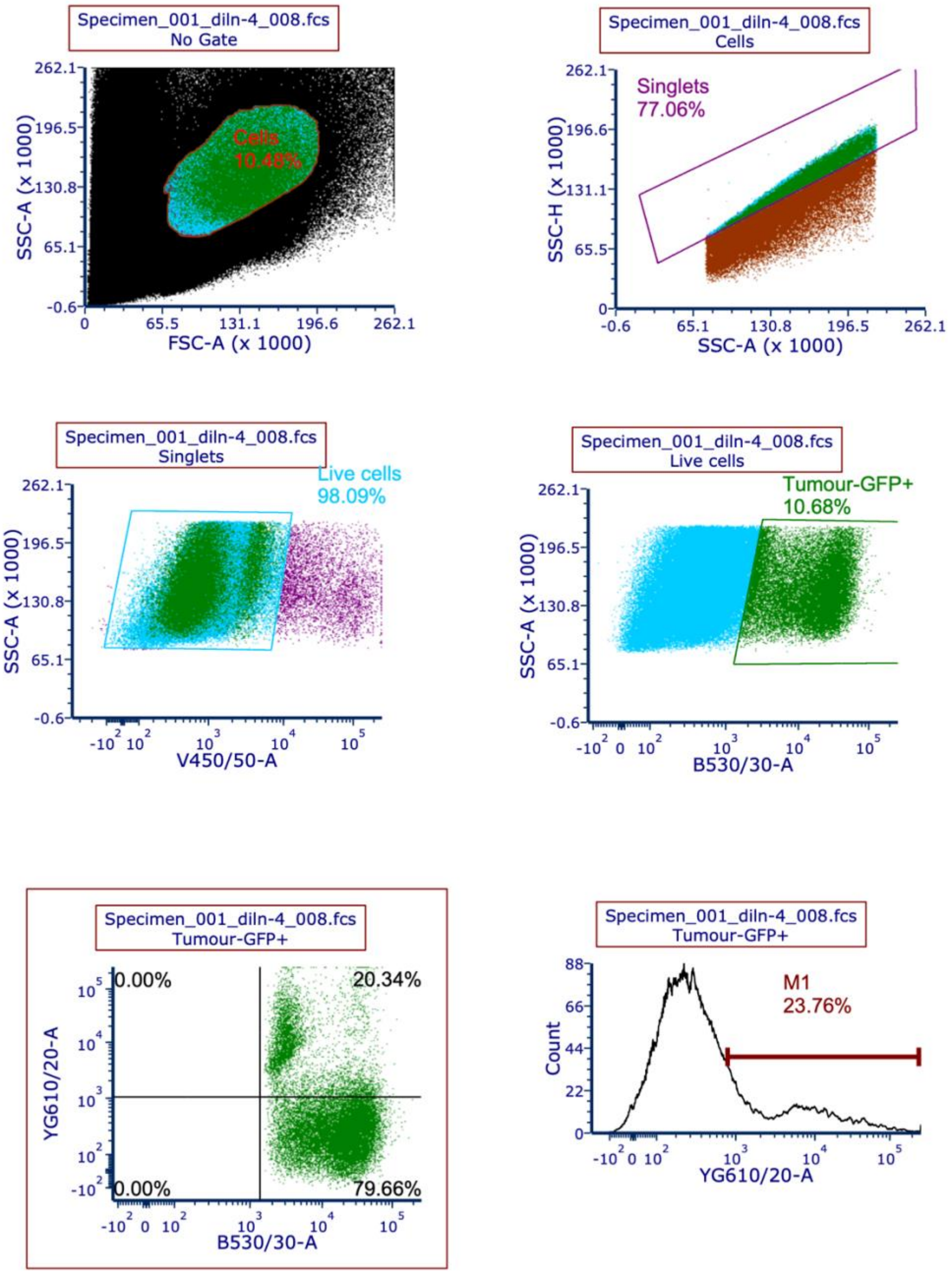

8. Typical example of flow gating for all tumours processed in Extended Data 13j
