# Supplementary material for: Synthetic super-enhancers enable precision viral immunotherapy
Source: Nature. 2026 Apr 8;653(8113):232–41. doi: 10.1038/s41586-026-10329-6 (PMC13149004; doi:10.1038/s41586-026-10329-6)
Supplement: Supplementary file 2 — Reporting Summary [file 41586_2026_10329_MOESM2_ESM.pdf]

nature portfolio

Corresponding author(s): Steven Pollard
Last updated by author(s): 18/02/2026

Reporting Summary

Nature Portfolio wishes to improve the reproducibility of the work that we publish. This form provides structure for consistency and transparency in reporting. For further information on Nature Portfolio policies, see our Editorial Policies and the Editorial Policy Checklist.

Statistics

For all statistical analyses, confirm that the following items are present in the figure legend, table legend, main text, or Methods section.

n/a	Confirmed	
☐	☒	The exact sample size (*n*) for each experimental group/condition, given as a discrete number and unit of measurement
☐	☒	A statement on whether measurements were taken from distinct samples or whether the same sample was measured repeatedly
☐	☒	The statistical test(s) used AND whether they are one- or two-sided<br>*Only common tests should be described solely by name; describe more complex techniques in the Methods section.*
☐	☒	A description of all covariates tested
☐	☒	A description of any assumptions or corrections, such as tests of normality and adjustment for multiple comparisons
☐	☒	A full description of the statistical parameters including central tendency (e.g. means) or other basic estimates (e.g. regression coefficient) AND variation (e.g. standard deviation) or associated estimates of uncertainty (e.g. confidence intervals)
☐	☒	For null hypothesis testing, the test statistic (e.g. *F*, *t*, *r*) with confidence intervals, effect sizes, degrees of freedom and *P* value noted<br>*Give P values as exact values whenever suitable.*
☒	☐	For Bayesian analysis, information on the choice of priors and Markov chain Monte Carlo settings
☒	☐	For hierarchical and complex designs, identification of the appropriate level for tests and full reporting of outcomes
☒	☐	Estimates of effect sizes (e.g. Cohen's *d*, Pearson's *r*), indicating how they were calculated

Our web collection on statistics for biologists contains articles on many of the points above.

Software and code

Policy information about availability of computer code

Data collection	- Sequencing data was collected using the Illumina NovaSeq platform.<br>- Western blots and EMSAs were imaged using BioRAD ChemiDoc MP imaging system. (Software version: 2.4.0.03).<br>- Fluorescent immunostaining images were captured by Nikon Eclipse TiE microscope (NIS elements software)<br>- Incucyte Imaging system for live cell imaging (Inucyte Base Software)<br>- Opera Phenix for tissue imaging of immunostaining (Harmony software, Version 5.2).<br>- DNA fragment sizes were measure by Agilent 2200 Tapestation.<br>- For flow cytometery, a BD LSRFortessa cell analyser was used. Data acquisition and analysis were conducted using the FCS Express 7 (7.24.0030) or FlowJo (version 10.6.2)<br>- For luciferase assays plate screening was performed using the Ensight Multimode Plate Reader, Perkin Elmer.<br>-IVIS imaging system (PerkinElmer) for bioluminescence imaging of tumours in live mice.

Data analysis	Data was analyzed using the following open source and commercial softwares: FASTQC v0.11.8, Bowtie v2.3, Bedtools V2.28, Picard v2.20, MACS v2.1.1, DeepTools V2, SAMTool2 v1.3.1, BEDOPS V2.49, MEME v5.0.2, R v3.6 (various R packages as indicated in the methods), IGV version 2.13.2 run with JAVA 11.0.13 (OpenJDK 64-bit). ImageJ (v1.54f) and Java 1.8.0_322 (64-bit), Graphpad Prism)Version 10.6.1).<br><br>Custom scripts were deposited in UOE GitHub:<br>https://github.com/alhafidzhamdan/sse_gene_therapy/tree/main

For manuscripts utilizing custom algorithms or software that are central to the research but not yet described in published literature, software must be made available to editors and reviewers. We strongly encourage code deposition in a community repository (e.g. GitHub). See the Nature Portfolio guidelines for submitting code & software for further information.

Data

Policy information about availability of data

All manuscripts must include a data availability statement. This statement should provide the following information, where applicable:

- Accession codes, unique identifiers, or web links for publicly available datasets
- A description of any restrictions on data availability
- For clinical datasets or third party data, please ensure that the statement adheres to our policy

The single cell RNA-seq data has been deposited at the ENA. Accession number: PRJEB81816. The project number for ChIP-seq data is PRJEB107008.
Data:
For ChIP-seq, raw (fastq) and processed (bigwig and narrowPeak) files have been deposited and made public at https://www.ebi.ac.uk/ena/browser/view/PRJEB107008
ENA does not take processed files so these have been uploaded to BioStudies which will link it to the same ENA accession number PRJEB107008, otherwise they are at https://www.ebi.ac.uk/biostudies/studies/S-BSST2733

Repo:
https://github.com/alhafidzhamdan/sse_gene_therapy
GitHub repo has been updated to include some processed files including all the narrowPeak files and consensus sets; using these in conjunction with the .Rmd document people should be able to reproduce Figure 2.
Also included a link to the SOX2 and SOX9 tracks on UCSC
Linked and minted a repo on Zenodo - https://zenodo.org/records/18676096

Research involving human participants, their data, or biological material

Policy information about studies with human participants or human data. See also policy information about sex, gender (identity/presentation), and sexual orientation and race, ethnicity and racism.

Reporting on sex and gender	N/A
Reporting on race, ethnicity, or other socially relevant groupings	N/A
Population characteristics	N/A
Recruitment	N/A
Ethics oversight	N/A

Note that full information on the approval of the study protocol must also be provided in the manuscript.

Field-specific reporting

Please select the one below that is the best fit for your research. If you are not sure, read the appropriate sections before making your selection.

☒ Life sciences ☐ Behavioural & social sciences ☐ Ecological, evolutionary & environmental sciences

For a reference copy of the document with all sections, see nature.com/documents/nr-reporting-summary-flat.pdf

Life sciences study design

All studies must disclose on these points even when the disclosure is negative.

Sample size	Sample sizes are indicated in each Figure legend. Generally, three to six biological replicates were used. This was determined according to established methods in the field and previous experience such as that in (Gangosa et al., using the NPE-IE mouse model PMID: 33857425 or Pollard et al.,PMID: 19497285), which allowed us to predetermine the number of sample size of each experiment for GSC lines or mice.

	The number of ChIP-seq peaks were identified to be significantly enriched over input by MACS2 software (q = 0.01). All sequencing libraries were normalized to sequencing depth.
Data exclusions	No data were excluded. All experiment included positive, negative or internal controls where appropriate.
Replication	Each Experiment was repeated as indicated in the figure legends and the Methods section.
Randomization	All mice bearing tumours for treatment were randomly allocated to groups for experimental versus control arms following IVIS imaging to confirm tumour burden.
Blinding	No blinding was required.

Reporting for specific materials, systems and methods

We require information from authors about some types of materials, experimental systems and methods used in many studies. Here, indicate whether each material, system or method listed is relevant to your study. If you are not sure if a list item applies to your research, read the appropriate section before selecting a response.

Materials & experimental systems

n/a	Involved in the study	
☐	☒	Antibodies
☐	☒	Eukaryotic cell lines
☒	☐	Palaeontology and archaeology
☐	☒	Animals and other organisms
☒	☐	Clinical data
☒	☐	Dual use research of concern
☒	☐	Plants

Methods

n/a	Involved in the study	
☐	☒	ChIP-seq
☐	☒	Flow cytometry
☒	☐	MRI-based neuroimaging

Antibodies

Antibodies used

All antibodies for immune profiling are listed in the Supplementary information.

Antibodies used are described in the supplementary information (for Fig. 5 immune characerisation) or in the methods. They are listed here as well:

For westerns and immunostaining cells and tissues:

Sox2 (rabbit) 1:200 Abcam Ab92494
Sox2 (goat) 1:200, R&D Systems af2018
Olig2 (rabbit) 1:200 Sigma (Ab9610)
Nestin (mouse) 1:500 R&D Systems (mab1259)
GFAP (mouse) 1:500 Millipore (ab5541)
SOX9 (rabbit) 1:200 Millipore (ab5535)
V5 (mouse IgG2b) 1:1000 eBioscience (14679682)
Rabbit secondary (goat). 1:1000 Alexa 488 Invitrogen (A11008)
Rabbit secondary (goat) 1:1000 Alexa 594 Invitrogen (A11012)
Goat secondary (donkey) 1:1000 Alexa 488 Invitrogen (A11055)
Mouse IgG secondary (goat) 1:1000. Alexa 594 Invitrogen (A21044)

For immune profiling

NK1.1 BD Biosciences PK136/Cat #564144 1/300
CD4 BD Biosciences GK1.5/ Cat# 564667
1/300
CD45 BD Biosciences 30-F11/ Cat# 565710
1/200
CD11B BD Biosciences M1/70/ Cat# 565080
1/200
CD3 BD Biosciences 17A2/Cat# 564380
1/300
CD8a BD Biosciences 53-67/ Cat# 612898 1/300
FOXP3 eBioscience FJK-16S/ Cat#48577382 1/100
CD62L BD Biosciences MEL-14/ Cat#563117 1/400
TIM-3 Biolegend RMT-23/Cat#119721 1/100
CD27 Biolegend LG.3A10/Cat# 119721 1/200
GITR BD Biosciences DTA-1/Cat# 563390 1/200
CD44 Biolegend IM7/ Cat#103059 1/200
CD25 Biolegend PC61/Cat#102017 1/200

LAG3 eBioscience ebioC9B7W/Cat#46-2231-82 1/100
TCF7 Cell Signalling Technology C63D9/ Cat#14456S 1/100
PD-1 BD Biosciences J43/ Cat#562523 1/200
Ki67 eBioscience SolA15/ Cat# 25-5698-82 1/200
TOX Miltenyi REA473/ Cat#130-118-335 1/100
Granzyme b BD Biosciences GB11/ Cat#560213 1/200
CD24 BD Biosciences M1/69/ Cat# 564664 1/400
LY6G BD Biosciences IA8/Cat# 565707 1/100
CD45 BD Biosciences 30F11/Cat# 624287 1/400
CD68 Biolegend FA-11/Cat#137017 1/200
Cd16.2 Biolegend 9E9/Cat #149531 1/200
LY6C BD Biosciences AL-21/Cat# 563011 1/200
CD206 Biolegend C068C2/Cat#141723 1/400
MHCII Biolegend M5-114-15-2/Cat#107643 1/200
CD11c Biolegend N418/Cat#117336 1/100
CD49D Biolegend R1-2/Cat#103611 1/200
XCR1 Biolegend ZET/Cat#148208 1/200
CD16 Biolegend S17014E/Cat#158003 1/200
PDL1 Biolegend 10F.9G2/Cat# 124324 1/200
CD64 Biolegend X54-5/7.1/Cat#139314 1/200
CD32b Thermo Scientific AT130-2/Cat# 17-0321-82 1/200
Ki67 nvitrogen SolA15/Cat# 56-5698-82 1/200
IFN-gammaBiolegend XMG1.2/Cat#505813 1/100
IL-2 eBiosciences JES6-5H4/Cat#17-7021-82 1/100
TNF-alpha Invitrogen MP6-XT22/Cat#25-7321-82 1/100

Validation

All antibodies were obtained from commercial sources and were validated by the company; refer to the company website for detailed validation analysis.

Eukaryotic cell lines

Policy information about cell lines and Sex and Gender in Research

Cell line source(s)

The study uses many primary patient derived cell lines and long term cultures.

Primary GBM and NSC cell lines were used (from the Glioma Cellular Genetics Resource, Pollard lab in preparation). These were all STR profiled and mycoplasma screened. They were all validated as tumour-intiating, with varying kinetics. Primary human fibroblasts were genearated from primary tissue biopsies. HEK293 (Thermo).

The mouse NPE-IE cells were described by Gangoso et al (Cell, 2021) and are primary mouse adult neural stem cells engineered with key GBM driver mutations and serially passaged through BL6 mice (tertiary transplants and rederived cultures).

iPSC derived neurons and microglia were generated from commercial sources (bit.bio) following their experimental protocols.

Authentication

GSCs and NSCs were STR profiled and are novel primary cell lines.
HEK293 was supplied directly from Thermo already validated.
The iPSC differentiated derivative from Bit.bio were validated as per company product sheets.

Mycoplasma contamination

All cells were routinely checked for Mycoplasma contamination and tested negative.

Commonly misidentified lines
(See ICLAC register)

No misidentified cell lines were used in this study.

Animals and other research organisms

Policy information about studies involving animals; ARRIVE guidelines recommended for reporting animal research, and Sex and Gender in Research

Laboratory animals

C57BL/6 J mice were used to generate tumours.

Wild animals

no wild animals were used in the study.

Reporting on sex

Sex information is provided in the methods and figures legends.

Field-collected samples

no field collected samples were used in the study.

Ethics oversight

Local Animal Welfare and Ethical Review Body (AWERB) (Univeristy of Edinburgh).

All animal experiments were reviewed and approved by the University of Edinburgh Animal Welfare and Ethical Review Body

(AWERB) and were conducted in accordance with the Animals (Scientific Procedures) Act 1986 under UK Home Office Project Licence [PP8631583. All procedures complied with institutional guidelines and regulations.

Note that full information on the approval of the study protocol must also be provided in the manuscript.

Plants

Seed stocks

N/A

Novel plant genotypes

N/A

Authentication

N/A

ChIP-seq

Data deposition

☒ Confirm that both raw and final processed data have been deposited in a public database such as GEO.

☒ Confirm that you have deposited or provided access to graph files (e.g. BED files) for the called peaks.

Data access links
May remain private before publication.

https://www.ebi.ac.uk/ena/browser/view/PRJEB107008
https://www.ebi.ac.uk/biostudies/studies/S-BSST2733

Files in database submission

E17_Sox2_peaks.narrowPeak
E21_Sox2_peaks.narrowPeak
E27_Sox2_peaks.narrowPeak
E28_Sox2_peaks.narrowPeak
E31_Sox2_peaks.narrowPeak
E34_Sox2_peaks.narrowPeak
E37_Sox2_peaks.narrowPeak
E17_Sox9_peaks.narrowPeak
E21_Sox9_peaks.narrowPeak
E27_Sox9_peaks.narrowPeak
E28_Sox9_peaks.narrowPeak
E31_Sox9_peaks.narrowPeak
E34_Sox9_peaks.narrowPeak
E37_Sox9_peaks.narrowPeak
E17_Sox2_sorted_merged.bw
E21_Sox2_sorted_merged.bw
E27_Sox2_sorted_merged.bw
E28_Sox2_sorted_merged.bw
E31_Sox2_sorted_merged.bw
E34_Sox2_sorted_merged.bw
E37_Sox2_sorted_merged.bw
E17_Sox9_sorted_merged.bw
E21_Sox9_sorted_merged.bw
E27_Sox9_sorted_merged.bw
E28_Sox9_sorted_merged.bw
E31_Sox9_sorted_merged.bw
E34_Sox9_sorted_merged.bw
E37_Sox9_sorted_merged.bw

Genome browser session
(e.g. UCSC)

https://genome.ucsc.edu/s/alhafidzhamdan/co_bound_SOX2_SOX9_peaks

Methodology

Replicates

Three ChIP replicates were pooled to make a DNA library for each ChIP-seq experiment and two independent replicates were carried out. All other sequencing data were carried out in duplicates or a pool of triplicates.

Sequencing depth

Around 50-60 million pair-end reads were obtained on average from each ChIP-seq.

Antibodies

R&D Sox2 #AF2018
Millipore Sox9 #AB5535

Peak calling parameters	Duplicates were removed from the aligned pair-end BAM files using Picard prior to peak calling. TF peaks (sample files) showing significant enrichment over input DNA (control files) obtained from the same cells were called using MACS2 (version 2.1.1.20160309) and a fragment size of 200 bp (--nomodel --extsize 200) and were controlled to q value (minimum FDR) cut-off of 0.01 (-q 0.01). The peaks that overlapped with the ENCODE mm9 blacklist were removed using the bedtools intersect function (flag –v).<br>To identify broadPeaks of TF binding, peaks were called as using MACS2 with the following flags: -B --broad-cutoff 0.1 --broad --nomodel --extsize 200. Regions that overlapped with the ENCODE blacklist were removed using the bedtools intersect function (flag –v).
Data quality	Quality controls of DNA libraries were carried out by DNA fragment size distribution using Tapestation and Bioanalyzer (Agilent). Sequencing quality was assessed by mean quality scores using FASTQC and only Phred scores above 30 were considered. Sequence duplication and library complexity was assessed by MutiQC and Qualimap prior to further analysis. Duplicates were removed by Picard and adapters by Cutadapt. Libraries were normalized by squencing depth to 1X genome coverage using DeepTools.
Software	FASTQC v0.11.8, MultiQC v1.3, Bowtie v2.3, Bedtools V2.28, Picard v2.20, MACS v2.1.1, DeepTools V2, Qualimap V2.2.1, SAMTool2 v1.3.1, MEME v5.0.2, R v3.6 (various R packages as indicated in the methods), BEDOPS V2.49 and Cutadapt v3.3.

Flow Cytometry

Plots

Confirm that:

☒ The axis labels state the marker and fluorochrome used (e.g. CD4-FITC).

☒ The axis scales are clearly visible. Include numbers along axes only for bottom left plot of group (a 'group' is an analysis of identical markers).

☒ All plots are contour plots with outliers or pseudocolor plots.

☒ A numerical value for number of cells or percentage (with statistics) is provided.

Methodology

Sample preparation	Cells were first dissociated using Accutase. Following this, the cells were centrifuged and washed twice with phosphate-buffered saline (PBS) to ensure the removal of any residual trypsin and medium. The washed cells were then resuspended in PBS for subsequent analysis.
Instrument	Flow cytometric analysis was performed using a Beckman Coulter BD LSRFortessa cell analyser was used.
Software	Data acquisition and analysis were conducted using the FlowJo or FCS Express 7 cytometry software.
Cell population abundance	The fluorescent markers eGFP and mCherry were used to score tumour cells (NPE-IE cells) or viral transduction and promoter activity, respectively.
Gating strategy	To remove dead cells, all samples were initially gated using the FSC-A/SSC-A gating to identify the live cell population (below 200 FS Area). To remove cell doublets, single cells were selected by gating forward scatter height vs area. The positively fluorescent cells were gated based on the fluorescent intensity of positive control cells.

☒ Tick this box to confirm that a figure exemplifying the gating strategy is provided in the Supplementary Information.
